# Blood transcriptomics of drug-naïve sporadic Parkinson’s disease patients

**DOI:** 10.1186/s12864-015-2058-3

**Published:** 2015-10-28

**Authors:** Raffaella Calligaris, Mihaela Banica, Paola Roncaglia, Elisa Robotti, Sara Finaurini, Christina Vlachouli, Lucia Antonutti, Francesco Iorio, Annamaria Carissimo, Tatiana Cattaruzza, Andrea Ceiner, Dejan Lazarevic, Alberto Cucca, Nicola Pangher, Emilio Marengo, Diego di Bernardo, Gilberto Pizzolato, Stefano Gustincich

**Affiliations:** Area of Neuroscience, International School for Advanced Studies (SISSA), via Bonomea 265, 34136 Trieste, Italy; Department of Medical Sciences, Neurology Unit, University of Trieste, Strada di Fiume 447, 34100 Trieste, Italy; Department of Environmental and Life Sciences, University of Eastern Piedmont, Viale T. Michel 11, 15121 Alessandria, Italy; Telethon Institute of Genetics and Medicine (TIGEM), Via P. Castellino 111, Naples, 80131 Italy; ITALTBS S.p.A., AREA Science Park, Padriciano, 99, 34149 Trieste, Italy; CBM Scrl - Consorzio per il Centro di Biomedicina Molecolare, Area Science Park, S.S.14, km 163.5, Basovizza, 34149 Trieste, Italy; Department Computer Science & Systems, School of Engineering, University of Naples “Federico II”, via Claudio 21, 80125 Naples, Italy; Present Address: European Molecular Biology Laboratory, European Bioinformatics Institute (EMBL-EBI), CB10 1SD Hinxton, Cambridge, UK

**Keywords:** Blood transcriptomics, Parkinson’s disease, *de novo*, Drug-naïve patients

## Abstract

**Background:**

Parkinson’s disease (PD) is a chronic progressive neurodegenerative disorder that is clinically defined in terms of motor symptoms. These are preceded by prodromal non-motor manifestations that prove the systemic nature of the disease. Identifying genes and pathways altered in living patients provide new information on the diagnosis and pathogenesis of sporadic PD.

**Methods:**

Changes in gene expression in the blood of 40 sporadic PD patients and 20 healthy controls ("Discovery set") were analyzed by taking advantage of the Affymetrix platform. Patients were at the onset of motor symptoms and before initiating any pharmacological treatment. Data analysis was performed by applying Ranking-Principal Component Analysis, PUMA and Significance Analysis of Microarrays. Functional annotations were assigned using GO, DAVID, GSEA to unveil significant enriched biological processes in the differentially expressed genes. The expressions of selected genes were validated using RT-qPCR and samples from an independent cohort of 12 patients and controls ("Validation set").

**Results:**

Gene expression profiling of blood samples discriminates PD patients from healthy controls and identifies differentially expressed genes in blood. The majority of these are also present in dopaminergic neurons of the Substantia Nigra, the key site of neurodegeneration. Together with neuronal apoptosis, lymphocyte activation and mitochondrial dysfunction, already found in previous analysis of PD blood and post-mortem brains, we unveiled transcriptome changes enriched in biological terms related to epigenetic modifications including chromatin remodeling and methylation. Candidate transcripts as CBX5, TCF3, MAN1C1 and DOCK10 were validated by RT-qPCR.

**Conclusions:**

Our data support the use of blood transcriptomics to study neurodegenerative diseases. It identifies changes in crucial components of chromatin remodeling and methylation machineries as early events in sporadic PD suggesting epigenetics as target for therapeutic intervention.

**Electronic supplementary material:**

The online version of this article (doi:10.1186/s12864-015-2058-3) contains supplementary material, which is available to authorized users.

## Background

Parkinson’s disease (PD) is a slowly progressive degenerative disorder of the central nervous system (CNS) that is classically defined in terms of motor symptoms consequent to degeneration of specific subsets of mesencephalic dopaminergic (DA) cells within *Substantia Nigra* (SN) *pars compacta*. Although DA drugs are effective in alleviating motor symptoms in PD patients, no pharmacological treatment is currently available to slow or arrest the neurodegenerative process. Furthermore, accurate early diagnosis suffers from the lack of reliable biomarkers. This is due at least in part to three challenges. First, at the onset of the motor symptoms, dopamine depletion in the *putamen* is 80 %, with 60 % of the SN DA neurons already lost [1], proving that at the time of clinical trials neuronal networks are already largely compromised. Moreover, PD is not a homogeneous disease since it presents a plethora of different clinical forms with unclear molecular differences and consequences on the treatment of choice. Finally, the aetiology and the initial molecular events of the disease remain unknown since the injured tissue in living patients is not accessible to genomics and biochemical analysis.

PD presents a variety of neuropsychiatric, autonomic, sensory, and sleep disorders that may precede the expression of motor disturbances by more than a decade suggesting that PD is a systemic disease [2]. In this context, a long and intriguing list of alterations of blood physiology has been described in PD patients [3].

Gene expression profiles represent a powerful tool to study the molecular basis of a systemic disease in living patients. Many proof − of − concept studies have been reported to use them as surrogates for disease prediction and classification [4]. Recently, gene expression analysis has identified changes in blood correlated to neurodegeneration in PD [5–11]. However, some of these works suffer the variability derived from enrolling patients in different stages of the disease and from the unknown effects of pharmacological treatments.

Here we present the largest study to date of sporadic PD patients at the early stage of the disease (*de novo*) and before any specific pharmacological treatment (drug-naïve) to perform gene expression profiling using Affymetrix microarrays on peripheral blood samples.

We show early changes in genes and pathways that provide new candidates on the quest for peripheral biomarkers of PD for diagnosis and patients’ classification. Furthermore, we confirm differences in expression for biological pathways and selected genes previously identified in both PD blood and *post-mortem* brains increasing their significance as peripheral biomarkers. Together with the expected alteration in biological terms comprising neuronal apoptosis, mitochondrial dysfunction and inflammation, we have also found enrichment in genes involved in chromatin remodeling suggesting new strategies for pharmacological intervention.

## Methods

### Subjects

The study was approved by the Ethical Committee at the Movement Disorders Center of the Neurology Clinic, Azienda Ospedaliero-Universitaria Ospedali Riuniti, Trieste, Italy. Study participants gave written informed consent. During a two-year period we enrolled 52 patients with a first clinical diagnosis of PD, according to the UK Parkinson’s Disease Society Brain Bank criteria. Thirty-two healthy age- and ethnicity-matched control subjects (HC) were also included in the study. The “Discovery set” of the experiment included 40 PD patients (68.8 years ± 6.9 SD) and 20 HC (60.3 years ± 5.7 SD). Subjects of the “Validation set” included 12 PD (68.8 years ± 5.2 SD) and 12 HC (68.0 years ± 1.5 SD) volunteers. The demographic, clinical and haematological characteristics of the two study groups are shown in Table 1. *De novo* and drug-naïve PD patients were the selected cohort of this study. While both sustained denervation at the nigrostriatal dopaminergic axis, treatment with levodopa or dopamine agonist might interfere with different central neurotransmitter pathways influencing gene expression profiles. Therefore enrolled subjects did not take any centrally acting drugs in the previous 6 months. Although no genetic testing was performed, any family history of PD was determined by self-report and review of medical records if available was used as an exclusion criterion in order to minimize the inclusion of genetic forms of the disease. Inclusion criteria for HCs were no personal or familiar history of any neurological and psychiatric disorder.

**Table 1:** Demographics, clinical phenotypes and haematological values for *de novo* and drug-naïve PD patients and healthy controls

Diagnostic group	Parkinson’s disease		Controls	
	Discovery set	Validation set	Discovery set	Validation set
Number	40	12	20	12
Gender (Male/Female), number	22/18	6/6	10/10	5/7
Age, yr, mean (SD; range)	68.83 (6.94; 51–78)	68.82 (5.22; 59–76)	60.30 (5.69; 53–69)	68.00 (1.5; 65–71)
Age at symptoms onset, yr, mean (SD; range)	67.65 (6.80; 50–76)	67.63 (5.05; 58–74)	NA	NA
Symptom duration, yr, mean (SD; range)	1.14 (0.66; 0.5–3)	1.49 (0.85; 0.5–3)	NA	NA
Clinical phenotype at enrollment:				
Unilateral symptoms, number (%)	24 (60)	10 (83.3)	NA	NA
Bilateral symptoms, number (%)	16 (40)	2 (16.7)	NA	NA
Tremor, number (%)	7 (17.5)	3 (25.0)	NA	NA
Bradykinesia/rigidity, number (%)	10 (25.0)	3 (25.0)	NA	NA
Mixed, number (%)	23 (57.5)	6 (50.0)	NA	NA
Hoehn and Yahr stage, mean (SD; range)	1.45 (0.55; 1–2)	1.50 (0.71; 1–3)	NA	NA
UPDRS motor score, mean (SD; range)	14.72 (8.15; 3–29)	13.82 (7.22; 1–24)	NA	NA
MMSE score, mean (SD; range)	29.77 (0.48; 29–30)	29.73 (0.90; 27–30)	NA	NA
^123^I-FP-CIT SPECT abnormality:				
Type 1, number (%)	21 (55.3)	6	NA	NA
Type 2, number (%)	17 (44.7)	5	NA	NA
Not executed, number	2	1	NA	NA
Haematological values (mean, SD):				
White blood cells [10^A^3/^L]	6.19 (1.58)	4.97 (1.47)	5.76 (1.24)	5.78 (1.09)
Neutrophils (%)	61.67 (8.18)	59.35 (14.64)	57.52 (6.18)	57.74 (5.85)
Lymphocytes (%)	27.85 (7.49)	28.92 (2.72)	31.31 (6.52)	30.63 (6.21)
Monocytes (%)	7.40 (1.73)	8.50 (2.72)	7.38 (1.74)	8.73 (2.42)
Eosinophils (%)	2.62 (1.52)	2.60 (1.89)	3.17 (1.81)	2.42 (1.24)
Basophils (%)	0.49 (0.16)	0.67 (0.36)	0.63 (0.26)	0.63 (0.20)
Red blood cells [10A6/^L]	4.59 (0.49)	4.44 (0.55)	4.79 (0.44)	4.74 (0.44)
Hemoglobin [g/dL]	14.40 (1.23)	14.28 (1.49))	14.14 (0.88)	14.85 (0.52)
MCV fL]	91.44 (4.61)	94.05 (3.03)	88.56 (4.54)	94.13 (5.37)
MCH [pg]	31.53 (2.42)	32.35 (1.47)	30.16 (1.77)	32.08 (2.10)
MCHC [g/dl]	34.46 (1.22)	34.37 (0.59)	34.05 (0.76)	34.05 (0.51)
Platelet count [10A3/^L]	226.71 (66.72)	176.33 (14.87)	221.60 (43.62)	226.92 (78.05)

*NA* not applicable, *Yr* year, *UPDRS* Unified Parkinson’s Disease Rating Scale, *MMSE* Mini-Mental State Examination

### Clinical assessment

Data referring to detailed history of the disease symptoms, co-morbid conditions, previous drug intake, and any evidence of family history of neurological diseases were collected. After a standardized neurological examination, Parkinsonian symptomatology was assessed by the motor subsection of the Unified Parkinson’s Disease Rating Scale, UPDRS-motor part [12], and the Hoehn and Yahr staging scale [13]. Patients’ cognition was assessed by the Mini-Mental State Examination (MMSE) [14]. Patients also underwent brain Computed Tomography and Magnetic Resonance Image scanning and Single Photon Emission Computed Tomography (SPECT) imaging with the pre-synaptic DA ligand ^123^I-2β-carbometoxy-3β-(4-iodophenyl)-N-(3-fluoropropyl) nortropane (^123^I-FP-CIT) to assess the loss of nigrostriatal terminals and to gain a functional picture of the progression of the degenerative process within nigral DA neurons. SPECT images were classified as normal (symmetric bilateral uptake of the basal ganglia regions) or abnormal by visual inspection of an experienced Nuclear Medicine Specialist. Abnormal scans were graded as follow: asymmetric uptake with normal or almost normal putamen activity in one hemisphere and more marked changes on the other side (type 1); greatly reduced uptake in the putamen on both the right and left sides (type 2); very low uptake in the basal ganglia regions with increased specific background signal (type 3) [15].

### Blood collection, RNA purification and microarray processing

Blood samples were harvested directly and sequentially into 8 PAXgene Blood RNA tubes (PreAnalytiX, Hombrechtikon, CH) via a 21 − gauge butterfly needle and then frozen and kept at −80 °C. Total RNA was purified using PAXgene™ Blood RNA kit (PreAnalytiX GmbH, Qiagen, Hilden, Germany) and DNaseI treatment was performed by ‘on-column’ treatment as recommended by manufacturer’s instructions plus a second treatment subsequent to elution. RNA was then purified using RNeasy (Qiagen, Hilden, Germany) and quantified by NanoDrop ND-100 Spectrophotometer (NanoDropTechnologies; Wilmington, DE). RNA integrity was determined with 2100 Bioanalyzer (Agilent Technologies, Palo Alto, CA) and exclusively samples with RIN ≥ 8 were included in the subsequent investigations. Hybridization targets were synthesized with Ovation™ Whole Blood Solution (NuGEN) after comparison with other 2 methods (Additional file 1) and hybridized to HG − U133A 2.0 arrays (Affymetrix, Santa Clara, CA), investigating the expression of 18400 transcripts.

### Data analysis

Principal Component Analysis (PCA) [16] was employed to reduce the dimensionality of the dataset before the application of classification methods. Partial Least Square Discriminant Analysis (PLS-DA) [17] was applied for classification purposes to obtain a first selection of the discriminating variables by using a binary coded Y variable (−1 for control samples and +1 for pathological samples). A preliminary application of PLS-DA reduced the number of relevant variables to 1612. Only classification models with a maximum of 6 Principal Component (PCs) were considered (Additional file 2). Ranking-Principal Component Analysis (R-PCA) [18] ranked variables according to their decreasing discriminant ability. Linear Discriminant Analysis (LDA) [19], a Bayesian classification method, provided the classification of the samples considering the multivariate structure of the data. Here, a Forward Selection procedure [19] was applied to the principal components. The classification performance of the models was evaluated by the non-error rate (NER%), namely the percentage of overall correct assignments. Further data processing was performed in the R computing environment (http://www.r-project.org/) version 2.8.0 with BioConductor packages (http://www.bioconductor.org/). Data were first filtered by eliminating probes with detection call of poor quality as well as those with intensity value lower than log_2_100 for all the samples. Of the original 60 samples, one (a control samples) did not pass the microarray hybridization quality controls and was excluded from further analyses. Therefore, the final dataset consisted of 59 samples described by 15137 probes.

Robust Multi-Array Average (RMA) normalization was applied to microarray data and these were imported in the Multiexperiment Viewer (MeV) software version 4.5.1 for Windows XP (http://www.tm4.org/mev.html). Statistical analysis was performed with PUMA [20], SAM (Significance Analysis of Microarrays) [21] and Rank Product (RP) modules [22] to detect significantly differentially expressed genes.

PUMA is a Bayesian method (available in R BioConductor) that includes probe-level measurement error into the estimates of expression profile [20]. These were normalized through a median global array scaling, and a single expression value for each condition was combined from the replicates and associated to a probability of positive log ratio (PPLR) between conditions. In order to facilitate the interpretation of results, PPLR was converted in a p-value-like form: 1-PPLR was used for up-regulated genes while PPLR for down-regulated ones. SAM was chosen for its power to allow the control of false positive results (False Discovery Rate or FDR). This is particularly relevant when looking at human samples because of the inherent rate of genetic variation among individuals. Data were filtered so that only probe sets that had a Present call and intensity value of >100 in at least half the arrays of the smaller group were retained.

Functional analyses were performed using Gene Ontology (GO) annotations [23], DAVID Bioinformatics Resources [24] and Gene Set Enrichment Analysis (GSEA) [25] as implemented at http://www.broadinstitute.org/gsea/, version 2.06.

### Quantitative real-time PCR (RT-qPCR)

Total RNA was reverse-transcribed using Superscript III Reverse Transcriptase (Invitrogen, Carlsbad, CA, USA), 25 ng random hexamers and 2.5 μM oligo (dT) 20 primers according to the manufacturer’s recommendations. Real-time PCR was performed in the presence of 1.25 μL of cDNA template, TaqMan® gene expression master mix and commercially available TaqMan® gene expression assays. It was run on ABI Prism® 7900HT Sequence Detection System (Applied Biosystems, Foster City, CA, USA). TaqMan® assays were: CBX5 (Hs01127577_m1), TCF3 (Hs00413032_m1), DOCK10 (Hs00391515_m1), MAN1C1 (Hs00220595_m1), ALDH1A1 (Hs00946916_m1), HSPA8 (Hs03044880_gH), PSMC4 (Hs00197826_m1), HIP2 (Hs00193507_m1), SKP1 (Hs00749532_s1), PGK1 (Hs99999906_m1) and UBC (Hs00824723_m1). Thermal cycler conditions were as follows: 50 °C for 2 min and 95 °C for 10 min, followed by 40 cycles of amplification at 95 °C for 15 s and 60 °C for 1 min. Amplification efficiencies were higher than 90 % for each primer set. Reactions were run in duplicate and a replica was performed. Negative controls, such as non-templates wells as negative reverse transcriptase controls, were assembled to rule out respectively DNA cross contamination of the reagents and genomic DNA in the samples. The amplified products were separated on a 2 % agarose gel and visualized with ethidium bromide staining. To identify the best candidate genes as endogenous controls for normalization, 8 PD and 8 HC age-matched peripheral blood samples were analyzed using the TaqMan® array human endogenous control Cards (Applied Biosystems, Foster City, CA, USA). As the best reference genes, PGK1 and UBC were similarly selected by geNorm and Normfinder applications. The relative gene expression was evaluated by normalization to the geometric mean of the 2 selected endogenous controls and a pool of HC samples was used as calibrator. Statistical analysis relied on Qbase plus software (SPSS Ltd., UK) and graphs were generated with Graphpad Prism 6.0 (Graphpad Software Inc, USA).

### Availability of supporting data

Microarray data sets are available in the Gene Expression Omnibus (GEO; http://www.ncbi.nlm.nih.gov/projects/geo/) with Accession Number GSE72267. Supporting data (Additional files 1, 2, 3, 4, 5, 6, 7, 8, 9, 10, 11, 12, 13 and 14) are included in the article.

## Results

### Study design

Here we aim to identify gene expression patterns in peripheral blood of *de novo* and drug-naïve PD patients by comparing 40 sporadic PD versus 20 HCs (“Discovery set”). To this purpose, patients were enrolled at the early clinical stage of the disease as evaluated by a neurologist. Subjects did not take any centrally acting drugs in the previous 6 months. Table 1 shows the demographic and clinical characteristics of all enrolled subjects. As expected, most patients showed a prevalent asymmetric parkinsonian symptomatology that, in the majority of cases, comprised the classical triad of tremor, bradykinesia, and rigidity. Accordingly, an asymmetric reduction of striatal activity on the ^123^I-FP-CIT SPECT images was observed. The prevalent case consisted of a putaminal alteration contralateral to the clinical most affected side.

Experiments were carried out aiming to optimize the protocol for blood transcriptomics to satisfy the required criteria for biomarker discovery [4]. Special attention was devoted to assess the effects on gene expression profiles of patient’s physiological status at the time of collection and of storage conditions of biological samples (data not shown). After comparing three methods for the synthesis of microarray hybridization targets, the Ovation^TM^ Whole Blood Solution (NuGEN) was chosen for its high sensitivity (three times increase in Present call) and robust reproducibility (Additional file 1). Blood was collected from study subjects into PAXgene Blood RNA tubes (PreAnalytiX, Hombrechtikon, CH) after a fasting period and at the same time of the day to limit circadian-dependent variability. Furthermore, hematological values were determinated to exclude any significant difference among blood cell types that could affect RNA composition. Total RNA was purified using PAXgene^TM^ Blood RNA kit (PreAnalytiX GmbH, Qiagen, Hilden, Germany) and underwent two DNaseI digestion steps. Only high quality RNAs (RNA Integrity Number or RIN ≥ 8) were included in the study as determined with 2100 Bioanalyzer (Agilent Technologies, Palo Alto, CA). Hybridization targets were synthesized with Ovation™ Whole Blood Solution (NuGEN) and hybridized to HG − U133A 2.0 arrays (Affymetrix, Santa Clara, CA), investigating the expression of 18400 transcripts. For details, see Material and methods.

### A gene panel discriminates PD patients from HCs

**Fig. 1:**
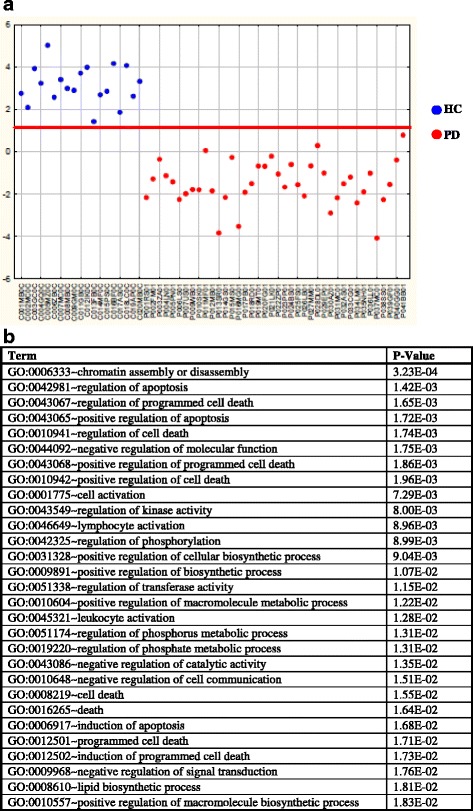
Ranking-PCA applied to the 395 selected variables to compare PD patients and HCs. **a**. Representation of the samples along the first canonical root. The first canonical root (y-axis) is reported for each sample (x-axis). Blue circles correspond to control samples while red circles to pathological samples. The solid line represents the separation between the two classes. The variables are reported in Additional file 3 in the order in which they are included in the model. **b**. Functional annotation analysis of the 395 variables. Over-represented GO annotations with at least 10 genes and *P* < 0.02 (Fisher exact probability) are presented. The complete data of enriched GO annotations are available in Additional file 5

A statistical analysis was carried out to identify a gene panel able to discriminate between patients and controls. To this purpose we took advantage of R-PCA [18] to select the most discriminant variables followed by LDA to obtain the best classification model with a maximum of 6 PCs (Additional file 2). Thus 395 variables were selected (Additional file 3) and a model including five highly significant PCs (*p*-levels < 0.01) (PC_2_, PC_6_, PC_3_, PC_5_, PC_1_) correctly classified all the samples (Fig. 1a). The squared Mahalanobis distance (Additional file 4) of each sample from the centroid of its own and of the other class showed that the classification performance was very robust, the only exception being sample P041BB01 characterized by similar distances from the two classes.

We then analyzed the set of 395 variables, identified by 466 probes, for biological processes significantly enriched in the panel. As shown in Fig. 1b and in Additional file 5, processes such as “regulation of apoptosis”, “lymphocyte activation”, “leukocyte activation” and “lipid biosynthetic process” were found. These expected findings were already previously associated to PD in blood and *post-mortem* brains confirming the experimental and bioinformatic pipeline. Moreover, we originally identified several GO terms associated to epigenetic remodeling including “chromatin assembly or disassembly” (GO:0006333).

### Differential gene expression analyses of blood samples of drug-naïve sporadic PD patients

To identify differentially expressed genes between sporadic PD patients and controls, analysis of gene expression profiles was performed with different bioinformatics algorithms. First, we applied a recently introduced probabilistic model (PUMA) [20] to estimate fold changes and their significance for each probe on the array. No filters were applied to the dataset. By selecting an arbitrary threshold of 1 % probability of including false positives, 306 differentially expressed probes between the two groups were identified corresponding to 282 unique genes (Additional file 6). We also carried out analysis with the parametric test SAM [21] (Additional file 7) and with the non-parametric RP [22] (Additional file 8). These analyses showed respectively 107 and 280 genes common with the ones obtained with PUMA.

Importantly, 54 genes were identified with R-PCA, PUMA and SAM representing a list of candidates for biomarker discovery (Additional file 9).

### Tissue expression and functional analysis of differentially expressed genes

A tissue enrichment analysis was then carried out on the list of differentially expressed genes obtained with PUMA. Surprisingly, 50 % of them were enriched in brain [24] (Table 2a). Furthermore, we compared them to those genes expressed in the DA neurons of the SN, the key site of degeneration in PD. To this purpose, we took advantage of the gene expression profile of mouse DA neurons in the SN that we have recently obtained by coupling transgenic labelling, Laser Capture Microdissection and Affymetrix expression analysis [26–28]. After converting the mouse gene annotation to human annotation, 135 genes were common to the 282 genes identified with the co-expression analysis (Additional file 10). These findings were confirmed for the list of genes obtained with SAM (data not shown) and prove an extensive overlap between genes expressed in mouse DA neurons and those differentially expressed in the blood of PD patients.

**Table 2:** Identification of tissue-enrichment terms and biological processes associated with selected genes dysregulated in blood of PD patients versus controls

Category	Term	Count	Percent	*P*-Value	Benjamini
**a**					
UP TISSUE	Brain	141	50.00	7.4E-3	7.1E-1
UP TISSUE	Testis	70	24.08	1.9E-2	8.0E-1
UP TISSUE	Epithelium	59	20.09	3.2E-2	8.4E-1
UP TISSUE	Lymphoma	4	1.04	6.1E-2	9.3E-1
UP TISSUE	Testicle	3	1.01	7.4E-2	9.2E-1
**b**					
GOTERM_BP_FAT	ncRNA processing	10	3.05	4.2E-3	1.0E0
GOTERM_BP_FAT	regulation of gene expression, epigenetic	6	2.01	1.1E-2	1.0E0
GOTERM_BP_FAT	lymphocyte activation	10	3.05	1.3E-2	1.0E0
GOTERM_BP_FAT	lipid homeostasis	5	1.08	1.5E-2	1.0E0
GOTERM_BP_FAT	chromatin assembly or disassembly	7	2.05	1.7E-2	9.9E-1
GOTERM_BP_FAT	midbrain development	3	1.01	2.0E-2	9.9E-1
GOTERM_BP_FAT	chemical homeostasis	17	6.00	2.9E-2	1.0E0
GOTERM_BP_FAT	negative regulation of protein kinase cascade	4	1.04	3.0E-2	1.0E0
GOTERM_BP_FAT	ncRNA metabolic process	9	3.02	3.5E-2	1.0E0
GOTERM_BP_FAT	regulation of action potential	5	1.08	3.8E-2	1.0E0
GOTERM_BP_FAT	leukocyte activation	10	3.05	3.9E-2	1.0E0
GOTERM_BP_FAT	cell activation	11	3.09	4.2E-2	1.0E0

**a**)Tissue enrichment analysis. Count: number of genes involved in the term; %: percentage of involved genes/total genes; *P*-Value: modified fisher exact *P*-value, EASE Score; Benjamini: adjusted P-value using Benjamini-Hochberg procedure. **b**)Biological processes identified by GO annotations (DAVID)

We then took advantage of Gene Ontology and GSEA to identify enriched biological pathways (Additional 11). As expected from previous studies both in PD blood and *post-mortem* brains, the most significant terms include “lymphocyte activation”, “lipid homeostasis”, “midbrain development” and “leukocyte activation”. Importantly, we also identified for the first time a strong enrichment in epigenetic-related Gene Ontology terms such “regulation of gene expression, epigenetic” and “chromatin assembly or disassembly” (Table 2b). These processes were also enriched in the list of differentially expressed genes obtained with SAM (Additional file 7). To validate the involvement of transcripts associated to chromatin remodeling and methylation-dependent processes, blood samples from the “Discovery set” were tested with RT-qPCR assays for the differentially regulated CBX5, HELLS and MECP2 mRNAs as well as for ASFA1, DNMT3A and PRMT1 as part of the very same GO gene list (Additional file 11). As shown in Additional file 12, their differential expression was confirmed.

### Validation of selected differentially expressed genes

We carried out RT-qPCR on additional un-profiled samples to independently assess expression changes for a selected group of genes obtained from the array data. Blood samples were collected from 24 sex-, age- and ethnicity-matched subjects: 12 sporadic PD (68.8 years ± 5.2 SD) and 12 HC (68.0 years ± 1.5 SD) (“Validation set”). Enrolment inclusion criteria and procedures were as those defined for the “Discovery set”. Clinical and demographic characteristics of subjects are reported in Table 1.

**Fig. 2:**
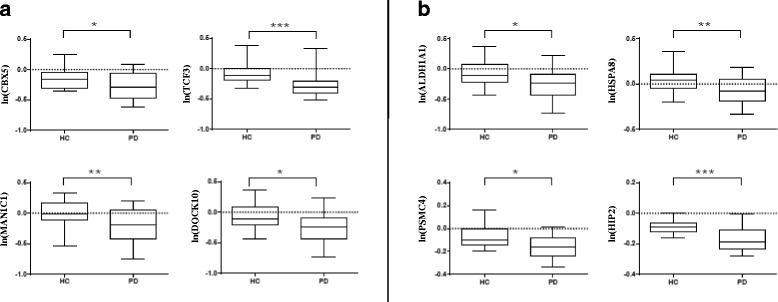
RT-qPCR validation experiments of selected transcripts. The box plots show the natural logarithms of the relative gene expression levels (calculated by dividing the RT-qPCR values by the geometric mean of the HKs PGK1 and UBC raw quantities) for the individual genes (**a** and **b**) in blood samples of 12 *de novo* PD patients and 12 age- and sex-matched HCs. The statistical significance was calculated by t-test (*p < 0.05, **p < 0.01, ***p < 0.001)

Genes for validation were selected from the candidates list of biomarkers commonly obtained with R-PCA, PUMA and SAM analysis (Additional file 9). Four transcripts were chosen among the ones with the most significant *P*-values (*p* < 0.0003) and tested with RT-qPCR. Data were normalized to the geometric mean of PGK and UBC, the most reliable reference genes. These were identified using TaqMan® array human endogenous control cards (Applied Biosystems, Foster City, CA, USA) as reported in Materials and methods section and in Additional file 13. As shown in Fig. 2a, statistically significant results from RT-qPCR analysis were achieved for Chromobox homolog 5 (CBX5) (*P* = 2.88E-02), Transcription factor 3 (TCF3) (*P* = 5.12E-04), Dedicator of cytokinesis 10 (DOCK10) (*P* = 1.52E-02) and Mannosidase, alpha class 1C (MAN1C1) (*P* = 1.15E-03). Overall, these RT-qPCR data validate expression changes identified through array analysis with a different technology and on an independent set of samples.

To correlate these findings with genes previously identified as differentially expressed in PD *post-mortem* brains and blood [29, 30], we have found a significant difference of expression in the very same “Validation set” for Aldehyde Dehydrogenase 1 Family, member A (ALDH1A1) (*P* = 1.51E-02), Proteasome (prosome, macropain) 26 S subunit PSMC4 (*P* = 1.80E-03) and Heat shock 70 kDa protein 8 (HSPA8) (*P* = 8.20E-03) but not for Huntingtin interacting protein 2/ubiquitin-conjugating enzyme E2K (HIP2/UBE2K) (*P* = 3.00E-04) (Fig. 2b).

### Drug network analysis of PD-associated genes

**Fig. 3:**
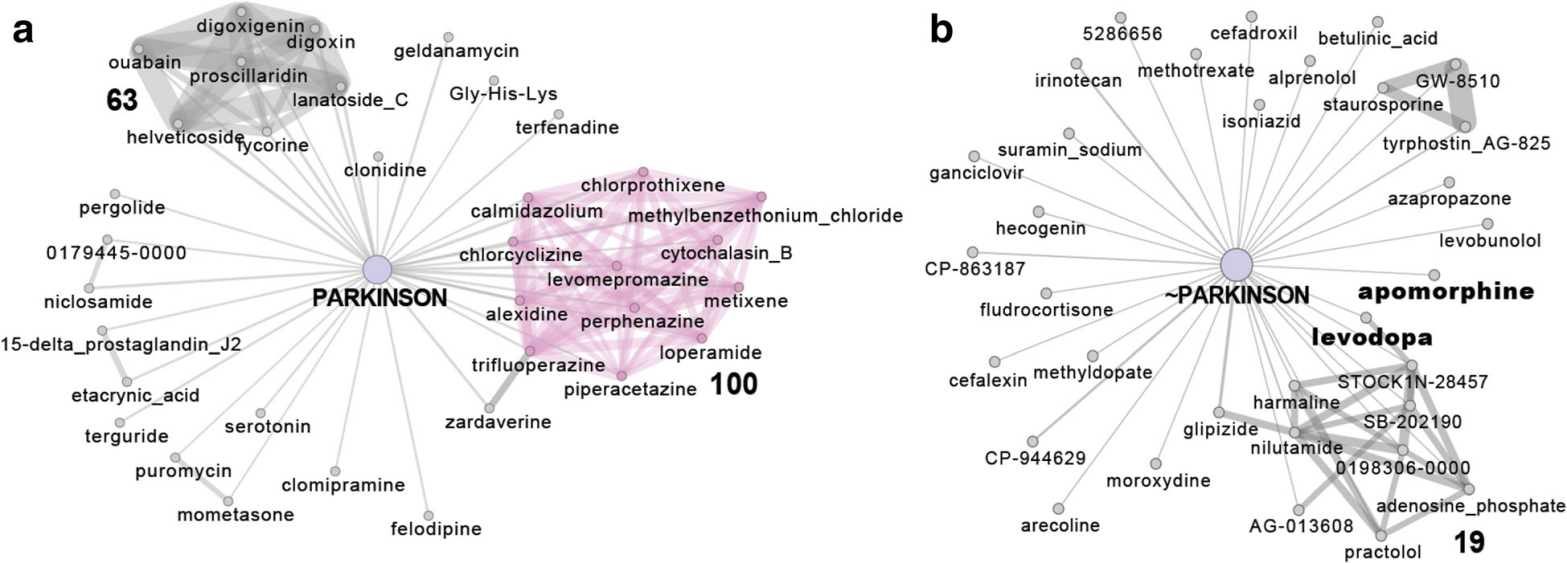
Drug network analysis. Sub-networks connected to genome-wide ranked lists of genes sorted according to their differential expression in PD: decreasing order in (**a**) and increasing order in (**b**), once they are integrated in the drug network as described in Iorio et al. [31]. For clarity we included only the first 35 most similar (resp. “anti-similar”) to PD drugs. Edge thickness is inversely proportional to the distance between the drugs and the conditions. Several antipsychotic drugs (community n. 100 in **a**) elicit a transcriptional response similar to PD while compounds used for PD treatment elicit an “anti-similar” to PD response (bold in **b**). The complete list is available in Additional file 14

In the search for potential new PD treatments we investigated whether FDA-approved drugs could elicit a transcriptional profile similar or opposite to the one observed in peripheral blood of PD patients. This approach is based on the observation that a large portion of differentially expressed genes in PD blood is expressed in mesencephalic DA neurons and that altered GO biological terms are common in PD blood and *post-mortem* brains. To this purpose we took advantage of a new approach to identify drug mode of action from gene expression profiles [31]. Specifically, drugs are connected in a network if they elicit a similar transcriptional response according to a new similarity measure based on a modification of GSEA [25]. The drug network consists of 1309 compounds that can be subdivided in 106 communities of drugs, i.e. groups of drugs very similar to each other with a similar mode of action but very different from other drugs in the network. To investigate which drugs trigger the most similar response to the changes found in PD patients, genes were ranked according to their differential expression in PD versus control and the network was queried. The drugs sorted according to their similarity to PD are shown in the Additional file 14. Interestingly, as reported in Fig. 3a, several antipsychotic drugs elicited a transcriptional profile similar to PD (Community 100, *P* = 3.83x10-6 considering the top-ranked 35 similar to PD drugs and *P* = 6.76x10-9 considering the top-ranked 100 ones). On the other hand, examining the drugs which elicit an “anti-similar” transcriptional response (i.e. which up-regulate genes found down-regulated in PD and vice-versa), it is noteworthy that apomorphine and levodopa occupy top-ranked positions and are currently used for treating PD (Fig. 3b and Additional file 14).

## Discussion

This work is the largest study to date of the peripheral whole blood transcriptome of drug-naïve sporadic PD patients at the time of their first diagnosis. A gene panel discriminates PD patients from healthy controls. While we confirmed alterations previously found in PD blood and *post-mortem* brains such as neuronal apoptosis, lymphocyte activation and mitochondrial dysfunction, we have also unveiled changes enriched in biological terms related to epigenetic modifications including chromatin remodeling and methylation.

A correct diagnosis at the early stages of the disease remains an unresolved and crucial issue in PD. This is important to choose the best medical treatment from disease onset and to apply the best criteria for patient selection and enrollment in clinical trials. The potential of blood as a surrogate tissue in PD is under intense scrutiny and blood transcriptomics is expected to facilitate the identification of biomarkers for early diagnosis and drug discovery [32].

A short but significant list of recent microarray-based studies has used human blood as RNA source to look for differentially expressed genes in sporadic and genetic PD patients [5–11]. In the pioneering work by Scherzer et al. [5], 22 genes were identified with microarray profiling of whole blood of 50 PD patients, among which 9 *de novo* subjects. A risk marker given by 8 genes (VDR, HIP2, CLTB, FPRL2, CA12, CEACAM4, ACRV1, and UTX) predicted PD and was not biased by dopamine replacement therapy. The analysis of a genetically homogenous population of 88 Ashkenazi patients [6], including 20 *de novo*, evidenced for the first time the decreased expression of B cells-related genes in PD. Karlssonn et al. [8] analyzed samples from 79 PD subjects at different stages of the disease, including 23 *de novo* patients and relative controls, proposing a classifier predicting sporadic and *de novo* PD. LRPPRC, BCL2 and SRSF8 were shared with the 22 genes list as in Scherzer et al. [5]. Furthermore, it presented HSPA8 and UBE2K/HIP2 in common with Molochnikov et al. [30]. Potashkin et al. [7] took advantage of splice variant-specific microarrays to identify a biosignature composed of 13 mRNAs (c5orf4, wls, macf1, prg3, eftud2, pkm2, slc14a1-s, slc14a1-l, mpp1, copz1, znf160, map4k1 and znf134) whose expression is altered in peripheral blood of early-stage PD patients. Recently, they identified two novel longitudinally markers (HNF4 and PTB1) by means of network-based and transcriptomic meta-analyses [33]. Interestingly, gene expression analysis of peripheral blood mononuclear cells from 20 sporadic PD patients and 9 individuals, heterozygous for the LRRK2 G2019S mutation, showed deregulation of the immune system, endocytosis and eukaryotic initiation factor 2 signaling [11].

Although there are a number of promising gene signatures, blood transcriptomics have not yet delivered the expected results for biomarker discovery in PD. One of the major concerns is the scarce overlap among candidate genes lists of these studies. Variances in the procedures for collection, processing and analysis of samples may strongly limit the reproducibility of gene expression data. Importantly, these differences may also be explained in biological terms. First, genetic variations in human populations may lead to diversity in transcriptional changes in disease. Furthermore, the majority of these works analyzed peripheral blood samples from sporadic PD patients at different stages of the disease and under pharmacotherapy raising the questions of whether changes are related to the disease stage, therapy or both. Finally, it is now clear that PD is a systemic and a highly heterogeneous disease, as classified according to distinct clinical subtypes [34].

On the other hand, blood transcriptomics studies identify a common repertory of enriched GO biological terms as altered in PD. These include “neuronal apoptosis”, “mitochondrial dysfunction”, “leukocyte activation” and “deregulation of the immune system”.

To overcome part of the limitations of previous analyses, our study design takes advantage of 1) a carefully selected drug-naïve and *de novo* ethnically-defined PD population size, 2) standardized, simple and well-defined technologies and methods whose protocols and procedures are generally recognized as robust and reproducible. Our purpose was to establish an optimized pipeline to collect biological samples for blood transcriptomics to minimize confounding signals such as artifacts of sample preparation and processing while maximizing reproducibility and sensitivity. Most importantly, the use of the NuGEN method for target preparation results in higher sensitivity (three times increase in Present calls) leading to a wider range of intensities and a smaller impact of technical variability.

By performing data analyses, we prove that gene expression profiling of peripheral blood discriminates patients from HCs. Furthermore, we identified a list of genes (Additional file 9) that were differentially expressed in PD patients at the onset of motor symptoms and before initiating any pharmacological treatment. Selected transcripts were validated in an independent cohort of patients and HCs (“Validation set”) with a different technology (RT-qPCR) strengthening our findings.

A very limited overlap was found in the identities of single genes between our study and those previously published. This is not surprising and probably due to differences in the cohort of patients and in technical settings.

The level of expression changes between PD patients and HC was in the range of 20 to 50 %. While these values are similar to the ones identified in other blood transcriptomics studies, patients were at an early stage of the disease and differential expression may increase with disease progression. Furthermore, while differential expression may occur only in selected cells’ populations, genes may be expressed in the majority of blood cell types decreasing fold changes measured in whole blood.

Our analyses confirmed the main biological GO terms found altered in previous blood transcriptomics of PD patients. Together with “neuronal apoptosis” and “mitochondrial dysfunction”, differentially expressed pathways include “lymphocyte activation” and “leukocyte activation”. Among the single validated transcripts, MAN1C1, mannosidase, alpha, class 1C, member 1 is a protein partner of MAN1A2 previously identified by Scherzer et al. [5]. These two key α1,2-mannosidases catalyze the earliest steps of mannose removal required for the conversion of high mannose to hybrid and subsequently complex *N*-glycans. Current hypothesis suggests a key role for core *N*-glycan structures (i.e. mannose) in providing signals to the innate immunity system for recognizing cells during inflammation [35]. Transcription factor 3 (TCF3), also known as E2A, plays an important role in the development and differentiation of B and T lymphocytes [36]. It mainly functions as a transcriptional repressor [37] being counteracted at multiple levels by Wnt signaling [38]. This pathway is crucial in normal function and survival of midbrain DA neurons [39] and its alteration has been already reported in gene expression studies of peripheral blood of PD patients [7, 11]. DOCK10 (Dedicator of cytokinesis 10) is a gene that encodes a member of the zizimin subfamily belonging to the Dock protein family, comprising atypical Rho guanine nucleotide exchange factors for Rac and/or Cdc42 GTPases [40]. Dock10 may represent a point of convergence for IL-4 signaling and small Rho GTPase function in B cells [41] and this might be important for the well-known dysregulation of IL-4 signaling in peripheral blood of sporadic PD [11].

Importantly, we make the original observation that crucial elements of chromatin remodeling and methyltransferase machineries are major targets of PD-associated molecular events in living patients suggesting a role of epigenetic regulation in neurodegeneration [42]. Recent data support epigenetic modulation in neurodegenerative diseases such as Alzheimer’s, Huntington’s and Amyotrophic Lateral Sclerosis. A direct relationship between epigenetics and PD has not been systematically assessed although sparse evidences are available. Dopamine depletion in PD is associated with a reduction in histone H3K4me3, whereas chronic levodopa therapy leads to deacetylation of histones H4K5, K8, K12, and K16. Treatment of animals with MPTP (1-methyl-4-phenyl-1,2,3,6- tetrahydropyridine), widely used as a PD model, induces H3 acetylation, which is reduced after treatment with levodopa [43]. Interestingly, methylation of *SNCA* intron 1 was found reduced in DNA from sporadic PD patients’ SN, putamen, and cortex, while its expression in a PD patient heterozygous for the A53T mutation was found to be monoallelic due to epigenetic silencing [44–46]. In fact, hypomethylation of SNCA and LRRK2 in leukocytes of peripheral blood has been suggested as a potential noninvasive biomarker for PD early diagnosis [47]. Most recently, α-synuclein was found to trigger DNMT1 aberrant cytoplasmic localization in PD *post-mortem* brains and animal models leading to a global DNA hypomethylation. Furthermore, DNMT1 protein itself was reduced to 50 % in *post-mortem* PD brains [48].

In this context an intriguing chain of events has been proposed linking mitochondrial dysfunction to epigenetic changes in PD [49–51]. The age-dependent down-regulation of metallothioneins may render DA neurons susceptible to oxidative stress and Charnoly body formation, an early and universal mitochondrial biomarker of cell injury, apoptosis and progressive neurodegeneration. In turn, the formation of 8-hydroxy, 2-deoxyguanosine, a PD biomarker in urine, can affect the epigenetic status of nuclear DNA.

Currently, several epigenetic-based drugs are investigated as potential treatment strategies for PD, including HDAC and DNMT inhibitors [52–55]. Further work is needed in evaluating these promising therapeutics.

Chromobox homolog 5 (CBX5), also named HP1a, is the most differentially expressed gene in our analysis. It belongs to a class of multifunctional chromatin-associated adapter proteins present in constitutive heterochromatin. There it plays an essential role in establishing and maintaining heterochromatin-mediated gene silencing [56]. In addition to self-association, HP1-interacting partners include the DNA methyltransferase DNMT3A, which we have found decreased in PD patients. DNMT3A is required for *de novo* DNA methylation [57], and its activity has been related to several functions in the nervous system including neuronal differentiation, synaptic plasticity and memory formation. Microarray data also detect changes in the expression of other genes involved in chromatin remodeling and epigenetic regulation such as MECP2, an essential epigenetic regulator in human brain development that has been associated to activity-dependent synaptic maturation [58], and ASF1A, a histone chaperone that participates in nucleosomes disassemblies and interacts with histone-acetylation-recognizing bromodomains [59]. Protein arginine methyltransferase (*Prmt1*), Thymocyte selection-associated high mobility group box (*Tox*), Enhancer of zeste homolog 1 (*Ezh1*), and Sin3A-associated protein (*SAP30*) were also dysregulated.

Changes in peripheral blood seem to reflect molecular events in the brain. Selected genes and GO terms, identified as dysregulated in PD *post-mortem* brains, were previously confirmed as altered in the peripheral blood of living patients. In this context, Grunblatt et al. [29] carried out RT-qPCR in more than 100 medicated and 11 *de novo* PD patients to study 12 transcripts previously proved to be differentially expressed in PD *post-mortem* brains. Four of them were proposed as biomarkers for PD with a specificity of more than 80 %. Recently, Molochnikov et al. [30] presented a highly similar five-gene set (SKP1A, HIP2, ALDH1A1, PSMC4 and HSPA8) that differentiated early PD from HCs.

Here we show that expression of PD-associated genes in blood is enriched in brain tissue. Furthermore, about 50 % of these genes are also expressed in DA neurons of the SN, the key site of neurodegeneration in PD. As expected, the enriched GO terms of this gene expression study present a significant overlap with those unveiled in gene expression profiling of PD *post-mortem* brains. Consequently, we specifically investigated the expression of selected genes previously shown as differentially expressed in both PD *post-mortem* brains and blood [29, 30]. Considering that we could not observe gene expression changes for those genes since no Affymetrix ID probes were present on the chips used in this study, we took advantage of RT-qPCR assays to test their expression in the “Validation set” of samples. We thus confirmed a significant difference of expression for ALDH1A1, a detoxification enzyme that participates in the metabolism of catecholamines and plays a key role in the protection of the nigrostriatal DA neurons [60], for PSMC4, involved in the ubiquitin proteasome degradation pathway and for HSPA8 (Fig. 2b). Their differential expression further corroborates that events associated with degeneration in PD *post-mortem* brain can also be detected in peripheral blood of living patients.

These observations are substantially strengthened by our drug network analysis. Among the 106 communities that grouped 1309 compounds for their gene expression patterns, our study revealed that the “antipsychotic” drug community was significantly mimicking PD gene expression phenotypes. This community of drugs, acting at the level of DA neurotransmission, highlights that we are detecting changes in gene patterns relevant for CNS function in PD. In addition, drugs as apomorphine and levodopa, two molecules currently used in PD clinical treatment, are selected in the community of drugs that elicit an “antisimilar” transcriptional response.

## Conclusions

To our knowledge, this is the largest study to date using whole peripheral blood to investigate early gene expression changes in drug-naïve and *de novo* sporadic PD patients. We are conscious that replication is needed in a larger well-characterized prospective study to confirm and define the clinical use of this set of transcripts.

The identification of altered biological pathways as early events in living sporadic PD subjects [61] may direct future investigation in the search of validated drug targets for therapeutic intervention. In this context, it will be interesting to study the role of epigenomic changes in PD and the use of epigenetic modifiers as PD candidate drugs.

## Additional files

Additional file 1:
**Venn diagram of probe sets scored as present calls following three different amplification methods.** Blood collected from a single healthy donor was divided in 3 groups of 2 samples each, and each group was processed for microarray target preparation according to 1) Affymetrix one − cycle cDNA synthesis/Affymetrix IVT (Affymetrix), 2) Illumina probe synthesis protocol (Ambion), 3) Ovation™ Whole Blood Solution (NuGEN). Total RNA input amounts were based on manufacturer’s recommendations for each method. Hybridizations of targets were performed to HG − U133A 2.0 arrays (Affymetrix, Santa Clara, CA). Cell intensity values and probe detection calls were computed using the AffymetrixGeneChip Operating Software (GCOS). This Venn diagram shows the overlap of Affymetrix probe sets with detection call “Present” following the three amplification methods (Affymetrix, Illumina and NuGEN). Data are relative to one of the two replicates and are good representatives of both replicates. 3553 present calls were detected exclusively using the NuGEN (Ovation TM Whole Blood Solution) method in a reproducible fashion. Therefore, despite starting from a much lower RNA amount, the NuGEN protocol detected a significant number of transcripts that were not identified with any of the other methods tested in this study (~29 % and 37 % more, respectively, than with the Illumina and Affymetrix protocols). (PDF 3122 kb) Additional file 2:
**Score plots of the first 6 PCs calculated on the dataset constituted by the 395 variables selected by Ranking-PCA.** Control samples are represented as filled circles while pathological samples as void circles. Of the original 60 samples, one (a control sample) did not pass the microarray hybridization quality controls and was excluded from further analyses. All results of bioinformatics analyses shown in this paper refer to this set of 59 samples. (PDF 3122 kb) Additional file 3:
**List of the 395 variables selected by Ranking-PCA to discriminate between PD patients and controls.** Order of variables, Affymetrix Probe Set IDs, gene symbols and names are indicated. (PDF 3122 kb) Additional file 4:
**Square of the Mahalanobis distance calculated for each sample from both control and pathological class models.** (PDF 3122 kb) Additional file 5:
**Main processes (GOTERM_BP_FAT as classified by DAVID Gene Ontology) altered in whole peripheral blood cells in the 395 selected variables comparing PD patients and controls.** GO annotations with at least 3 genes and *P* < 0.05 (Fisher exact probability) are presented. Count: number of genes involved in the term; %: percentage of involved genes/total genes; *P*-Value: modified fisher exact *P*-value, EASE Score; Benjamini: adjusted *P*-value using Benjamini-Hochberg procedure. (PDF 3122 kb) Additional file 6:
**List of differentially expressed genes between PD patients and controls based on PUMA.** Ratios are calculated as differences in gene expression between samples of PD patients and controls, with the top up-regulated genes first. (PDF 3122 kb) Additional file 7:
**List of differentially expressed genes between PD patients and controls based on Significance Analysis of Microarrays (SAM).** Significance Analysis of Microarrays was performed on normalized and filtered microarray data as described in the main text. The SAM test was run using 1000 permutations and a False Discovery Rate (FDR) of 10 %. Ratios are calculated as differences in gene expression between samples of PD patients and controls, with the top up-regulated genes first. (PDF 3122 kb) Additional file 8:
**List of differentially expressed genes between PD patients and controls based on Rank Product analysis (RP).** Rank Product analysis was performed on normalized and filtered microarray data as described in the main text. The RP test was run using 100 permutations and an FDR of 0.005 % and looking for up- and down-regulated genes separately. Low RP-Values indicate high significance of the results. Genes are shown with the most significant first. (PDF 3122 kb) Additional file 9:
**List of common differentially expressed genes between PD patients and controls based on SAM and PUMA analyses and the 395 selected variables of the Ranking-PCA.** Genes are ranked according to the P- values based on PUMA analysis. Order of the 395 variables, Affymetrix Probe Set IDs, gene symbols and names are indicated. Names shown in bold indicate transcripts validated by RT-qPCR (Fig. 2a). (PDF 3122 kb) Additional file 10:
**List of common genes between A9 DA neurons of the SN in the mouse and differentially expressed transcripts in the blood of PD patients according to SAM (a) and PUMA (b).** (PDF 3122 kb) Additional file 11:
**Statistically significant gene sets from GSEA analyses.** GSEA was run on the normalized, unfiltered microarray dataset as suggested in the tools implementation (http://www.broadinstitute.org/gsea/ version 2.06), and using the c5 - GO gene sets collection of the Molecular Signatures Database (MSigDB) (http://www.broadinstitute.org/gsea/msigdb/). The test was performed separately on each of the c5 sub-collections (biological process, molecular function and cellular component), running 1000 permutations and excluding gene sets with fewer than 5 genes or more than 150 (the latter, to retain granularity). Names (Gene Symbol) shown in bold indicate transcripts measured by RT-qPCR (Additional file 12). (PDF 3122 kb) Additional file 12:
**Genes involved in chromatin remodeling and methylation are targeted in PD.** Selected samples (12 PD and 12 HC blood samples) previously processed for gene expression profiling were also tested in RT-qPCR assays for several targets (CBX5, HELLS, MECP2, ASF1A, DNMT3A and PRMT1) that were identified by data analyses. Retrotranscription was performed using 1 μg of RNA and the iSCRIPT™ cDNA Synthesis Kit (Bio-Rad) according to the manufacturer’s protocol. Real Time qPCR was executed using SYBER-Green PCR Master Mix (Applied Biosystem) and an iCycler IQ Real Time PCR System (Bio-Rad). Sequences of gene specific primers are reported in the table below. Expression of the gene of interest was normalized to *β-actin*. The relative expression of each sample was calculated by the formula 2 exp^-ΔΔCt^ (User Bulletin 2 of the ABI Prism 7700 Sequence Detection System). The amplified products were separated on a 2 % agarose gel and visualized with ethidium bromide staining. (PDF 3122 kb) Additional file 13:
**Selection of reliable reference genes for peripheral blood gene expression analyses. TaqMan® array human endogenous control cards (Applied Biosystems, Foster City, CA, USA) are 384-well microfluidic cards containing 16 human TaqMan Gene Expression Assays.** They were used to evaluate the endogenous controls specific for peripheral blood that exhibit minimal differential expression. Peripheral blood samples from 8 PD and 8 HC gender- and age-matched subjects were processed following the manufacturer’s instructions (Applied Biosystems, Foster City, CA, USA). The expression stability was determined and compared by two commonly used algorithms (geNorm and NormFinder). By comparing the output of these two methods and by accepting gene expression levels of qPCR at Ct values ≤ 29, we obtained a list of the most stable reference genes in human peripheral blood. See the following Figures reporting output files of the analyses. As the best reference genes, PGK1, UBC and GAPDH were selected according to the following practical considerations: PGK1 is the best reference gene according to geNorm and NormFinder analyses; UBC presents similar stability strength to PGK1 and a different threshold Ct value; GAPDH is one of the most widely used reference genes in peripheral blood expression studies. The gene expression analyses of the first experimental data sets (data not shown) reveled that GAPDH had a higher variability compared to PGK1 and UBC; therefore, we decided to evaluate the relative gene expression by normalizing the data to the geometric mean of PGK1 and UBC. (PDF 3122 kb) Additional file 14:
**List of similar- and anti-similar-to-PD compounds from drug network analysis.** Community identifiers, drugs and community enrichment p-values resulting from the drug network analysis of genome-wide ranked lists of genes sorted according to their differential expression in PD. Drugs are sorted according to their similarity to PD (**a**) and according to their “anti-similarity” (**b**) as explained in the main manuscript. (PDF 3121 kb)

## Abbreviations

PD: Parkinson’s disease
CNS: central nervous system
SN: *Substantia Nigra*
DA: dopaminergic
*de novo*: early stage of the disease
drug-naïve: no specific pharmacological treatment
HC: healthy control
SD: Standard Deviation
MMSE: Mini Mental State Examination
SPECT: Single Photon Emission Computed Tomography
PCA: Principal Component Analysis
PLS-DA: Partial Least Square Discriminant Analysis
R-PCA: Ranking-Principal Component Analysis
LDA: Linear Discriminant Analysis
NER%: non-error rate
RMA: Robust Multi-Array Average
MeV: Multiexperiment Viewer
SAM: Significance Analysis of Microarrays
RP: Rank Product
PPLR: probability of positive log ratio
FDR: False Discovery Rate
GO: Gene Ontology
GSEA: Gene Set Enrichment Analysis
RT-qPCR: Quantitative real-time PCR
PCs: Principal Components
P: *P*-value
