# Supplementary material for: Blood transcriptomics of drug-naïve sporadic Parkinson’s disease patients
Source: BMC Genomics. 2015 Oct 28;16:876. doi: 10.1186/s12864-015-2058-3 (PMC4625854; doi:10.1186/s12864-015-2058-3)

Additional file 1. Venn diagram of probe sets scored as present calls following 3 different amplification methods. Blood collected from a single healthy donor was divided in 3 groups of 2 samples each, and each group was processed for microarray target preparation according to 1) Affymetrix one−cycle cDNA synthesis/Affymetrix IVT (Affymetrix), 2) Illumina probe synthesis protocol (Ambion), 3) Ovation^{TM} Whole Blood Solution (NuGEN). Total RNA input amounts were based on manufacturer's recommendations for each method. Hybridizations of targets were performed to HG−U133A 2.0 arrays (Affymetrix, Santa Clara, CA). Cell intensity values and probe detection calls were computed using the Affymetrix GeneChip Operating Software (GCOS). This Venn diagram shows the overlap of Affymetrix probe sets with detection call “Present” following the three amplification methods (Affymetrix, Illumina and NuGEN). Data are relative to one of the two replicates and are good representatives of both replicates. 3553 present calls were detected exclusively using the NuGEN (Ovation TM Whole Blood Solution) method in a reproducible fashion. Therefore, despite starting from a much lower RNA amount, the NuGEN protocol detected a significant number of transcripts that were not identified with any of the other methods tested in this study (~29% and 37% more, respectively, than with the Illumina and Affymetrix protocols).

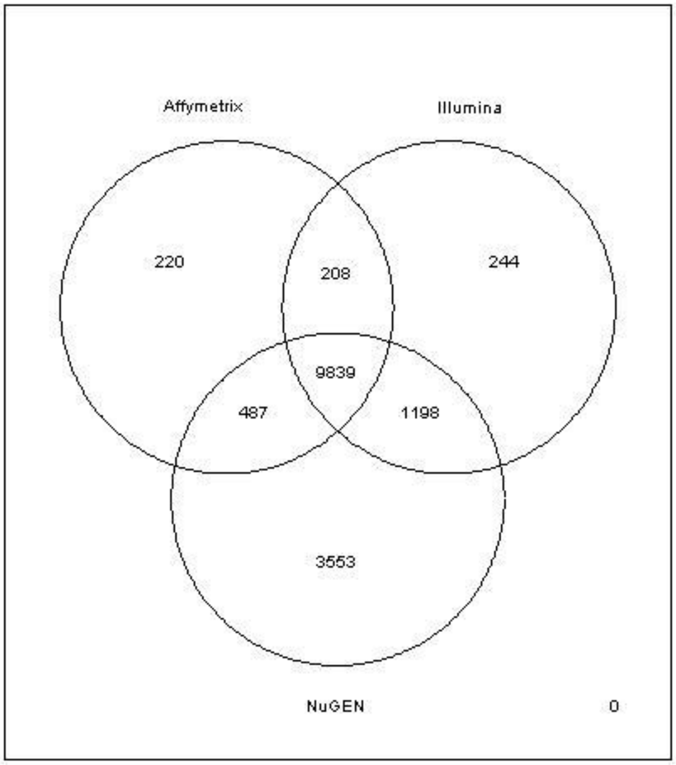

Supplement: Additional file 1: — Venn diagram of probe sets scored as present calls following three different amplification methods. Blood collected from a single healthy donor was divided in 3 groups of 2 samples each, and each group was processed for microarray target preparation according to 1) Affymetrix one − cycle cDNA synthesis/Affymetrix IVT (Affymetrix), 2) Illumina probe synthesis protocol (Ambion), 3) Ovation™ Whole Blood Solution (NuGEN). Total RNA input amounts were based on manufacturer’s recommendations for each method. Hybridizations of targets were performed to HG − U133A 2.0 arrays (Affymetrix, Santa Clara, CA). Cell intensity values and probe detection calls were computed using the AffymetrixGeneChip Operating Software (GCOS). This Venn diagram shows the overlap of Affymetrix probe sets with detection call “Present” following the three amplification methods (Affymetrix, Illumina and NuGEN). Data are relative to one of the two replicates and are good representatives of both replicates. 3553 present calls were detected exclusively using the NuGEN (Ovation TM Whole Blood Solution) method in a reproducible fashion. Therefore, despite starting from a much lower RNA amount, the NuGEN protocol detected a significant number of transcripts that were not identified with any of the other methods tested in this study (~29 % and 37 % more, respectively, than with the Illumina and Affymetrix protocols). (PDF 3122 kb) [file 12864_2015_2058_MOESM1_ESM.pdf]
