# Supplementary material for: Blood transcriptomics of drug-naïve sporadic Parkinson’s disease patients
Source: BMC Genomics. 2015 Oct 28;16:876. doi: 10.1186/s12864-015-2058-3 (PMC4625854; doi:10.1186/s12864-015-2058-3)

Additional file 2. Score plots of the first 6 PCs calculated on the dataset constituted by the 395 variables selected by Ranking-PCA. Control samples are represented as filled circles while pathological samples as void circles. Of the original 60 samples, one (a control samples) did not pass the microarray hybridization quality controls and was excluded from further analyses. All results of bioinformatics analyses shown in this paper refer to this set of 59 samples.

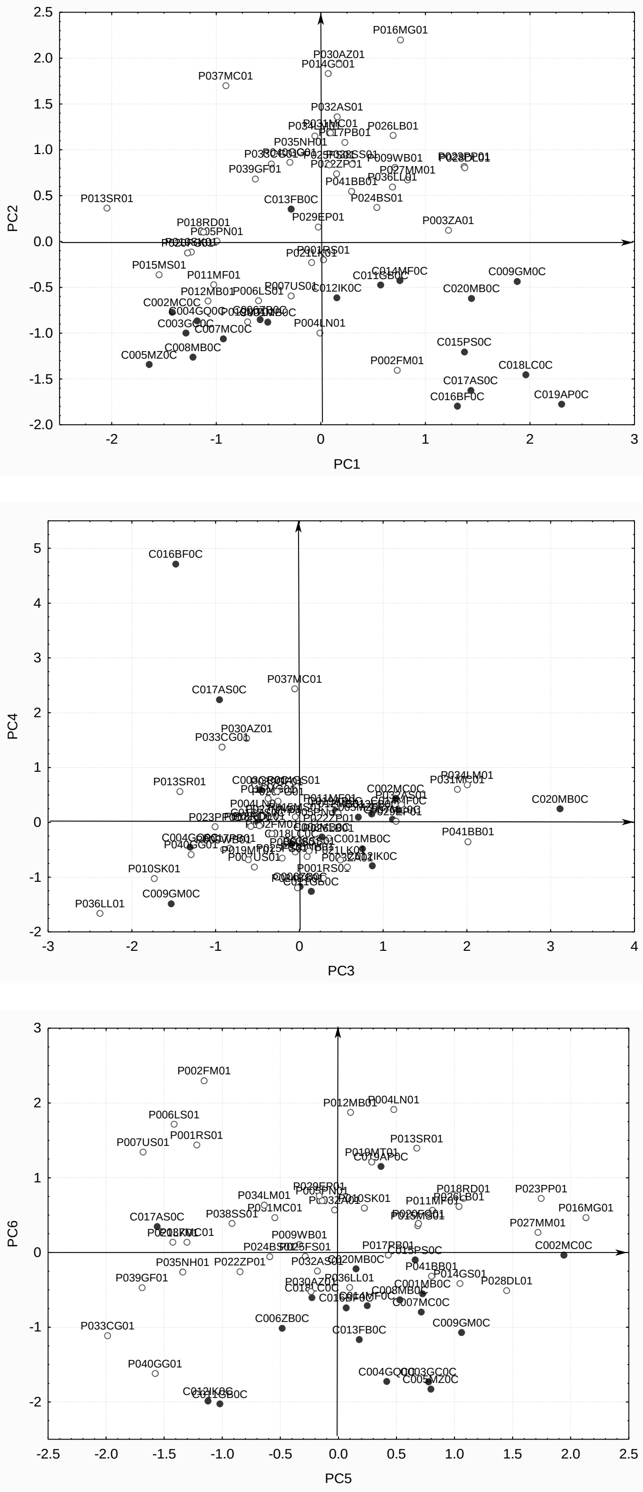

Supplement: Additional file 2: — Score plots of the first 6 PCs calculated on the dataset constituted by the 395 variables selected by Ranking-PCA. Control samples are represented as filled circles while pathological samples as void circles. Of the original 60 samples, one (a control sample) did not pass the microarray hybridization quality controls and was excluded from further analyses. All results of bioinformatics analyses shown in this paper refer to this set of 59 samples. (PDF 3122 kb) [file 12864_2015_2058_MOESM2_ESM.pdf]
