# Supplementary material for: Blood transcriptomics of drug-naïve sporadic Parkinson’s disease patients
Source: BMC Genomics. 2015 Oct 28;16:876. doi: 10.1186/s12864-015-2058-3 (PMC4625854; doi:10.1186/s12864-015-2058-3)
Supplement: Additional file 3: — List of the 395 variables selected by Ranking-PCA to discriminate between PD patients and controls. Order of variables, Affymetrix Probe Set IDs, gene symbols and names are indicated. (PDF 3122 kb) [file 12864_2015_2058_MOESM3_ESM.pdf]

Additional file 3. List of the 395 variables selected by Ranking-PCA to discriminate between PD patients and controls. Order of variables, Affymetrix Probe Set IDs, gene symbols and names are indicated.

Order	Affymetrix probe ID	Gene symbol	Gene name
1	209715_at	CBX5	chromobox homolog 5 (HP1 alpha homolog, Drosophila)
2	215750_at	KIAA1659	KIAA1659 protein
3	207339_s_at	LTB	lymphotoxin beta (TNF superfamily, member 3)
4	208763_s_at	TSC22D3	TSC22 domain family, member 3; GRAM domain containing 4
4	208763_s_at	GRAMD4	TSC22 domain family, member 3; GRAM domain containing 4
5	200921_s_at	btg1	B-cell translocation gene 1, anti-proliferative
6	214739_at	LRCH3	leucine-rich repeats and calponin homology (CH) domain containing 3
7	209153_s_at	tcf3	transcription factor 3 (E2A immunoglobulin enhancer binding factors E12/E47)
8	208893_s_at	dusp6	dual specificity phosphatase 6
9	221592_at	TBC1D8	TBC1 domain family, member 8 (with GRAM domain)
10	217492_s_at	PTENP1	phosphatase and tensin homolog; phosphatase and tensin homolog pseudogene 1
10	217492_s_at	pten	phosphatase and tensin homolog; phosphatase and tensin homolog pseudogene 1
11	217807_s_at	GLTSCR2	glioma tumor suppressor candidate region gene 2; glioma tumor suppressor candidate region gene 2 pseudogene
11	217807_s_at	LOC440311	glioma tumor suppressor candidate region gene 2; glioma tumor suppressor candidate region gene 2 pseudogene
12	210847_x_at	TNFRSF25	tumor necrosis factor receptor superfamily, member 25
13	213963_s_at	SAP30	Sin3A-associated protein, 30kDa
14	221649_s_at	ppan	peter pan homolog (Drosophila)
15	203232_s_at	ATXN1	ataxin 1
16	214661_s_at	NOP14	NOP14 nucleolar protein homolog (yeast)
17	221923_s_at	LOC399804	nucleophosmin 1 (nucleolar phosphoprotein B23, numatrin) pseudogene 21; hypothetical LOC100131044; similar to nucleophosmin 1
17	221923_s_at	LOC100131044	nucleophosmin 1 (nucleolar phosphoprotein B23, numatrin) pseudogene 21; hypothetical LOC100131044; similar to nucleophosmin 1
17	221923_s_at	LOC729686	nucleophosmin 1 (nucleolar phosphoprotein B23, numatrin) pseudogene 21; hypothetical LOC100131044; similar to nucleophosmin 1
17	221923_s_at	Npm1	nucleophosmin 1 (nucleolar phosphoprotein B23, numatrin) pseudogene 21; hypothetical LOC100131044; similar to nucleophosmin 1
17	221923_s_at	LOC729342	nucleophosmin 1 (nucleolar phosphoprotein B23, numatrin) pseudogene 21; hypothetical LOC100131044; similar to nucleophosmin 1
17	221923_s_at	NPM1P21	nucleophosmin 1 (nucleolar phosphoprotein B23, numatrin) pseudogene 21; hypothetical LOC100131044; similar to nucleophosmin 1
17	221923_s_at	LOC440577	nucleophosmin 1 (nucleolar phosphoprotein B23, numatrin) pseudogene 21; hypothetical LOC100131044; similar to nucleophosmin 1
18	210607_at	FLT3LG	fms-related tyrosine kinase 3 ligand
19	202436_s_at	CYP1B1	cytochrome P450, family 1, subfamily B, polypeptide 1
20	213614_x_at	EEF1A1	eukaryotic translation elongation factor 1 alpha-like 7; eukaryotic translation elongation factor 1 alpha-like 3
20	213614_x_at	EEF1A1P9	eukaryotic translation elongation factor 1 alpha-like 7; eukaryotic translation elongation factor 1 alpha-like 3
20	213614_x_at	EEF1A1P5	eukaryotic translation elongation factor 1 alpha-like 7; eukaryotic translation elongation factor 1 alpha-like 3
20	213614_x_at	EEF1A1P24	eukaryotic translation elongation factor 1 alpha-like 7; eukaryotic

			translation elongation factor 1 alpha-like 3
21	201085_s_at	SON	SON DNA binding protein
22	222348_at	MAST4	microtubule associated serine/threonine kinase family member 4
23	31637_s_at	thrA	thyroid hormone receptor, alpha (erythroblastic leukemia viral (v-erb-a) oncogene homolog, avian)
24	205530_at	Etfdh	electron-transferring-flavoprotein dehydrogenase
25	213817_at	irak3	interleukin-1 receptor-associated kinase 3
26	37590_g_at	ZNF710	zinc finger protein 710
27	221963_x_at	NA	not yet annotated
28	217494_s_at	PTENP1	phosphatase and tensin homolog; phosphatase and tensin homolog pseudogene 1
28	217494_s_at	pten	phosphatase and tensin homolog; phosphatase and tensin homolog pseudogene 1
29	206413_s_at	TCL6	T-cell leukemia/lymphoma 6
30	211282_x_at	TNFRSF25	tumor necrosis factor receptor superfamily, member 25
31	219025_at	CD248	CD248 molecule, endosialin
32	217992_s_at	Efhd2	EF-hand domain family, member D2
33	205594_at	ZNF652	zinc finger protein 652
34	203658_at	SLC25A20	solute carrier family 25 (carnitine/acylcarnitine translocase), member 20
35	222337_at	OSBPL9	oxysterol binding protein-like 9
36	217647_at	dhodh	dihydroorotate dehydrogenase
37	219671_at	HPCAL4	hippocalcin like 4
38	214394_x_at	EEF1D	eukaryotic translation elongation factor 1 delta (guanine nucleotide exchange protein)
39	222091_at	HPCAL4	hippocalcin like 4
40	214180_at	MAN1C1	mannosidase, alpha, class 1C, member 1
41	212831_at	MEGF9	multiple EGF-like-domains 9
42	202388_at	RGS2	regulator of G-protein signaling 2, 24kDa
43	212794_s_at	KIAA1033	KIAA1033
44	41113_at	ZNF500	zinc finger protein 500
45	214500_at	H2AFY	H2A histone family, member Y
46	207078_at	MED6	mediator complex subunit 6
47	220609_at	LOC202181	hypothetical protein LOC202181
48	213428_s_at	COL6A1	collagen, type VI, alpha 1
49	212562_s_at	CTSZ	cathepsin Z
50	219574_at	MARCH1	membrane-associated ring finger (C3HC4) 1
51	220085_at	Hells	helicase, lymphoid-specific
52	201132_at	RPL36A	ribosomal protein L36a pseudogene 51; ribosomal protein L36a pseudogene 37; ribosomal protein L36a pseudogene 49
52	201132_at	RPL36AP51	ribosomal protein L36a pseudogene 51; ribosomal protein L36a pseudogene 37; ribosomal protein L36a pseudogene 49
52	201132_at	RPL36AP49	ribosomal protein L36a pseudogene 51; ribosomal protein L36a pseudogene 37; ribosomal protein L36a pseudogene 49
52	201132_at	Hnrnph2	ribosomal protein L36a pseudogene 51; ribosomal protein L36a pseudogene 37; ribosomal protein L36a pseudogene 49
52	201132_at	RPL36AP37	ribosomal protein L36a pseudogene 51; ribosomal protein L36a pseudogene 37; ribosomal protein L36a pseudogene 49
53	217168_s_at	HERPUD1	homocysteine-inducible, endoplasmic reticulum stress-inducible, ubiquitin-like domain member 1
54	201318_s_at	MYL12B	myosin, light chain 12B, regulatory
55	216682_s_at	fam48a	family with sequence similarity 48, member A
56	220152_at	C10orf95	chromosome 10 open reading frame 95
57	218693_at	tspan15	tetraspanin 15

58	213804_at	INPP5B	inositol polyphosphate-5-phosphatase, 75kDa
59	222280_at	GAPDHS	glyceraldehyde-3-phosphate dehydrogenase, spermatogenic
60	210701_at	cfdp1	craniofacial development protein 1
61	220215_at	ZNF669	zinc finger protein 669
62	207630_s_at	Crem	cAMP responsive element modulator
63	203107_x_at	RPS2P17	ribosomal protein S2 pseudogene 8; ribosomal protein S2 pseudogene 11; ribosomal protein S2 pseudogene 5; ribosomal protein S2 pseudogene 12
63	203107_x_at	rps2	ribosomal protein S2 pseudogene 8; ribosomal protein S2 pseudogene 11; ribosomal protein S2 pseudogene 5; ribosomal protein S2 pseudogene 12
63	203107_x_at	RPS2P55	ribosomal protein S2 pseudogene 8; ribosomal protein S2 pseudogene 11; ribosomal protein S2 pseudogene 5; ribosomal protein S2 pseudogene 12
63	203107_x_at	RPS2P8	ribosomal protein S2 pseudogene 8; ribosomal protein S2 pseudogene 11; ribosomal protein S2 pseudogene 5; ribosomal protein S2 pseudogene 12
63	203107_x_at	RPS2P20	ribosomal protein S2 pseudogene 8; ribosomal protein S2 pseudogene 11; ribosomal protein S2 pseudogene 5; ribosomal protein S2 pseudogene 12
63	203107_x_at	RPS2P11	ribosomal protein S2 pseudogene 8; ribosomal protein S2 pseudogene 11; ribosomal protein S2 pseudogene 5; ribosomal protein S2 pseudogene 12
63	203107_x_at	RPS2P12	ribosomal protein S2 pseudogene 8; ribosomal protein S2 pseudogene 11; ribosomal protein S2 pseudogene 5; ribosomal protein S2 pseudogene 12
63	203107_x_at	RPS2P5	ribosomal protein S2 pseudogene 8; ribosomal protein S2 pseudogene 11; ribosomal protein S2 pseudogene 5; ribosomal protein S2 pseudogene 12
63	203107_x_at	RPS2P51	ribosomal protein S2 pseudogene 8; ribosomal protein S2 pseudogene 11; ribosomal protein S2 pseudogene 5; ribosomal protein S2 pseudogene 12
64	206074_s_at	LOC100130009	hypothetical LOC100130009; high mobility group AT-hook 1
64	206074_s_at	HMGA1	hypothetical LOC100130009; high mobility group AT-hook 1
65	218445_at	h2afy2	H2A histone family, member Y2
66	213159_at	pcnx	pecanex homolog (Drosophila)
67	218676_s_at	PCTP	phosphatidylcholine transfer protein
68	203825_at	BRD3	bromodomain containing 3
69	219632_s_at	TRPV1	transient receptor potential cation channel, subfamily V, member 1
70	203041_s_at	lamp2	lysosomal-associated membrane protein 2
71	206761_at	Cd96	CD96 molecule
72	205174_s_at	QPCT	glutaminyl-peptide cyclotransferase
73	207188_at	Cdk3	cyclin-dependent kinase 3
74	214749_s_at	LOC653354	similar to armadillo repeat containing, X-linked 6; armadillo repeat containing, X-linked 6
74	214749_s_at	Armcx6	similar to armadillo repeat containing, X-linked 6; armadillo repeat containing, X-linked 6
75	202340_x_at	NR4A1	nuclear receptor subfamily 4, group A, member 1
76	203761_at	SLA	Src-like-adaptor
77	212414_s_at	SEPT6	septin 6
78	217645_at	COX16	COX16 cytochrome c oxidase assembly homolog (S. cerevisiae)
79	209810_at	sftpb	surfactant protein B
80	201221_s_at	SNRNP70	small nuclear ribonucleoprotein 70kDa (U1)
81	213006_at	cebpd	CCAAT/enhancer binding protein (C/EBP), delta
82	212934_at	UBXN2B	UBX domain protein 2B
83	208828_at	POLE3	polymerase (DNA directed), epsilon 3 (p17 subunit)
84	206757_at	PDE5A	phosphodiesterase 5A, cGMP-specific
85	217995_at	SQRDL	sulfide quinone reductase-like (yeast)
86	212659_s_at	IL1RN	interleukin 1 receptor antagonist
87	219846_at	YY1AP1	YY1 associated protein 1; gon-4-like (C. elegans)
87	219846_at	GON4L	YY1 associated protein 1; gon-4-like (C. elegans)
88	210047_at	SLC11A2	solute carrier family 11 (proton-coupled divalent metal ion transporters),

			member 2
89	205674_x_at	FXYD2	FXYD domain containing ion transport regulator 2
90	215786_at	rsf1	remodeling and spacing factor 1
91	213641_at	ZNF500	zinc finger protein 500
92	218341_at	PPCS	phosphopantothenoylcysteine synthetase
93	202414_at	ERCC5	excision repair cross-complementing rodent repair deficiency, complementation group 5
94	217671_at	rfx3	regulatory factor X, 3 (influences HLA class II expression)
95	222141_at	Klhl22	kelch-like 22 (Drosophila)
96	201546_at	Trip12	thyroid hormone receptor interactor 12
97	201013_s_at	paics	phosphoribosylaminoimidazole carboxylase, phosphoribosylaminoimidazole succinocarboxamide synthetase
98	201677_at	C3orf37	chromosome 3 open reading frame 37
99	209995_s_at	TCL1A	T-cell leukemia/lymphoma 1A
100	214487_s_at	RAP2A	RAP2A, member of RAS oncogene family
101	204826_at	CCNF	cyclin F
102	217575_s_at	SOS2	son of sevenless homolog 2 (Drosophila)
103	202789_at	PLCG1	phospholipase C, gamma 1
104	209185_s_at	irs2	insulin receptor substrate 2
105	211715_s_at	BDH1	3-hydroxybutyrate dehydrogenase, type 1
106	205129_at	NPM3	nucleophosmin/nucleoplasmin, 3
107	206257_at	CCDC9	coiled-coil domain containing 9
108	214775_at	N4BP3	Nedd4 binding protein 3
109	213340_s_at	KIAA0495	KIAA0495
110	203127_s_at	Sptlc2	serine palmitoyltransferase, long chain base subunit 2
111	205209_at	ACVR1B	activin A receptor, type IB
112	202864_s_at	Sp100	SP100 nuclear antigen
113	220728_at	NA	not yet annotated
114	203630_s_at	COG5	component of oligomeric golgi complex 5
115	212115_at	HN1L	hematological and neurological expressed 1-like
116	220384_at	TXNDC3	thioredoxin domain containing 3 (spermatozoa)
117	206453_s_at	Ndrg2	NDRG family member 2
118	221588_x_at	Aldh6a1	aldehyde dehydrogenase 6 family, member A1
119	213639_s_at	ZNF500	zinc finger protein 500
120	222027_at	Nucks1	nuclear casein kinase and cyclin-dependent kinase substrate 1
121	215390_at	c9orf5	chromosome 9 open reading frame 5
122	215030_at	Grsf1	G-rich RNA sequence binding factor 1
123	202651_at	lpgat1	lysophosphatidylglycerol acyltransferase 1
124	210950_s_at	FDFT1	farnesyl-diphosphate farnesyltransferase 1
125	219315_s_at	TMEM204	transmembrane protein 204
126	217414_x_at	hba2	hemoglobin, alpha 2; hemoglobin, alpha 1
126	217414_x_at	HBA1	hemoglobin, alpha 2; hemoglobin, alpha 1
127	204053_x_at	PTENP1	phosphatase and tensin homolog; phosphatase and tensin homolog pseudogene 1
127	204053_x_at	pten	phosphatase and tensin homolog; phosphatase and tensin homolog pseudogene 1
128	215151_at	dock10	dedicator of cytokinesis 10
129	213462_at	NPAS2	neuronal PAS domain protein 2
130	213008_at	fanci	Fanconi anemia, complementation group I
131	222006_at	LETM1	leucine zipper-EF-hand containing transmembrane protein 1
132	221101_at	NA	not yet annotated
133	209623_at	mccc2	methylcrotonoyl-Coenzyme A carboxylase 2 (beta)
134	205698_s_at	map2k6	mitogen-activated protein kinase kinase 6

135	208843_s_at	gorasp2	golgi reassembly stacking protein 2, 55kDa
136	212192_at	kctd12	potassium channel tetramerisation domain containing 12
137	201581_at	TMX4	thioredoxin-related transmembrane protein 4
138	222071_s_at	SLCO4C1	solute carrier organic anion transporter family, member 4C1
139	205254_x_at	TCF7	transcription factor 7 (T-cell specific, HMG-box)
140	208499_s_at	DNAJC3	DnaJ (Hsp40) homolog, subfamily C, member 3
141	219061_s_at	Lage3	L antigen family, member 3
142	218495_at	UXT	ubiquitously-expressed transcript
143	221797_at	C17orf90	chromosome 17 open reading frame 90
144	204018_x_at	hba2	hemoglobin, alpha 2; hemoglobin, alpha 1
144	204018_x_at	HBA1	hemoglobin, alpha 2; hemoglobin, alpha 1
145	203215_s_at	myo6	myosin VI
146	213922_at	TTBK2	tau tubulin kinase 2
147	206158_s_at	CNBP	CCHC-type zinc finger, nucleic acid binding protein
148	210450_at	LOC90925	hypothetical protein LOC90925
149	213435_at	SATB2	SATB homeobox 2
150	213658_at	ZNF710	zinc finger protein 710
151	203226_s_at	Tspan31	tetraspanin 31
152	218150_at	arl5a	ADP-ribosylation factor-like 5A
153	213666_at	SEPT6	septin 6
154	216043_x_at	cyp20a1	cytochrome P450, family 20, subfamily A, polypeptide 1
155	212542_s_at	PHIP	pleckstrin homology domain interacting protein
156	201198_s_at	PSMD1	proteasome (prosome, macropain) 26S subunit, non-ATPase, 1
157	203549_s_at	Lpl	lipoprotein lipase
158	202658_at	PEX11B	peroxisomal biogenesis factor 11 beta
159	221417_x_at	S1PR5	sphingosine-1-phosphate receptor 5
160	204589_at	NUAK1	NUAK family, SNF1-like kinase, 1
161	212374_at	fem1b	fem-1 homolog b (C. elegans)
162	204900_x_at	SAP30	Sin3A-associated protein, 30kDa
163	212996_s_at	URB1	URB1 ribosome biogenesis 1 homolog (S. cerevisiae)
164	201041_s_at	DUSP1	dual specificity phosphatase 1
165	206210_s_at	cetp	cholesteryl ester transfer protein, plasma
166	217842_at	luc7l2	LUC7-like 2 (S. cerevisiae)
167	212361_s_at	ATP2A2	ATPase, Ca++ transporting, cardiac muscle, slow twitch 2
168	202446_s_at	plscr1	phospholipid scramblase 1
169	219206_x_at	Tmbim4	transmembrane BAX inhibitor motif containing 4
170	220354_at	LOC10013401 8	similar to hCG1774568
171	217670_at	RPLP2P3	ribosomal protein, large, P2 pseudogene 3; ribosomal protein, large, P2
171	217670_at	Rplp2	ribosomal protein, large, P2 pseudogene 3; ribosomal protein, large, P2
172	206980_s_at	FLT3LG	fms-related tyrosine kinase 3 ligand
173	209458_x_at	hba2	hemoglobin, alpha 2; hemoglobin, alpha 1
173	209458_x_at	HBA1	hemoglobin, alpha 2; hemoglobin, alpha 1
174	218845_at	dusp22	similar to mitogen-activated protein kinase phosphatase x; dual specificity phosphatase 22
174	218845_at	LOC10013429 1	similar to mitogen-activated protein kinase phosphatase x; dual specificity phosphatase 22
175	212766_s_at	ISG20L2	interferon stimulated exonuclease gene 20kDa-like 2
176	221221_s_at	KLHL3	kelch-like 3 (Drosophila)
177	213302_at	PFAS	phosphoribosylformylglycinamidine synthase
178	50374_at	C17orf90	chromosome 17 open reading frame 90
179	216751_at	NA	not yet annotated

180	213871_s_at	C6orf108	chromosome 6 open reading frame 108
181	207434_s_at	FXYD2	FXYD domain containing ion transport regulator 2
182	60528_at	JMJD7-PLA2G4B	JMJD7-PLA2G4B readthrough transcript; phospholipase A2, group IVB (cytosolic); jumonji domain containing 7
182	60528_at	JMJD7	JMJD7-PLA2G4B readthrough transcript; phospholipase A2, group IVB (cytosolic); jumonji domain containing 7
182	60528_at	PLA2G4B	JMJD7-PLA2G4B readthrough transcript; phospholipase A2, group IVB (cytosolic); jumonji domain containing 7
183	201382_at	CACYBP	similar to calcyclin binding protein; calcyclin binding protein
183	201382_at	LOC644877	similar to calcyclin binding protein; calcyclin binding protein
184	202163_s_at	cnot8	CCR4-NOT transcription complex, subunit 8
185	217933_s_at	LAP3	leucine aminopeptidase 3
186	202119_s_at	CPNE3	copine III
187	209992_at	Pfkfb2	6-phosphofructo-2-kinase/fructose-2,6-biphosphatase 2
188	210410_s_at	C6orf26	chromosome 6 open reading frame 26
189	209497_s_at	rbm4b	RNA binding motif protein 4B
190	206352_s_at	PEX10	peroxisomal biogenesis factor 10
191	222273_at	PAPOLG	poly(A) polymerase gamma
192	208892_s_at	dusp6	dual specificity phosphatase 6
193	216218_s_at	PLCL2	phospholipase C-like 2
194	206332_s_at	IFI16	interferon, gamma-inducible protein 16
195	204054_at	PTENP1	phosphatase and tensin homolog; phosphatase and tensin homolog pseudogene 1
195	204054_at	pten	phosphatase and tensin homolog; phosphatase and tensin homolog pseudogene 1
196	204555_s_at	PPP1R3D	protein phosphatase 1, regulatory (inhibitor) subunit 3D
197	213582_at	Atp11a	ATPase, class VI, type 11A
198	212582_at	OSBPL8	oxysterol binding protein-like 8
199	215892_at	ZNF440	zinc finger protein 440
200	217871_s_at	LOC284889	hypothetical protein LOC284889
201	201861_s_at	Lrrfip1	leucine rich repeat (in FLII) interacting protein 1
202	217738_at	NAMPT	nicotinamide phosphoribosyltransferase
203	221995_s_at	MRP63	mitochondrial ribosomal protein 63
204	202137_s_at	zmynd11	zinc finger, MYND domain containing 11
205	218213_s_at	c11orf10	chromosome 11 open reading frame 10
206	212919_at	dcp2	DCP2 decapping enzyme homolog (S. cerevisiae)
207	201417_at	Sox4	SRY (sex determining region Y)-box 4
208	211071_s_at	MLLT11	myeloid/lymphoid or mixed-lineage leukemia (trithorax homolog, Drosophila); translocated to, 11
209	210543_s_at	PRKDC	similar to protein kinase, DNA-activated, catalytic polypeptide; protein kinase, DNA-activated, catalytic polypeptide
209	210543_s_at	LOC731751	similar to protein kinase, DNA-activated, catalytic polypeptide; protein kinase, DNA-activated, catalytic polypeptide
210	210098_s_at	NOL7	nucleolar protein 7, 27kDa
211	204386_s_at	MRP63	mitochondrial ribosomal protein 63
212	202539_s_at	HMGCR	3-hydroxy-3-methylglutaryl-Coenzyme A reductase
213	201987_at	MED13	mediator complex subunit 13
214	202753_at	PSMD6	proteasome (prosome, macropain) 26S subunit, non-ATPase, 6
215	218807_at	VAV3	vav 3 guanine nucleotide exchange factor
216	211699_x_at	hba2	hemoglobin, alpha 2; hemoglobin, alpha 1
216	211699_x_at	HBA1	hemoglobin, alpha 2; hemoglobin, alpha 1
217	212300_at	TXLNA	taxilin alpha
218	205181_at	ZNF193	zinc finger protein 193

219	211799_x_at	HLA-C	major histocompatibility complex, class I, C; major histocompatibility complex, class I, B
219	211799_x_at	HLA-B	major histocompatibility complex, class I, C; major histocompatibility complex, class I, B
220	204530_s_at	Tox	thymocyte selection-associated high mobility group box
221	AFFX-r2-Ec-bioB-M_at		
222	215105_at	cg030	hypothetical CG030
223	216230_x_at	SMPD1	sphingomyelin phosphodiesterase 1, acid lysosomal
224	204909_at	DDX6	DEAD (Asp-Glu-Ala-Asp) box polypeptide 6
225	213798_s_at	CAP1	CAP, adenylate cyclase-associated protein 1 (yeast)
226	209922_at	BRAP	BRCA1 associated protein
227	214801_at	NA	not yet annotated
228	221840_at	PTPRE	protein tyrosine phosphatase, receptor type, E
229	209633_at	Ppp2r3a	protein phosphatase 2 (formerly 2A), regulatory subunit B", alpha
230	209960_at	Hgf	hepatocyte growth factor (hepapoietin A; scatter factor)
231	210050_at	TPI1	TPI1 pseudogene; triosephosphate isomerase 1
231	210050_at	TPI1P1	TPI1 pseudogene; triosephosphate isomerase 1
232	220954_s_at	PILRB	paired immunoglobin-like type 2 receptor beta
233	204516_at	ATXN7	ataxin 7
234	209702_at	FTO	fat mass and obesity associated
235	202393_s_at	KLF10	Kruppel-like factor 10
236	210896_s_at	asph	aspartate beta-hydroxylase
237	204091_at	PDE6D	phosphodiesterase 6D, cGMP-specific, rod, delta
238	202005_at	ST14	suppression of tumorigenicity 14 (colon carcinoma)
239	41220_at	SEPT9	septin 9
240	218958_at	C19orf60	chromosome 19 open reading frame 60
241	208580_x_at	Hist1h4c	histone cluster 1, H4l; histone cluster 1, H4k; histone cluster 4, H4; histone cluster 1, H4h; histone cluster 1, H4j; histone cluster 1, H4i
241	208580_x_at	Hist1h4d	histone cluster 1, H4l; histone cluster 1, H4k; histone cluster 4, H4; histone cluster 1, H4h; histone cluster 1, H4j; histone cluster 1, H4i
241	208580_x_at	Hist1h4f	histone cluster 1, H4l; histone cluster 1, H4k; histone cluster 4, H4; histone cluster 1, H4h; histone cluster 1, H4j; histone cluster 1, H4i
241	208580_x_at	Hist1h4h	histone cluster 1, H4l; histone cluster 1, H4k; histone cluster 4, H4; histone cluster 1, H4h; histone cluster 1, H4j; histone cluster 1, H4i
241	208580_x_at	Hist1h4i	histone cluster 1, H4l; histone cluster 1, H4k; histone cluster 4, H4; histone cluster 1, H4h; histone cluster 1, H4j; histone cluster 1, H4i
241	208580_x_at	Hist1h4j	histone cluster 1, H4l; histone cluster 1, H4k; histone cluster 4, H4; histone cluster 1, H4h; histone cluster 1, H4j; histone cluster 1, H4i
241	208580_x_at	HIST2H4A	histone cluster 1, H4l; histone cluster 1, H4k; histone cluster 4, H4; histone cluster 1, H4h; histone cluster 1, H4j; histone cluster 1, H4i
241	208580_x_at	Hist1h4a	histone cluster 1, H4l; histone cluster 1, H4k; histone cluster 4, H4; histone cluster 1, H4h; histone cluster 1, H4j; histone cluster 1, H4i
241	208580_x_at	Hist1h4b	histone cluster 1, H4l; histone cluster 1, H4k; histone cluster 4, H4; histone cluster 1, H4h; histone cluster 1, H4j; histone cluster 1, H4i
241	208580_x_at	HIST2H4B	histone cluster 1, H4l; histone cluster 1, H4k; histone cluster 4, H4; histone cluster 1, H4h; histone cluster 1, H4j; histone cluster 1, H4i
241	208580_x_at	HIST1H4E	histone cluster 1, H4l; histone cluster 1, H4k; histone cluster 4, H4; histone cluster 1, H4h; histone cluster 1, H4j; histone cluster 1, H4i
241	208580_x_at	Hist1h4k	histone cluster 1, H4l; histone cluster 1, H4k; histone cluster 4, H4; histone cluster 1, H4h; histone cluster 1, H4j; histone cluster 1, H4i
241	208580_x_at	hist1h4l	histone cluster 1, H4l; histone cluster 1, H4k; histone cluster 4, H4; histone cluster 1, H4h; histone cluster 1, H4j; histone cluster 1, H4i
241	208580_x_at	Hist4h4	histone cluster 1, H4l; histone cluster 1, H4k; histone cluster 4, H4; histone

			cluster 1, H4h; histone cluster 1, H4j; histone cluster 1, H4i
242	213818_x_at	Col5a1	collagen, type V, alpha 1
243	200732_s_at	ptp4a1	protein tyrosine phosphatase type IVA, member 1
244	204094_s_at	TSC22D2	TSC22 domain family, member 2
245	209030_s_at	CADM1	cell adhesion molecule 1
246	213390_at	Zc3h4	zinc finger CCCH-type containing 4
247	219696_at	DENND1B	DENN/MADD domain containing 1B
248	213168_at	SP3	Sp3 transcription factor
249	222119_s_at	fbxo11	F-box protein 11
250	209759_s_at	dci	dodecenoyl-Coenzyme A delta isomerase (3,2 trans-enoyl-Coenzyme A isomerase)
251	221741_s_at	YTHDF1	YTH domain family, member 1
252	203739_at	ZNF217	zinc finger protein 217
253	209405_s_at	FAM3A	family with sequence similarity 3, member A
254	212916_at	PHF8	PHD finger protein 8
255	213883_s_at	TM2D1	TM2 domain containing 1
256	217765_at	nrbp1	nuclear receptor binding protein 1
257	212263_at	QKI	quaking homolog, KH domain RNA binding (mouse)
258	209899_s_at	PUF60	poly-U binding splicing factor 60KDa
259	210350_x_at	ing1	inhibitor of growth family, member 1
260	200731_s_at	ptp4a1	protein tyrosine phosphatase type IVA, member 1
261	212569_at	SMCHD1	structural maintenance of chromosomes flexible hinge domain containing 1
262	216095_x_at	MTMR1	myotubularin related protein 1
263	213341_at	FEM1C	fem-1 homolog c (C. elegans)
264	218530_at	fhod1	formin homology 2 domain containing 1
265	208865_at	csnk1a1	casein kinase 1, alpha 1
266	202041_s_at	FIBP	fibroblast growth factor (acidic) intracellular binding protein
267	221613_s_at	zfand6	zinc finger, AN1-type domain 6
268	221437_s_at	MRPS15	mitochondrial ribosomal protein S15
269	204104_at	Snapc2	small nuclear RNA activating complex, polypeptide 2, 45kDa
270	203422_at	Pold1	polymerase (DNA directed), delta 1, catalytic subunit 125kDa
271	216221_s_at	PUM2	pumilio homolog 2 (Drosophila)
272	219157_at	KLHL2	kelch-like 2, Mayven (Drosophila)
273	202417_at	keap1	kelch-like ECH-associated protein 1
274	212824_at	FUBP3	far upstream element (FUSE) binding protein 3
275	200097_s_at	LOC644063	heterogeneous nuclear ribonucleoprotein K; similar to heterogeneous nuclear ribonucleoprotein K
275	200097_s_at	Hnrnpk	heterogeneous nuclear ribonucleoprotein K; similar to heterogeneous nuclear ribonucleoprotein K
276	201641_at	BST2	NPC-A-7; bone marrow stromal cell antigen 2
277	202991_at	stard3	StAR-related lipid transfer (START) domain containing 3
278	213511_s_at	MTMR1	myotubularin related protein 1
279	205917_at	ZNF264	zinc finger protein 264
280	221952_x_at	trmt5	TRM5 tRNA methyltransferase 5 homolog (S. cerevisiae)
281	214076_at	gfod2	glucose-fructose oxidoreductase domain containing 2
282	201310_s_at	C5orf13	chromosome 5 open reading frame 13
283	211336_x_at	LILRB1	leukocyte immunoglobulin-like receptor, subfamily B (with TM and ITIM domains), member 1
284	221596_s_at	c7orf64	chromosome 7 open reading frame 64
285	203330_s_at	STX5	syntaxin 5
286	222335_at	NA	not yet annotated
287	201251_at	LOC652797	similar to Pyruvate kinase, isozymes M1/M2 (Pyruvate kinase muscle

			isozyme) (Cytosolic thyroid hormone-binding protein) (CTHBP) (THBP1)
287	201251_at	PKM2	similar to Pyruvate kinase, isozymes M1/M2 (Pyruvate kinase muscle isozyme) (Cytosolic thyroid hormone-binding protein) (CTHBP) (THBP1)
288	32836_at	AGPAT1	1-acylglycerol-3-phosphate O-acyltransferase 1 (lysophosphatidic acid acyltransferase, alpha)
289	214794_at	PA2G4	proliferation-associated 2G4, 38kDa; proliferation-associated 2G4 pseudogene 4
289	214794_at	PA2G4P4	proliferation-associated 2G4, 38kDa; proliferation-associated 2G4 pseudogene 4
290	212722_s_at	JMJD6	jumonji domain containing 6
291	213453_x_at	LOC10013304 2	glyceraldehyde-3-phosphate dehydrogenase-like 6; hypothetical protein LOC100133042; glyceraldehyde-3-phosphate dehydrogenase
291	213453_x_at	GAPDH	glyceraldehyde-3-phosphate dehydrogenase-like 6; hypothetical protein LOC100133042; glyceraldehyde-3-phosphate dehydrogenase
291	213453_x_at	GAPDHL6	glyceraldehyde-3-phosphate dehydrogenase-like 6; hypothetical protein LOC100133042; glyceraldehyde-3-phosphate dehydrogenase
292	216109_at	Med13l	mediator complex subunit 13-like
293	221771_s_at	Mphosph8	M-phase phosphoprotein 8
294	209051_s_at	RALGDS	ral guanine nucleotide dissociation stimulator
295	218443_s_at	DAZAP1	DAZ associated protein 1
296	212927_at	smc5	structural maintenance of chromosomes 5
297	215597_x_at	MYST4	MYST histone acetyltransferase (monocytic leukemia) 4
298	37254_at	ZNF133	zinc finger protein 133
299	217923_at	pef1	penta-EF-hand domain containing 1
300	217929_s_at	KIAA0319L	KIAA0319-like
301	206133_at	XAF1	XIAP associated factor 1
302	214731_at	CTTNBP2NL	CTTNBP2 N-terminal like
303	215374_at	Papola	poly(A) polymerase alpha
304	202545_at	PRKCD	protein kinase C, delta
305	200048_s_at	Jtb	jumping translocation breakpoint
306	218772_x_at	tmem38b	transmembrane protein 38B
307	209105_at	NCOA1	nuclear receptor coactivator 1
308	206207_at	clc	Charcot-Leyden crystal protein
309	219825_at	CYP26B1	cytochrome P450, family 26, subfamily B, polypeptide 1
310	202956_at	ARFGEF1	ADP-ribosylation factor guanine nucleotide-exchange factor 1(brefeldin A-inhibited)
311	219256_s_at	SH3TC1	SH3 domain and tetratricopeptide repeats 1
312	204176_at	KLHL20	kelch-like 20 (Drosophila)
313	201113_at	Tufm	Tu translation elongation factor, mitochondrial
314	215544_s_at	UBOX5	U-box domain containing 5
315	218995_s_at	EDN1	endothelin 1
316	219402_s_at	Derl1	Der1-like domain family, member 1
317	207418_s_at	DDO	D-aspartate oxidase
318	221902_at	GPR153	G protein-coupled receptor 153
319	217371_s_at	IL15	interleukin 15
320	212175_s_at	Ak2	adenylate kinase 2
321	202643_s_at	TNFAIP3	tumor necrosis factor, alpha-induced protein 3
322	209808_x_at	ing1	inhibitor of growth family, member 1
323	221971_x_at	AGAP6	ArfGAP with GTPase domain, ankyrin repeat and PH domain 4; ArfGAP with GTPase domain, ankyrin repeat and PH domain 6
323	221971_x_at	AGAP4	ArfGAP with GTPase domain, ankyrin repeat and PH domain 4; ArfGAP with GTPase domain, ankyrin repeat and PH domain 6
324	212343_at	YIPF6	Yip1 domain family, member 6

325	201036_s_at	hadh	hydroxyacyl-Coenzyme A dehydrogenase
326	33494_at	Etfdh	electron-transferring-flavoprotein dehydrogenase
327	212462_at	MYST4	MYST histone acetyltransferase (monocytic leukemia) 4
328	218273_s_at	Pdp1	pyruvate dehyrogenase phosphatase catalytic subunit 1
329	213114_at	RER1	RER1 retention in endoplasmic reticulum 1 homolog (S. cerevisiae)
330	203545_at	ALG8	asparagine-linked glycosylation 8, alpha-1,3-glucosyltransferase homolog (S. cerevisiae)
331	220052_s_at	tinf2	TERF1 (TRF1)-interacting nuclear factor 2
332	214722_at	NOTCH2NL	Notch homolog 2 (Drosophila) N-terminal like
333	203746_s_at	HCCS	holocytochrome c synthase (cytochrome c heme-lyase)
334	219617_at	c2orf34	chromosome 2 open reading frame 34
335	218381_s_at	U2AF2	U2 small nuclear RNA auxiliary factor 2
336	210097_s_at	NOL7	nucleolar protein 7, 27kDa
337	221290_s_at	Mum1	melanoma associated antigen (mutated) 1
338	33307_at	RRP7B	ribosomal RNA processing 7 homolog B (S. cerevisiae); ribosomal RNA processing 7 homolog A (S. cerevisiae)
338	33307_at	RRP7A	ribosomal RNA processing 7 homolog B (S. cerevisiae); ribosomal RNA processing 7 homolog A (S. cerevisiae)
339	217537_x_at	NA	not yet annotated
340	210756_s_at	Notch2	Notch homolog 2 (Drosophila)
341	202882_x_at	NOL7	nucleolar protein 7, 27kDa
342	209226_s_at	tnpo1	transportin 1
343	207926_at	GP5	glycoprotein V (platelet)
344	221808_at	RAB9A	RAB9A, member RAS oncogene family
345	206746_at	bfsp1	beaded filament structural protein 1, filensin
346	211310_at	EZH1	enhancer of zeste homolog 1 (Drosophila)
347	211745_x_at	hba2	hemoglobin, alpha 2; hemoglobin, alpha 1
347	211745_x_at	HBA1	hemoglobin, alpha 2; hemoglobin, alpha 1
348	210466_s_at	serbp1	SERPINE1 mRNA binding protein 1
349	213852_at	RBM8A	RNA binding motif protein 8A
350	217027_x_at	KPNB1	karyopherin (importin) beta 1
351	211678_s_at	RNF114	ring finger protein 114
352	207357_s_at	GALNT10	UDP-N-acetyl-alpha-D-galactosamine:polypeptide N-acetylgalactosaminyltransferase 10 (GalNAc-T10)
353	AFFX-r2-Ec-bioB-5_at		
354	207076_s_at	ASS1	argininosuccinate synthetase 1
355	220835_s_at	ZNF407	zinc finger protein 407
356	208777_s_at	Psmd11	proteasome (prosome, macropain) 26S subunit, non-ATPase, 11
357	212071_s_at	SPTBN1	spectrin, beta, non-erythrocytic 1
358	207104_x_at	LILRB1	leukocyte immunoglobulin-like receptor, subfamily B (with TM and ITIM domains), member 1
359	216304_x_at	YME1L1	YME1-like 1 (S. cerevisiae)
360	212594_at	PDCD4	programmed cell death 4 (neoplastic transformation inhibitor)
361	201040_at	gnai2	guanine nucleotide binding protein (G protein), alpha inhibiting activity polypeptide 2
362	203388_at	arrb2	arrestin, beta 2
363	209584_x_at	APOBEC3C	apolipoprotein B mRNA editing enzyme, catalytic polypeptide-like 3C
364	218963_s_at	Krt23	keratin 23 (histone deacetylase inducible)
365	218389_s_at	aph1a	anterior pharynx defective 1 homolog A (C. elegans)
366	204686_at	IRS1	insulin receptor substrate 1
367	222062_at	IL27RA	interleukin 27 receptor, alpha
368	219459_at	Polr3b	polymerase (RNA) III (DNA directed) polypeptide B

369	206548_at	NA	not yet annotated
370	215648_at	NUDCD3	NudC domain containing 3
371	209602_s_at	GATA3	GATA binding protein 3
372	202617_s_at	mecp2	methyl CpG binding protein 2 (Rett syndrome)
373	204553_x_at	INPP4A	inositol polyphosphate-4-phosphatase, type I, 107kDa
374	220120_s_at	EPB41L4A	erythrocyte membrane protein band 4.1 like 4A
375	218991_at	HEATR6	HEAT repeat containing 6
376	210685_s_at	Ube4b	ubiquitination factor E4B (UFD2 homolog, yeast)
377	201206_s_at	RRBP1	ribosome binding protein 1 homolog 180kDa (dog)
378	209403_at	TBC1D3C	TBC1 domain family, member 3E; TBC1 domain family, member 3F; TBC1 domain family, member 3G
378	209403_at	TBC1D3F	TBC1 domain family, member 3E; TBC1 domain family, member 3F; TBC1 domain family, member 3G
378	209403_at	TBC1D3G	TBC1 domain family, member 3E; TBC1 domain family, member 3F; TBC1 domain family, member 3G
378	209403_at	TBC1D3H	TBC1 domain family, member 3E; TBC1 domain family, member 3F; TBC1 domain family, member 3G
378	209403_at	LOC653380	TBC1 domain family, member 3E; TBC1 domain family, member 3F; TBC1 domain family, member 3G
378	209403_at	TBC1D3	TBC1 domain family, member 3E; TBC1 domain family, member 3F; TBC1 domain family, member 3G
378	209403_at	TBC1D3B	TBC1 domain family, member 3E; TBC1 domain family, member 3F; TBC1 domain family, member 3G
379	210337_s_at	ACLY	ATP citrate lyase
380	214973_x_at	IGHD	immunoglobulin heavy constant delta
381	207252_at	INE1	inactivation escape 1 (non-protein coding)
382	204415_at	IFI6	interferon, alpha-inducible protein 6
383	207705_s_at	NINL	ninein-like
384	204098_at	Rbmx2	RNA binding motif protein, X-linked 2
385	205963_s_at	DNAJA3	DnaJ (Hsp40) homolog, subfamily A, member 3
386	205660_at	OASL	2'-5'-oligoadenylate synthetase-like
387	203975_s_at	CHAF1A	chromatin assembly factor 1, subunit A (p150)
388	217170_at	TRAV8-6	T cell receptor alpha variable 8-6
389	201880_at	ARIH1	ariadne homolog, ubiquitin-conjugating enzyme E2 binding protein, 1 (Drosophila)
390	218400_at	Oas3	2'-5'-oligoadenylate synthetase 3, 100kDa
391	205239_at	AREGB	amphiregulin; amphiregulin B
391	205239_at	AREG	amphiregulin; amphiregulin B
392	212846_at	Rrp1b	ribosomal RNA processing 1 homolog B (S. cerevisiae)
393	219968_at	ZNF589	zinc finger protein 589
394	209425_at	C1qtnf3	C1q and tumor necrosis factor related protein 3; alpha-methylacyl-CoA racemase
394	209425_at	AMACR	C1q and tumor necrosis factor related protein 3; alpha-methylacyl-CoA racemase
395	221169_s_at	HRH4	histamine receptor H4
