# Supplementary material for: Blood transcriptomics of drug-naïve sporadic Parkinson’s disease patients
Source: BMC Genomics. 2015 Oct 28;16:876. doi: 10.1186/s12864-015-2058-3 (PMC4625854; doi:10.1186/s12864-015-2058-3)
Supplement: Additional file 4: — Square of the Mahalanobis distance calculated for each sample from both control and pathological class models. (PDF 3122 kb) [file 12864_2015_2058_MOESM4_ESM.pdf]

Additional file 4. Square of the Mahalanobis distance calculated for each sample from both the control and the pathological class models.

	Control	Pathological
**C001MB0C**	1.23	19.10
**C002MC0C**	7.91	19.66
**C003GC0C**	4.35	33.03
**C004GQ0C**	5.10	27.42
**C005MZ0C**	8.05	46.87
**C006ZB0C**	1.48	17.65
**C007MC0C**	2.38	26.33
**C008MB0C**	2.28	22.25
**C009GM0C**	6.80	25.94
**C011GB0C**	3.60	30.29
**C012IK0C**	4.26	33.48
**C013FB0C**	4.87	10.45
**C014MF0C**	1.65	18.86
**C015PS0C**	2.64	21.42
**C016BF0C**	5.62	36.56
**C017AS0C**	9.06	18.65
**C018LC0C**	4.30	34.36
**C019AP0C**	9.01	25.61
**C020MB0C**	10.16	33.30
**P001RS01**	30.83	3.26
**P002FM01**	28.31	8.84
**P003ZA01**	14.27	3.39
**P004LN01**	23.04	4.97
**P005PN01**	21.89	1.16
**P006LS01**	33.78	5.33
**P007US01**	30.77	4.83
**P009WB01**	25.75	1.60
**P010SK01**	28.66	4.41
**P011MF01**	11.81	4.74
**P012MB01**	29.49	4.78
**P013SR01**	54.63	11.57
**P014GS01**	31.74	4.21

**P015MS01**	14.58	4.57
**P016MG01**	52.28	12.13
**P017PB01**	26.94	1.62
**P018RD01**	24.33	2.85
**P019MT01**	17.61	3.70
**P020FG01**	16.85	2.85
**P021LK01**	13.48	3.94
**P022ZP01**	18.79	1.46
**P023PP01**	29.60	6.57
**P024BS01**	14.68	1.49
**P025FS01**	22.37	0.41
**P026LB01**	29.87	2.95
**P027MM01**	18.60	4.78
**P028DL01**	13.11	8.20
**P029EP01**	18.90	1.98
**P030AZ01**	39.20	4.93
**P031MC01**	32.73	5.05
**P032AS01**	24.57	3.00
**P033CG01**	25.37	6.71
**P034LM01**	35.77	5.90
**P035NH01**	27.55	2.37
**P036LL01**	23.64	6.65
**P037MC01**	54.58	9.21
**P038SS01**	29.99	1.49
**P039GF01**	25.39	3.54
**P040GG01**	19.90	8.67
**P041BB01**	10.50	10.19
