# Supplementary material for: Blood transcriptomics of drug-naïve sporadic Parkinson’s disease patients
Source: BMC Genomics. 2015 Oct 28;16:876. doi: 10.1186/s12864-015-2058-3 (PMC4625854; doi:10.1186/s12864-015-2058-3)
Supplement: Additional file 5: — Main processes (GOTERM_BP_FAT as classified by DAVID Gene Ontology) altered in whole peripheral blood cells in the 395 selected variables comparing PD patients and controls. GO annotations with at least 3 genes and P < 0.05 (Fisher exact probability) are presented. Count: number of genes involved in the term; %: percentage of involved genes/total genes; P-Value: modified fisher exact P-value, EASE Score; Benjamini: adjusted P-value using Benjamini-Hochberg procedure. (PDF 3122 kb) [file 12864_2015_2058_MOESM5_ESM.pdf]

Additional file 5. Main function (GOTERM_BP_FAT as classified by DAVID gene ontology) altered in whole peripheral blood cells in the 395 selected variables comparing PD patients and controls. GO annotations with at least 3 genes and P<0.05 (Fisher exact probability) are presented. Count: number of genes involved in the term; %: percentage of involved genes/total genes; P-Value: modified fisher exact P-value, EASE Score; Benjamini: adjusted P-value using Benjamini-Hochberg procedure.

**Term**	**Count**	**%**	**P-Value**	**Benjamini**
GO:0006333~chromatin assembly or disassembly	11	3.05	3.23E-04	4.53E-01
GO:0042981~regulation of apoptosis	31	8.59	1.42E-03	7.34E-01
GO:0043067~regulation of programmed cell death	31	8.59	1.65E-03	6.42E-01
GO:0043065~positive regulation of apoptosis	20	5.54	1.72E-03	5.52E-01
GO:0010941~regulation of cell death	31	8.59	1.74E-03	4.77E-01
GO:0044092~negative regulation of molecular function	17	4.71	1.75E-03	4.20E-01
GO:0043068~positive regulation of programmed cell death	20	5.54	1.86E-03	3.91E-01
GO:0010942~positive regulation of cell death	20	5.54	1.96E-03	3.67E-01
GO:0031497~chromatin assembly	8	2.22	2.24E-03	3.71E-01
GO:0065004~protein-DNA complex assembly	8	2.22	2.89E-03	4.17E-01
GO:0034728~nucleosome organization	8	2.22	3.27E-03	4.26E-01
GO:0030098~lymphocyte differentiation	8	2.22	5.74E-03	5.91E-01
GO:0043154~negative regulation of caspase activity	4	1.11	5.80E-03	5.66E-01
GO:0002521~leukocyte differentiation	9	2.49	6.14E-03	5.60E-01
GO:0043407~negative regulation of MAP kinase activity	5	1.39	6.44E-03	5.52E-01
GO:0001775~cell activation	14	3.88	7.29E-03	5.73E-01
GO:0051100~negative regulation of binding	6	1.66	7.60E-03	5.67E-01
GO:0043549~regulation of kinase activity	16	4.43	8.00E-03	5.65E-01
GO:0006334~nucleosome assembly	7	1.94	8.14E-03	5.51E-01
GO:0007568~aging	8	2.22	8.15E-03	5.34E-01
GO:0046649~lymphocyte activation	11	3.05	8.96E-03	5.50E-01
GO:0042325~regulation of phosphorylation	19	5.26	8.99E-03	5.35E-01
GO:0010466~negative regulation of peptidase activity	4	1.11	9.03E-03	5.20E-01
GO:0031328~positive regulation of cellular biosynthetic process	25	6.93	9.04E-03	5.06E-01
GO:0009891~positive regulation of biosynthetic process	25	6.93	1.07E-02	5.50E-01
GO:0031647~regulation of protein stability	5	1.39	1.11E-02	5.51E-01
GO:0006323~DNA packaging	8	2.22	1.12E-02	5.42E-01
GO:0030217~T cell differentiation	6	1.66	1.13E-02	5.32E-01
GO:0051338~regulation of transferase activity	16	4.43	1.15E-02	5.25E-01
GO:0010604~positive regulation of macromolecule metabolic process	29	8.03	1.22E-02	5.34E-01
GO:0045321~leukocyte activation	12	3.32	1.28E-02	5.38E-01
GO:0051174~regulation of phosphorus metabolic process	19	5.26	1.31E-02	5.37E-01
GO:0019220~regulation of phosphate metabolic process	19	5.26	1.31E-02	5.37E-01

GO:0043086~negative regulation of catalytic activity	13	3.60	1.35E-02	5.35E-01
GO:0010648~negative regulation of cell communication	12	3.32	1.51E-02	5.65E-01
GO:0016311~dephosphorylation	9	2.49	1.54E-02	5.63E-01
GO:0008219~cell death	25	6.93	1.55E-02	5.54E-01
GO:0016265~death	25	6.93	1.64E-02	5.66E-01
GO:0006917~induction of apoptosis	14	3.88	1.68E-02	5.65E-01
GO:0012501~programmed cell death	22	6.09	1.71E-02	5.61E-01
GO:0012502~induction of programmed cell death	14	3.88	1.73E-02	5.56E-01
GO:0009968~negative regulation of signal transduction	11	3.05	1.76E-02	5.54E-01
GO:0008610~lipid biosynthetic process	14	3.88	1.81E-02	5.56E-01
GO:0010557~positive regulation of macromolecule biosynthetic process	23	6.37	1.83E-02	5.51E-01
GO:0008283~cell proliferation	17	4.71	2.07E-02	5.87E-01
GO:0044242~cellular lipid catabolic process	6	1.66	2.11E-02	5.87E-01
GO:0006470~protein amino acid dephosphorylation	8	2.22	2.14E-02	5.83E-01
GO:0043392~negative regulation of DNA binding	5	1.39	2.15E-02	5.77E-01
GO:0045934~negative regulation of nucleobase, nucleoside, nucleotide and nucleic acid metabolic process	19	5.26	2.17E-02	5.74E-01
GO:0042127~regulation of cell proliferation	26	7.20	2.37E-02	5.99E-01
GO:0051172~negative regulation of nitrogen compound metabolic process	19	5.26	2.42E-02	5.99E-01
GO:0019637~organophosphate metabolic process	10	2.77	2.45E-02	5.96E-01
GO:0051276~chromosome organization	18	4.99	2.57E-02	6.07E-01
GO:0008202~steroid metabolic process	10	2.77	2.59E-02	6.03E-01
GO:0007167~enzyme linked receptor protein signaling pathway	14	3.88	2.73E-02	6.15E-01
GO:0006325~chromatin organization	15	4.16	2.75E-02	6.12E-01
GO:0043405~regulation of MAP kinase activity	8	2.22	2.83E-02	6.15E-01
GO:0051173~positive regulation of nitrogen compound metabolic process	22	6.09	2.88E-02	6.15E-01
GO:0048384~retinoic acid receptor signaling pathway	3	0.83	2.90E-02	6.12E-01
GO:0051252~regulation of RNA metabolic process	50	13.85	3.25E-02	6.48E-01
GO:0006007~glucose catabolic process	5	1.39	3.26E-02	6.43E-01
GO:0010608~posttranscriptional regulation of gene expression	10	2.77	3.30E-02	6.41E-01
GO:0010605~negative regulation of macromolecule metabolic process	24	6.65	3.30E-02	6.35E-01
GO:0043631~RNA polyadenylation	3	0.83	3.34E-02	6.34E-01
GO:0007049~cell cycle	25	6.93	3.42E-02	6.37E-01
GO:0006469~negative regulation of protein kinase activity	6	1.66	3.52E-02	6.42E-01
GO:0010906~regulation of glucose metabolic process	4	1.11	3.59E-02	6.44E-01
GO:0045935~positive regulation of nucleobase, nucleoside, nucleotide and nucleic acid metabolic process	21	5.82	3.74E-02	6.54E-01
GO:0006396~RNA processing	19	5.26	3.77E-02	6.51E-01
GO:0033673~negative regulation of kinase activity	6	1.66	3.99E-02	6.67E-01
GO:0006006~glucose metabolic process	8	2.22	4.13E-02	6.74E-01

GO:0001959~regulation of cytokine-mediated signaling pathway	3	0.83	4.29E-02	6.83E-01
GO:0008203~cholesterol metabolic process	6	1.66	4.31E-02	6.81E-01
GO:0010675~regulation of cellular carbohydrate metabolic process	4	1.11	4.42E-02	6.85E-01
GO:0048534~hemopoietic or lymphoid organ development	11	3.05	4.62E-02	6.96E-01
GO:0006644~phospholipid metabolic process	9	2.49	4.64E-02	6.93E-01
GO:0016071~mRNA metabolic process	14	3.88	4.65E-02	6.89E-01
GO:0006109~regulation of carbohydrate metabolic process	4	1.11	4.72E-02	6.90E-01
GO:0006915~apoptosis	20	5.54	4.75E-02	6.88E-01
GO:0045941~positive regulation of transcription	19	5.26	4.85E-02	6.91E-01
GO:0009123~nucleoside monophosphate metabolic process	5	1.39	4.87E-02	6.88E-01
