# Supplementary material for: Blood transcriptomics of drug-naïve sporadic Parkinson’s disease patients
Source: BMC Genomics. 2015 Oct 28;16:876. doi: 10.1186/s12864-015-2058-3 (PMC4625854; doi:10.1186/s12864-015-2058-3)
Supplement: Additional file 6: — List of differentially expressed genes between PD patients and controls based on PUMA. Ratios are calculated as differences in gene expression between samples of PD patients and controls, with the top up-regulated genes first. (PDF 3122 kb) [file 12864_2015_2058_MOESM6_ESM.pdf]

Additional file 6. List of differentially expressed genes between PD patients and controls based on PUMA. Ratios are calculated as differences in gene expression between samples of PD patients and controls, with the top up-regulated genes first.

Affymetrix probe ID	Gene name	Gene symbol	Fold change (LOG2)	p-Values
209715_at	chromobox homolog 5 (HP1 alpha homolog, Drosophila)	CBX5	-0.466356561	5.66874E-08
214770_at	macrophage scavenger receptor 1	MSR1	1.155707168	1.03419E-06
216168_at	not yet annotated	NA	-0.877227057	1.28714E-05
204621_s_at	nuclear receptor subfamily 4, group A, member 2	NR4A2	0.604488377	1.84149E-05
209719_x_at	serpin peptidase inhibitor, clade B (ovalbumin), member 3	SERPINB3	0.947560037	2.56296E-05
214487_s_at	RAP2A, member of RAS oncogene family /// RAP2B, member of RAS oncogene family	RAP2A /// RAP2B	0.801631943	3.72848E-05
207652_s_at	chemokine-like receptor 1	CMKLR1	0.471898613	5.14479E-05
208342_x_at	chorionic somatomammotropin hormone 1 (placental lactogen) /// chorionic somatomammotropin hormone 2 /// growth hormone 1	CSH1 /// CSH2 /// GH1	-1.00448384	6.56904E-05
208893_s_at	dual specificity phosphatase 6	DUSP6	0.453386791	7.4754E-05
210085_s_at	annexin A9	ANXA9	-1.14117166	8.53377E-05
209153_s_at	transcription factor 3 (E2A immunoglobulin enhancer binding factors E12/E47)	TCF3	-0.328005864	8.6175E-05
206466_at	acyl-CoA synthetase bubblegum family member 1	ACSBG1	-0.476174167	0.000100777
203645_s_at	CD163 molecule	CD163	0.335349142	0.000103353
213006_at	CCAAT/enhancer binding protein (C/EBP), delta	CEBPD	0.394699181	0.000127742
203658_at	solute carrier family 25 (carnitine/acylcarnitine translocase), member 20	SLC25A20	0.338656917	0.00013186
214015_at	suppressor of cytokine signaling 7	SOCS7	0.934011822	0.00013723
218906_x_at	kinesin light chain 2	KLC2	-0.547975676	0.000179074
214921_at	potassium large conductance calcium-activated channel, subfamily M, alpha member 1	KCNMA1	0.907252494	0.000186009
202436_s_at	cytochrome P450, family 1, subfamily B, polypeptide 1	CYP1B1	0.528002989	0.000197309
202170_s_at	aminoadipate-semialdehyde dehydrogenase-phosphopantetheinyl transferase	AASDHPPT	-0.200729462	0.000217083
209226_s_at	transportin 1	TNPO1	0.146621612	0.000226022
202570_s_at	discs, large (Drosophila) homolog-associated protein 4	DLGAP4	0.329113612	0.0002271
201085_s_at	SON DNA binding protein	SON	0.234788726	0.000228818
204530_s_at	thymocyte selection-associated high mobility group box	TOX	0.364166554	0.000241607
215151_at	dedicator of cytokinesis 10	DOCK10	-0.303841791	0.000247385

221592_at	TBC1 domain family, member 8 (with GRAM domain)	TBC1D8	0.352432876	0.000247568
206720_at	mannosyl (alpha-1,6-)-glycoprotein beta-1,6-N-acetyl-glucosaminyltransferase	MGAT5	0.841171889	0.000254123
219025_at	CD248 molecule, endosialin	CD248	-1.116191982	0.00028425
214180_at	mannosidase, alpha, class 1C, member 1	MAN1C1	-0.454728243	0.000295329
212562_s_at	Cathepsin Z precursor (CTSZ)	CTSZ	0.385954838	0.000301618
204484_at	phosphoinositide-3-kinase, class 2, beta polypeptide	PIK3C2B	-0.371678816	0.000339617
213435_at	SATB homeobox 2	SATB2	0.66594766	0.000365439
221583_s_at	potassium large conductance calcium-activated channel, subfamily M, alpha member 1	KCNMA1	0.720391303	0.000372464
211528_x_at	major histocompatibility complex, class I, G	HLA-G	0.260269689	0.000389438
210607_at	fms-related tyrosine kinase 3 ligand	FLT3LG	-0.432410714	0.000397135
221648_s_at	not yet annotated	NA	-0.332140054	0.000408087
205594_at	zinc finger protein 652	ZNF652	-0.498121115	0.000419955
211529_x_at	major histocompatibility complex, class I, G	HLA-G	0.285387853	0.00046583
211689_s_at	transmembrane protease, serine 2	TMPRSS2	0.82754955	0.000488075
205530_at	electron-transferring-flavoprotein dehydrogenase	ETFDH	0.245452971	0.000493292
201013_s_at	phosphoribosylaminoimidazole carboxylase, phosphoribosylaminoimidazole succinocarboxamide synthetase	PAICS	-0.20799853	0.000498347
210847_x_at	tumor necrosis factor receptor superfamily, member 25	TNFRSF25	-0.333481749	0.000503277
220650_s_at	solute carrier family 9 (sodium/hydrogen exchanger), member 5	SLC9A5	-0.717043099	0.000525664
203548_s_at	lipoprotein lipase	LPL	0.695863327	0.000616339
214739_at	leucine-rich repeats and calponin homology (CH) domain containing 3	LRCH3	-0.232108596	0.000625629
212996_s_at	URB1 ribosome biogenesis 1 homolog (S. cerevisiae)	URB1	-0.312788713	0.000625819
212659_s_at	interleukin 1 receptor antagonist	IL1RN	0.48822024	0.000721395
208250_s_at	deleted in malignant brain tumors 1	DMBT1	0.641287515	0.000736787
208423_s_at	macrophage scavenger receptor 1	MSR1	0.517802645	0.000761855
215295_at	dystrobrevin, beta	DTNB	-0.849038676	0.000778437
207990_x_at	acrosomal vesicle protein 1	ACRV1	0.625356194	0.000853422
213666_at	septin 6	SEPT6	-0.275134841	0.000853423
201340_s_at	ectodermal-neural cortex (with BTB-like domain)	ENC1	0.626294754	0.000877435
217647_at	not yet annotated	NA	-0.439387628	0.000944701
215049_x_at	CD163 molecule	CD163	0.279963425	0.000956214
215726_s_at	cytochrome b5 type A (microsomal)	CYB5A	-0.266627132	0.000976392
215750_at	KIAA1659 protein	KIAA1659	-0.397790885	0.000985982

202052_s_at	retinoic acid induced 14	RAI14	-0.90848263	0.000991565
214468_at	myosin, heavy chain 6, cardiac muscle, alpha	MYH6	0.778105799	0.001036691
202689_at	RNA binding motif protein 15B	RBM15B	0.162810206	0.001039642
217152_at	not yet annotated	NA	-0.256231878	0.001052851
221146_at	not yet annotated	NA	-0.544325116	0.001055778
202617_s_at	methyl CpG binding protein 2 (Rett syndrome)	MECP2	0.165387492	0.001082264
220609_at	hypothetical protein LOC202181	LOC202181	-0.255993832	0.001110672
218693_at	tetraspanin 15	TSPAN15	-0.48276688	0.00111286
210318_at	retinol binding protein 3, interstitial	RBP3	-0.833439145	0.001123025
204027_s_at	methyltransferase like 1	METTL1	-0.276513299	0.001125912
219496_at	ankyrin repeat domain 57	ANKRD57	0.669761984	0.001132405
207339_s_at	lymphotoxin beta (TNF superfamily, member 3)	LTB	-0.291010536	0.001148496
203788_s_at	sema domain, immunoglobulin domain (Ig), short basic domain, secreted, (semaphorin) 3C	SEMA3C	0.396970511	0.001189937
218676_s_at	phosphatidylcholine transfer protein	PCTP	0.23107584	0.001200463
219671_at	hippocalcin like 4	HPCAL4	-0.450659118	0.001216453
201203_s_at	ribosome binding protein 1 homolog 180kDa (dog)	RRBP1	1.128271592	0.001238956
206618_at	interleukin 18 receptor 1	IL18R1	0.438266711	0.001285442
203630_s_at	component of oligomeric golgi complex 5	COG5	-0.323793923	0.001287931
216227_at	HLA complex group 2 pseudogene 7	HCG2P7	0.597749177	0.00130013
33494_at	electron-transferring-flavoprotein dehydrogenase	ETFDH	0.206783093	0.001313642
218754_at	nucleolar protein 9	NOL9	-0.197077728	0.001319467
205491_s_at	gap junction protein, beta 3, 31kDa	GJB3	0.732235161	0.001325795
218273_s_at	protein phosphatase 2C, magnesium-dependent, catalytic subunit	PPM2C	0.304076084	0.00134614
210475_at	POU class 3 homeobox 1	POU3F1	-0.684792024	0.001380041
218675_at	solute carrier family 22, member 17	SLC22A17	-1.062766292	0.001385286
206260_at	transglutaminase 4 (prostate)	TGM4	-0.609864058	0.001394993
218899_s_at	brain and acute leukemia, cytoplasmic	BAALC	-0.481385196	0.001423021
202658_at	peroxisomal biogenesis factor 11 beta	PEX11B	-0.165482477	0.001430496
214500_at	H2A histone family, member Y	H2AFY	0.312225257	0.00143456
212186_at	acetyl-Coenzyme A carboxylase alpha	ACACA	-0.216541587	0.001462758
206674_at	fms-related tyrosine kinase 3	FLT3	0.354349815	0.001493866
216945_x_at	PAS domain containing serine/threonine kinase	PASK	-0.337753651	0.001511612
214661_s_at	NOP14 nucleolar protein homolog (yeast)	NOP14	-0.229819539	0.001565629

39705_at	SIN3 homolog B, transcription regulator (yeast)	SIN3B	-0.262877794	0.001580746
216964_at	Ubiquitin specific peptidase 22, mRNA (cDNA clone IMAGE:3629270)	USP22	-0.378087818	0.001607975
217450_at	similar to insulin receptor substrate like protein	LOC442715	0.694830012	0.001630985
216531_at	YY2 transcription factor	YY2	0.354964955	0.001705508
213159_at	pecanex homolog (Drosophila)	PCNX	0.225181822	0.001718209
215349_at	HCG1730474, mRNA (cDNA clone MGC:189748 IMAGE:9057072)	hCG_1730474	-0.315653202	0.001726704
211310_at	enhancer of zeste homolog 1 (Drosophila)	EZH1	-0.344283389	0.001732969
221649_s_at	peter pan homolog (Drosophila)	PPAN	-0.292142942	0.00175623
211591_s_at	phosphodiesterase 4A, cAMP-specific (phosphodiesterase E2 dunce homolog, Drosophila)	PDE4A	-0.754059002	0.001768511
217168_s_at	homocysteine-inducible, endoplasmic reticulum stress-inducible, ubiquitin-like domain member 1	HERPUD1	0.142395055	0.00178268
202643_s_at	tumor necrosis factor, alpha-induced protein 3	TNFAIP3	0.214012268	0.001796675
215646_s_at	versican	VCAN	0.427562571	0.001805152
205129_at	nucleophosmin/nucleoplasmin, 3	NPM3	-0.2679823	0.001891157
214997_at	CDNA clone IMAGE:5285458	GOLGA1	-0.339527541	0.001901639
31637_s_at	nuclear receptor subfamily 1, group D, member 1 /// thyroid hormone receptor, alpha (erythroblastic leukemia viral (v-erb-a) oncogene homolog, avian)	NR1D1 /// THRA	-0.368157253	0.001969414
220652_at	kinesin family member 24	KIF24	0.530692715	0.00202485
212831_at	multiple EGF-like-domains 9	MEGF9	0.26530666	0.002032823
217645_at	COX16 cytochrome c oxidase assembly homolog (S. cerevisiae)	COX16	-0.300039031	0.002045937
219882_at	tubulin tyrosine ligase-like family, member 7	TTLL7	0.656157914	0.002062414
208241_at	neuregulin 1	NRG1	0.803389569	0.002129758
216624_s_at	myeloid/lymphoid or mixed-lineage leukemia (trithorax homolog, Drosophila)	MLL	0.774520202	0.002164031
208978_at	cysteine-rich protein 2	CRIP2	-0.938311947	0.002183632
200965_s_at	actin binding LIM protein 1	ABLIM1	-0.224575067	0.002250916
208895_s_at	DEAD (Asp-Glu-Ala-Asp) box polypeptide 18	DDX18	-0.204418024	0.002287737
204555_s_at	protein phosphatase 1, regulatory (inhibitor) subunit 3D	PPP1R3D	0.424983905	0.002346258
212162_at	kinase D-interacting substrate, 220kDa	KIDINS220	0.232692954	0.002391666
214103_s_at	RAP2A, member of RAS oncogene family, mRNA (cDNA clone MGC:41795 IMAGE:5278625)	RAP2A	0.600783638	0.002409283
203549_s_at	lipoprotein lipase	LPL	0.705629582	0.002462286
201221_s_at	small nuclear ribonucleoprotein 70kDa (U1)	SNRNP70	-0.3146288	0.002473321

221968_s_at	Zinc finger protein 771 (ZNF771), transcript variant 2, mRNA	ZNF771	-0.933094787	0.002504067
220295_x_at	DEP domain containing 1	DEPDC1	0.354859202	0.002518822
203825_at	bromodomain containing 3	BRD3	-0.278761111	0.002575541
201677_at	Clone 23837 mRNA sequence	C3orf37	-0.175528882	0.002650468
201396_s_at	small glutamine-rich tetratricopeptide repeat (TPR)-containing, alpha	SGTA	-0.293906954	0.002664609
220068_at	pre-B lymphocyte 3	VPREB3	-0.468239933	0.002679446
213963_s_at	Sin3A-associated protein, 30kDa, mRNA (cDNA clone MGC:13685 IMAGE:4074154)	SAP30	0.358965223	0.002763904
220818_s_at	transient receptor potential cation channel, subfamily C, member 4	TRPC4	0.660067978	0.002783459
213534_s_at	PAS domain containing serine/threonine kinase	PASK	-0.357270414	0.002789842
218670_at	pseudouridylate synthase 1	PUS1	-0.302994779	0.002821509
207445_s_at	chemokine (C-C motif) receptor 9	CCR9	-0.485330289	0.002846486
207995_s_at	C-type lectin domain family 4, member M	CLEC4M	0.591580309	0.002930408
202864_s_at	SP100 nuclear antigen	SP100	0.186197954	0.002966928
220354_at	similar to hCG1774568	hCG_1774568	-0.572523498	0.002997815
222348_at	KIAA0303 gene	MAST4	-0.27403997	0.003000198
214309_s_at	not yet annotated	NA	-0.34054797	0.003023758
212400_at	family with sequence similarity 102, member A	FAM102A	-0.265637996	0.003038778
206428_s_at	zinc finger protein 143	ZNF143	0.610233111	0.003051507
202435_s_at	cytochrome P450, family 1, subfamily B, polypeptide 1	CYP1B1	0.425163769	0.003173176
207103_at	potassium voltage-gated channel, Shal-related subfamily, member 2	KCND2	0.512669239	0.003218315
217275_at	testis-specific serine kinase 2	TSSK2	0.76170441	0.003252926
216219_at	aquaporin 6, kidney specific	AQP6	0.416107628	0.003273055
207190_at	zinc finger, ZZ-type with EF-hand domain 1	ZZEF1	0.169781547	0.003294357
219315_s_at	transmembrane protein 204	TMEM204	-0.339640135	0.003303077
212142_at	minichromosome maintenance complex component 4	MCM4	0.946242543	0.003305126
204610_s_at	coiled-coil domain containing 85B	CCDC85B	-0.325985406	0.003305389
209197_at	synaptotagmin XI	SYT11	0.206494795	0.003374913
211715_s_at	3-hydroxybutyrate dehydrogenase, type 1	BDH1	-0.281854344	0.003378891
211648_at	immunoglobulin heavy constant alpha 1 /// immunoglobulin heavy constant gamma 1 (G1m marker) /// similar to hCG1773549	IGHA1 /// IGHG1 /// LOC100133862	-0.530825606	0.003379971
203996_s_at	chromosome 21 open reading frame 2	C21orf2	-0.286726183	0.003423145

210042_s_at	cathepsin Z	CTSZ	0.279771021	0.003444137
210251_s_at	RUN and FYVE domain containing 3	RUFY3	0.201019286	0.003459629
64942_at	G protein-coupled receptor 153	GPR153	0.266278797	0.003479779
211680_at	PDZ and LIM domain 5	PDLIM5	-0.396531642	0.003498029
213804_at	inositol polyphosphate-5-phosphatase, 75kDa	INPP5B	-0.336355767	0.003499911
219423_x_at	tumor necrosis factor receptor superfamily, member 25	TNFRSF25	-0.268879203	0.003584038
213674_x_at	immunoglobulin heavy constant delta	IGHD	-0.533605489	0.003584432
220383_at	ATP-binding cassette, sub-family G (WHITE), member 5	ABCG5	0.635660741	0.003756643
206226_at	histidine-rich glycoprotein	HRG	0.626045518	0.003794371
215346_at	CD40 molecule, TNF receptor superfamily member 5	CD40	-0.28989747	0.003884121
201041_s_at	dual specificity phosphatase 1	DUSP1	0.220084077	0.003955571
206100_at	carboxypeptidase M	CPM	0.345007902	0.003965472
219803_at	angiopoietin-like 3	ANGPTL3	0.543288722	0.004076861
204527_at	myosin VA (heavy chain 12, myoxin)	MYO5A	0.201004588	0.004103067
209558_s_at	huntingtin interacting protein 1 related	HIP1R	-0.372127988	0.004112271
204581_at	CD22 molecule	CD22	-0.419367055	0.004132178
220215_at	zinc finger protein 669	ZNF669	-0.183876496	0.004157883
208620_at	poly(rC) binding protein 1	PCBP1	0.161605136	0.004160509
206806_at	diacylglycerol kinase, iota	DGKI	0.510941137	0.004232878
220843_s_at	WD repeats and SOF1 domain containing	WDSOF1	0.323285245	0.004237266
212794_s_at	KIAA1033	KIAA1033	0.1395113	0.004238224
207468_s_at	secreted frizzled-related protein 5	SFRP5	-0.873636533	0.004315849
207630_s_at	cAMP responsive element modulator	CREM	0.258012321	0.004343762
207078_at	mediator complex subunit 6	MED6	-0.261673531	0.004404355
208537_at	sphingosine-1-phosphate receptor 2	S1PR2	0.662172135	0.004500845
208317_at	xylulokinase homolog (H. influenzae)	XYLB	-0.473168793	0.004514817
217466_x_at	ribosomal protein S2	RPS2	-0.207198853	0.004517758
217170_at	not yet annotated	NA	-0.402411624	0.004520679
216723_at	not yet annotated	NA	0.58776772	0.004571784
213639_s_at	zinc finger protein 500	ZNF500	-0.189088168	0.004608711
222323_at	not yet annotated	NA	0.596033631	0.004639537
212357_at	family with sequence similarity 168, member A	FAM168A	0.191133998	0.004661274
209789_at	coronin, actin binding protein, 2B	CORO2B	-0.695919622	0.004741575
211914_x_at	neurofibromin 1	NF1	0.321772784	0.004743772
209871_s_at	amyloid beta (A4)precursor protein-binding, family A,member2	APBA2	-0.349285391	0.00477428

218991_at	HEAT repeat containing 6	HEATR6	0.19490177	0.004788041
215390_at	not yet annotated	NA	-0.541438574	0.004788452
212053_at	pyridoxal-dependent decarboxylase domain containing 1	PDXDC1	0.113427359	0.004833681
222027_at	CDNA clone IMAGE:4513453	NUCKS1	-0.254979345	0.004939806
205254_x_at	transcription factor 7 (T-cell specific, HMG-box)	TCF7	-0.289171917	0.004978627
206307_s_at	forkhead box D1	FOXD1	0.572579113	0.005056677
222048_at	crystallin, beta B2 pseudogene 1	CRYBB2P1	-0.253002717	0.005145509
215323_at	leucine zipper protein 2	LUZP2	0.591641403	0.005166825
208903_at	ribosomal protein S28	RPS28	-0.194196541	0.005182963
219846_at	gon-4-like (C. elegans)	GON4L	-0.360815723	0.005206435
212715_s_at	microtubule associated monoxygenase, calponin and LIM domain containing 3	MICAL3	-0.239217707	0.005253409
206413_s_at	T-cell leukemia/lymphoma 1B /// T-cell leukemia/lymphoma 6	TCL1B /// TCL6	-0.550765999	0.005254097
222031_at	hypothetical protein LOC286434 /// similar to Serine/threonine-protein kinase PRKX (Protein kinase PKX1) /// hypothetical protein LOC441528 /// similar to hCG1981372 /// similar to LOC389906 protein	LOC286434 /// LOC389906 /// LOC441528 /// LOC728687 /// LOC729162	-0.318611865	0.005435773
213834_at	IQ motif and Sec7 domain 3 /// similar to IQ motif and Sec7 domain-containing protein 3 /// similar to IQ motif and Sec7 domain 3	IQSEC3 /// LOC100134209 /// LOC731035	-0.735086065	0.005501547
220349_s_at	endo-beta-N-acetylglucosaminidase	FLJ21865	-0.223275359	0.005546515
220618_s_at	zinc finger, CW type with PWWP domain 1	ZCWPW1	-0.209039313	0.00560885
211571_s_at	versican	VCAN	0.381173514	0.005617021
204762_s_at	guanine nucleotide binding protein (G protein), alpha activating activity polypeptide O	GNAO1	0.285308185	0.005641332
213335_s_at	ST3 beta-galactoside alpha-2,3-sialyltransferase 6	ST3GAL6	0.247035772	0.005658616
206427_s_at	melan-A	MLANA	0.27287048	0.005659764
206074_s_at	high mobility group AT-hook 1	HMGA1	-0.230716771	0.005667789
212935_at	MCF.2 cell line derived transforming sequence-like	MCF2L	-0.399163398	0.005743264
221101_at	chromosome 14 open reading frame 113	C14orf113	-0.628612237	0.005756916
214083_at	hypothetical protein LOC100132532	LOC100132532	-0.180920239	0.005767622
209245_s_at	kinesin family member 1C	KIF1C	-0.542430857	0.005885563
222141_at	kelch-like 22 (Drosophila)	KLHL22	-0.229908614	0.005904523
210514_x_at	major histocompatibility complex, class I, G	HLA-G	0.232489798	0.005993239
212210_at	integrator complex subunit 1	INTS1	0.737172414	0.005993643

206761_at	CD96 molecule	CD96	-0.317799185	0.005999151
205789_at	CD1d molecule	CD1D	0.214021289	0.006000346
210350_x_at	inhibitor of growth family, member 1	ING1	0.35863245	0.006010631
207274_at	cholinergic receptor, nicotinic, epsilon	CHRNE	0.291246084	0.006023634
203716_s_at	dipeptidyl-peptidase 4	DPP4	-0.228421293	0.006060235
217539_at	chromosome 18 open reading frame 25	C18orf25	-0.326483672	0.006061989
214217_at	glutamate receptor, metabotropic 5	GRM5	-0.614134196	0.006063128
220085_at	helicase, lymphoid-specific	HELLS	-0.34642368	0.006100818
214875_x_at	amyloid beta (A4) precursor-like protein 2	APLP2	0.227399027	0.006115735
203127_s_at	serine palmitoyltransferase, long chain base subunit 2	SPTLC2	0.217472996	0.006171437
221261_x_at	melanoma antigen family D, 4 /// melanoma antigen family D, 4B	MAGED4 /// MAGED4B	-0.322204558	0.006287221
216997_x_at	transducin-like enhancer of split 4 (E(sp1) homolog, Drosophila)	TLE4	0.28446533	0.006431176
218900_at	cyclin M4	CNNM4	0.158079757	0.006591928
212414_s_at	cytokine-like nuclear factor n-pac /// septin 6	N-PAC /// SEPT6	-0.189889638	0.00659997
220384_at	thioredoxin domain containing 3 (spermatozoa)	TXNDC3	0.30466639	0.006610729
221272_s_at	chromosome 1 open reading frame 21	C1orf21	0.294817026	0.00662324
201206_s_at	ribosome binding protein 1 homolog 180kDa (dog)	RRBP1	0.466774857	0.006651951
218295_s_at	nucleoporin 50kDa	NUP50	0.193682438	0.006739229
210993_s_at	SMAD family member 1	SMAD1	0.242243261	0.006756001
41113_at	zinc finger protein 500	ZNF500	-0.206899358	0.006780245
217644_s_at	son of sevenless homolog 2 (Drosophila)	SOS2	0.20192813	0.006787288
221646_s_at	zinc finger, DHHC-type containing 11	ZDHHC11	-0.366155397	0.007089167
37005_at	neuroblastoma, suppression of tumorigenicity 1	NBL1	-0.320804176	0.007092559
204589_at	NUAK family, SNF1-like kinase, 1	NUAK1	0.518721613	0.007100007
213994_s_at	spondin 1, extracellular matrix protein	SPON1	-0.305667183	0.007132784
221979_at	similar to hCG1811779	LOC100129250	-0.37915977	0.007191765
219516_at	transient receptor potential cation channel, subfamily V, member 4	TRPV4	0.733375569	0.007227062
200827_at	procollagen-lysine 1, 2-oxoglutarate 5-dioxygenase 1	PLOD1	0.267136643	0.00734353
210675_s_at	protein tyrosine phosphatase, receptor type, R	PTPRR	0.455559093	0.007394684
35147_at	MCF.2 cell line derived transforming sequence-like	MCF2L	-0.311114432	0.007411365
222285_at	immunoglobulin heavy constant delta	IGHD	-0.477588472	0.007422004
203226_s_at	tetraspanin 31	TSPAN31	-0.158389563	0.007424552
209841_s_at	leucine rich repeat neuronal 3	LRRN3	-0.531186942	0.007449328

205267_at	POU class 2 associating factor 1	POU2AF1	-0.265196778	0.007492213
212075_s_at	casein kinase 2, alpha 1 polypeptide	CSNK2A1	0.269084816	0.007665417
205376_at	inositol polyphosphate-4-phosphatase, type II, 105kDa	INPP4B	-0.229690301	0.007667837
211722_s_at	histone deacetylase 6	HDAC6	0.423101669	0.007677094
201430_s_at	dihydropyrimidinase-like 3	DPYSL3	0.41043335	0.007683671
203215_s_at	myosin VI	MYO6	0.356675766	0.007699807
207464_at	S-adenosylhomocysteine hydrolase-like 1	AHCYL1	-0.289943667	0.00776576
212617_at	zinc finger protein 609	ZNF609	-0.212939688	0.007767226
201934_at	WD repeat domain 82	WDR82	-0.12685142	0.007916491
204679_at	potassium channel, subfamily K, member 1	KCNK1	0.354192865	0.008005695
217492_s_at	phosphatase and tensin homolog /// phosphatase and tensin homolog pseudogene 1	PTEN /// PTENP1	0.17004751	0.008034531
219971_at	interleukin 21 receptor	IL21R	-0.220840628	0.008036557
219221_at	zinc finger and BTB domain containing 38	ZBTB38	0.274887587	0.008085445
217207_s_at	butyrophilin-like 3	BTNL3	2.576512829	0.00813886
212374_at	fem-1 homolog b (C. elegans)	FEM1B	0.23284695	0.008189826
221907_at	tRNA methyltransferase 61 homolog A (S. cerevisiae)	TRMT61A	-0.405044177	0.008245226
214076_at	not yet annotated	NA	0.16899714	0.008249382
214139_at	not yet annotated	NA	0.256206733	0.008344832
39248_at	aquaporin 3 (Gill blood group)	AQP3	-0.171771672	0.008376573
206786_at	histatin 3	HTN3	-0.502825959	0.008462647
221088_s_at	protein phosphatase 1, regulatory (inhibitor) subunit 9A	PPP1R9A	0.517078015	0.008467344
220799_at	glial cells missing homolog 2 (Drosophila)	GCM2	0.325044024	0.008484862
219068_x_at	ATPase family, AAA domain containing 3A /// ATPase family, AAA domain containing 3C	ATAD3A /// ATAD3C	-0.331060383	0.008555447
206190_at	G protein-coupled receptor 17	GPR17	-0.622058564	0.008578201
206257_at	coiled-coil domain containing 9	CCDC9	-0.32808322	0.008590667
211462_s_at	transducin (beta)-like 1Y-linked	TBL1Y	0.758743686	0.00862459
209485_s_at	oxysterol binding protein-like 1A	OSBPL1A	0.231186115	0.008638068
215485_s_at	intercellular adhesion molecule 1	ICAM1	0.246284716	0.008653038
215786_at	not yet annotated	NA	-0.331581149	0.008655853
AFFX-r2-Ec-bioB-M_at	not yet annotated	NA	0.202988566	0.008699215
221906_at	thioredoxin reductase 3	TXNRD3	-0.678453091	0.008700847
203545_at	asparagine-linked glycosylation 8, alpha-1,3-glucosyltransferase homolog (S. cerevisiae)	ALG8	-0.196360591	0.008816012

212101_at	karyopherin alpha 6 (importin alpha 7)	KPNA6	-0.149813243	0.008828397
205656_at	protocadherin 17	PCDH17	0.475701043	0.008836367
205587_at	FGFR1 oncogene partner	FGFR1OP	-0.361935252	0.008899023
216202_s_at	serine palmitoyltransferase, long chain base subunit 2	SPTLC2	0.254967789	0.008942523
216503_s_at	myeloid/lymphoid or mixed-lineage leukemia (trithorax homolog, Drosophila); translocated to, 10	MLLT10	0.308260764	0.008972445
219532_at	elongation of very long chain fatty acids (FEN1/Elo2, SUR4/Elo3, yeast)-like 4	ELOVL4	-0.492361483	0.008974219
204560_at	FK506 binding protein 5	FKBP5	0.358924107	0.009002514
220183_s_at	nudix (nucleoside diphosphate linked moiety X)-type motif 6	NUDT6	0.295527592	0.009014013
215743_at	N-myristoyltransferase 2	NMT2	-0.261777588	0.009039357
204553_x_at	inositol polyphosphate-4-phosphatase, type I, 107kDa	INPP4A	0.154705415	0.009229342
213580_at	not yet annotated	NA	0.341889864	0.009230048
209995_s_at	T-cell leukemia/lymphoma 1A	TCL1A	-0.459081297	0.009247181
221790_s_at	low density lipoprotein receptor adaptor protein 1	LDLRAP1	-0.238946582	0.009534002
210121_at	UDP-Gal:betaGlcNAc beta 1,3-galactosyltransferase, polypeptide 2	B3GALT2	-0.434610925	0.009545439
212413_at	septin 6	SEPT6	-0.184567146	0.00954606
214968_at	DEAD (Asp-Glu-Ala-Asp) box polypeptide 51	DDX51	-0.555371762	0.00962018
201995_at	exostoses (multiple) 1	EXT1	0.206942896	0.00965575
214216_s_at	La ribonucleoprotein domain family, member 5	LARP5	0.592120916	0.009690984
212766_s_at	interferon stimulated exonuclease gene 20kDa-like 2	ISG20L2	-0.138246408	0.009785639
208806_at	chromodomain helicase DNA binding protein 3	CHD3	-0.203416965	0.009796176
217995_at	sulfide quinone reductase-like (yeast)	SQRDL	0.165335372	0.009812184
216751_at	not yet annotated	NA	-0.244495939	0.009815445
212542_s_at	pleckstrin homology domain interacting protein	PHIP	0.152145075	0.009876101
212763_at	calmodulin regulated spectrin-associated protein 1-like 1	CAMSAP1L1	0.297966492	0.009883783
219124_at	chromosome 8 open reading frame 41	C8orf41	-0.085951827	0.009889273
206320_s_at	SMAD family member 9	SMAD9	-0.393509714	0.009900105
213340_s_at	KIAA0495	KIAA0495	-0.147360363	0.009936118
214563_at	protocadherin gamma subfamily C, 3	PCDHGC3	0.549977929	0.009950263
203450_at	chibby homolog 1 (Drosophila)	CBY1	-0.136339732	0.009952094
