# Supplementary material for: Blood transcriptomics of drug-naïve sporadic Parkinson’s disease patients
Source: BMC Genomics. 2015 Oct 28;16:876. doi: 10.1186/s12864-015-2058-3 (PMC4625854; doi:10.1186/s12864-015-2058-3)
Supplement: Additional file 7: — List of differentially expressed genes between PD patients and controls based on Significance Analysis of Microarrays (SAM). Significance Analysis of Microarrays was performed on normalized and filtered microarray data as described in the main text. The SAM test was run using 1000 permutations and a False Discovery Rate (FDR) of 10 %. Ratios are calculated as differences in gene expression between samples of PD patients and controls, with the top up-regulated genes first. (PDF 3122 kb) [file 12864_2015_2058_MOESM7_ESM.pdf]

Additional file 7. List of differentially expressed genes between PD patients and controls based on Significance Analysis of Microarrays (SAM). Significance Analysis of Microarrays was performed on normalized and filtered microarray data as described in the main text. The SAM test was run using 1000 permutations and a False Discovery Rate (FDR) of 10%. Ratios are calculated as differences in gene expression between samples of PD patients and controls, with the top up-regulated genes first.

Affymetrix probe ID	Gene name	Gene symbol	Fold change	Ratio
215390_at	chromosome 9 open reading frame 5	C9orf5	0.61	-1.65
217671_at	regulatory factor X, 3 (influences HLA class II expression)	RFX3	0.63	-1.59
205594_at	zinc finger protein 652	ZNF652	0.64	-1.57
219532_at	elongation of very long chain fatty acids (FEN1/Elo2, SUR4/Elo3, yeast)-like 4	ELOVL4	0.65	-1.54
215750_at	KIAA1659 protein	KIAA1659	0.66	-1.53
209841_s_at	leucine rich repeat neuronal 3	LRRN3	0.66	-1.52
209789_at	coronin, actin binding protein, 2B	CORO2B	0.66	-1.52
219671_at	hippocalcin like 4	HPCAL4	0.66	-1.51
213674_x_at	immunoglobulin heavy constant delta	IGHD	0.67	-1.50
221101_at	chromosome 14 open reading frame 113	C14orf113	0.67	-1.50
205674_x_at	FXYD domain containing ion transport regulator 2	FXYD2	0.67	-1.49
220068_at	pre-B lymphocyte gene 3	VPREB3	0.69	-1.46
210701_at	craniofacial development protein 1	CFDP1	0.69	-1.46
222285_at	immunoglobulin heavy constant delta	IGHD	0.7	-1.44
217647_at	dihydroorotate dehydrogenase	DHODH	0.7	-1.43
209871_s_at	amyloid beta (A4) precursor protein-binding, family A, member 2 (X11-like)	APBA2	0.7	-1.42
214180_at	mannosidase, alpha, class 1C, member 1	MAN1C1	0.72	-1.39
215786_at	remodeling and spacing factor 1	RSF1	0.72	-1.39
219846_at	gon-4-like (C. elegans)	GON4L	0.72	-1.39
31637_s_at	thyroid hormone receptor, alpha (erythroblastic leukemia viral (v-erb-a) oncogene homolog, avian)	THRA	0.72	-1.39
214997_at	Golgi autoantigen, golgin subfamily a, 1	GOLGA1	0.73	-1.38
218693_at	tetraspanin 15	TSPAN15	0.73	-1.36
219315_s_at	chromosome 16 open reading frame 30	C16orf30	0.74	-1.36
209715_at	chromobox homolog 5 (HP1 alpha homolog, Drosophila)	CBX5	0.74	-1.35
220085_at	helicase, lymphoid-specific	HELLS	0.74	-1.35
33307_at	ribosomal RNA processing 7 homolog A (S. cerevisiae)	RRP7A	0.74	-1.34
213922_at	tau tubulin kinase 2	TTBK2	0.74	-1.34

206548_at	hypothetical protein FLJ23556	FLJ23556	0.75	-1.34
210607_at	fms-related tyrosine kinase 3 ligand	FLT3LG	0.75	-1.34
213804_at	inositol polyphosphate-5-phosphatase, 75kDa	INPP5B	0.75	-1.34
209153_s_at	transcription factor 3 (E2A immunoglobulin enhancer binding factors E12/E47)	TCF3	0.75	-1.33
214309_s_at	Chromosome 21 open reading frame 2	C21orf2	0.75	-1.33
213462_at	neuronal PAS domain protein 2	NPAS2	0.76	-1.32
210450_at	hypothetical protein LOC90925	LOC90925	0.76	-1.32
218906_x_at	kinesin light chain 2	KLC2	0.76	-1.32
222348_at	Microtubule associated serine/threonine kinase family member 4	MAST4	0.76	-1.31
212935_at	MCF.2 cell line derived transforming sequence-like	MCF2L	0.77	-1.30
213428_s_at	collagen, type VI, alpha 1	COL6A1	0.77	-1.30
222337_at	oxysterol binding protein-like 9	OSBPL9	0.77	-1.30
219025_at	CD248 molecule, endosialin	CD248	0.77	-1.30
219815_at	galactose-3-O-sulfotransferase 4	GAL3ST4	0.77	-1.29
211310_at	enhancer of zeste homolog 1 (Drosophila)	EZH1	0.78	-1.29
210847_x_at	tumor necrosis factor receptor superfamily, member 25	TNFRSF25	0.78	-1.29
215743_at	N-myristoyltransferase 2	NMT2	0.78	-1.29
212996_s_at	URB1 ribosome biogenesis 1 homolog (S. cerevisiae)	URB1	0.78	-1.28
35147_at	MCF.2 cell line derived transforming sequence-like	MCF2L	0.78	-1.28
221648_s_at	agmatine ureohydrolase (agmatinase)	AGMAT	0.78	-1.28
203630_s_at	component of oligomeric golgi complex 5	COG5	0.78	-1.28
221979_at	Topoisomerase I binding, arginine/serine-rich	TOPORS	0.78	-1.28
221923_s_at	nucleophosmin (nucleolar phosphoprotein B23, numatrin)	NPM1	0.78	-1.28
217645_at	chromosome 14 open reading frame 112	C14orf112	0.78	-1.28
220728_at	NA	NA	0.79	-1.27
207078_at	mediator complex subunit 6	MED6	0.79	-1.27
222091_at	hippocalcin like 4	HPCAL4	0.79	-1.27
206761_at	CD96 molecule	CD96	0.79	-1.27
222027_at	Nuclear casein kinase and cyclin-dependent kinase substrate 1	NUCKS1	0.79	-1.27
203996_s_at	chromosome 21 open reading frame 2	C21orf2	0.79	-1.26
213283_s_at	sal-like 2 (Drosophila)	SALL2	0.79	-1.26
221963_x_at	Transcribed locus	NA	0.79	-1.26
215151_at	dedicator of cytokinesis 10	DOCK10	0.79	-1.26
207339_s_at	lymphotoxin beta (TNF superfamily, member 3)	LTB	0.79	-1.26
204484_at	phosphoinositide-3-kinase, class 2, beta polypeptide	PIK3C2B	0.8	-1.26

219423_x_at	tumor necrosis factor receptor superfamily, member 25	TNFRSF25	0.8	-1.25
214739_at	leucine-rich repeats and calponin homology (CH) domain containing 3	LRCH3	0.8	-1.25
217152_at	hypothetical LOC100271832	LOC100271832	0.81	-1.24
213666_at	septin 6	SEPT6	0.81	-1.24
210047_at	solute carrier family 11 (proton-coupled divalent metal ion transporters), member 2	SLC11A2	0.81	-1.24
211282_x_at	tumor necrosis factor receptor superfamily, member 25	TNFRSF25	0.81	-1.24
203716_s_at	dipeptidyl-peptidase 4 (CD26, adenosine deaminase complexing protein 2)	DPP4	0.81	-1.24
220609_at	hypothetical protein LOC202181	LOC202181	0.81	-1.24
203825_at	bromodomain containing 3	BRD3	0.81	-1.23
206413_s_at	T-cell leukemia/lymphoma 6	TCL6	0.81	-1.23
215726_s_at	cytochrome b5 type A (microsomal)	CYB5A	0.82	-1.22
215346_at	CD40 molecule, TNF receptor superfamily member 5	CD40	0.82	-1.22
219632_s_at	transient receptor potential cation channel, subfamily V, member 1	TRPV1	0.82	-1.22
222141_at	kelch-like 22 (Drosophila)	KLHL22	0.82	-1.21
37590_g_at	MRNA full length insert cDNA clone EUROIMAGE 375854///MRNA full length insert cDNA clone	NA	0.83	-1.21
201013_s_at	phosphoribosylaminoimidazole carboxylase, phosphoribosylaminoimidazole succinocarboxamide	PAICS	0.83	-1.21
220215_at	zinc finger protein 669	ZNF669	0.83	-1.20
214775_at	Nedd4 binding protein 3	N4BP3	0.83	-1.20
207826_s_at	inhibitor of DNA binding 3, dominant negative helix-loop-helix protein	ID3	0.83	-1.20
215030_at	G-rich RNA sequence binding factor 1	GRSF1	0.84	-1.20
214661_s_at	nucleolar protein 14	NOL14	0.84	-1.20
221868_at	poly(A) binding protein interacting protein 2B	PAIP2B	0.84	-1.19
212186_at	acetyl-Coenzyme A carboxylase alpha	ACACA	0.84	-1.19
203226_s_at	tetraspanin 31	TSPAN31	0.84	-1.19
212414_s_at	septin 6	SEPT6	0.84	-1.19
220618_s_at	zinc finger, CW type with PWWP domain 1	ZCWPW1	0.85	-1.18
209623_at	methylcrotonoyl-Coenzyme A carboxylase 2 (beta)	MCCC2	0.85	-1.18
41113_at	zinc finger protein 500	ZNF500	0.85	-1.18
57516_at	zinc finger protein 764	ZNF764	0.85	-1.18
213340_s_at	KIAA0495	KIAA0495	0.87	-1.15
217807_s_at	glioma tumor suppressor candidate region gene 2	GLTSCR2	0.87	-1.15
39248_at	aquaporin 3 (Gill blood group)	AQP3	0.88	-1.14
208828_at	polymerase (DNA directed), epsilon 3 (p17 subunit)	POLE3	0.88	-1.14

202658_at	peroxisomal biogenesis factor 11B	PEX11B	0.88	-1.13
220152_at	chromosome 10 open reading frame 95	C10orf95	0.89	-1.12
208763_s_at	TSC22 domain family, member 3	TSC22D3	1.15	1.15
217995_at	sulfide quinone reductase-like (yeast)	SQRDL	1.17	1.17
205530_at	electron-transferring-flavoprotein dehydrogenase	ETFDH	1.22	1.22
203645_s_at	CD163 molecule	CD163	1.27	1.27
212831_at	multiple EGF-like-domains 9	MEGF9	1.29	1.29
204530_s_at	thymocyte selection-associated high mobility group box	TOX	1.29	1.29
203658_at	solute carrier family 25 (carnitine/acylcarnitine translocase), member 20	SLC25A20	1.29	1.29
221592_at	TBC1 domain family, member 8 (with GRAM domain)	TBC1D8	1.33	1.33
206757_at	phosphodiesterase 5A, cGMP-specific	PDE5A	1.34	1.34
213006_at	CCAAT/enhancer binding protein (C/EBP), delta	CEBPD	1.35	1.35
213963_s_at	Sin3A-associated protein, 30kDa	SAP30	1.35	1.35
208893_s_at	dual specificity phosphatase 6	DUSP6	1.37	1.37
206618_at	interleukin 18 receptor 1	IL18R1	1.45	1.45
202436_s_at	cytochrome P450, family 1, subfamily B, polypeptide 1	CYP1B1	1.48	1.48
201340_s_at	ectodermal-neural cortex (with BTB-like domain)	ENC1	1.55	1.55
213435_at	SATB homeobox 2	SATB2	1.67	1.67
217207_s_at	butyrophilin-like 3	BTNL3	2.11	2.11
