# Supplementary material for: Blood transcriptomics of drug-naïve sporadic Parkinson’s disease patients
Source: BMC Genomics. 2015 Oct 28;16:876. doi: 10.1186/s12864-015-2058-3 (PMC4625854; doi:10.1186/s12864-015-2058-3)
Supplement: Additional file 8: — List of differentially expressed genes between PD patients and controls based on Rank Product analysis (RP). Rank Product analysis was performed on normalized and filtered microarray data as described in the main text. The RP test was run using 100 permutations and an FDR of 0.005 % and looking for up- and down-regulated genes separately. Low RP-Values indicate high significance of the results. Genes are shown with the most significant first. (PDF 3122 kb) [file 12864_2015_2058_MOESM8_ESM.pdf]

Additional file 8. List of differentially expressed genes between PD patients and controls based on Rank Product analysis (RP). Rank Product analysis was performed on normalized and filtered microarray data as described in the main text. The RP test was run using 100 permutations and an FDR of 0·005%, and looking for up- and down-regulated genes separately. Low RP-Values indicate high significance of the results. Genes are shown with the most significant first.

Up-regulated genes

Affymetrix probe ID	Gene name	Gene symbol	p-Values	q-Values	RP-Values	Fold Change
217207_s_at	butyrophilin-like 3	BTNL3	0	0	206.35	0.33
213831_at	major histocompatibility complex, class II, DQ alpha 1	HLA-DQA1	0	0	337.25	0.37
209480_at	major histocompatibility complex, class II, DQ beta 1	HLA-DQB1	0	0	448.67	0.42
201909_at	ribosomal protein S4, Y-linked 1	RPS4Y1	0	0	497.16	0.48
214131_at	chromosome Y open reading frame 15B	CYorf15B	0	0	589.67	0.51
206700_s_at	jumonji, AT rich interactive domain 1D	JARID1D	0	0	591.58	0.56
205000_at	DEAD (Asp-Glu-Ala-Asp) box polypeptide 3, Y-linked	DDX3Y	0	0	623.18	0.56
204409_s_at	eukaryotic translation initiation factor 1A, Y-linked	EIF1AY	0	0	628.71	0.55
211149_at	ubiquitously transcribed tetratricopeptide repeat gene, Y-linked	UTY	0	0	747.74	0.61
204410_at	eukaryotic translation initiation factor 1A, Y-linked	EIF1AY	0	0	758.92	0.59
205001_s_at	DEAD (Asp-Glu-Ala-Asp) box polypeptide 3, Y-linked	DDX3Y	0	0	791.40	0.63
205048_s_at	phosphoserine phosphatase	PSPH	0	0	810.22	0.66
206624_at	ubiquitin specific peptidase 9, Y-linked (fat facets-like, Drosophila)	USP9Y	0	0	810.49	0.69
209875_s_at	secreted phosphoprotein 1	SPP1	0	0	864.82	0.65
205767_at	epiregulin	EREG	0	0	878.49	0.65
212999_x_at	similar to Major histocompatibility complex, class II, DR beta 4	LOC100133484	0	0	889.36	0.68
207067_s_at	histidine decarboxylase	HDC	0	0	912.69	0.73
218345_at	transmembrane protein 176A	TMEM176A	0	0	1050.71	0.88
206279_at	protein kinase, Y-linked	PRKY	0	0	1066.04	0.75
209728_at	major histocompatibility complex, class II, DR beta 4	HLA-DRB4	0	0	1081.46	1.12
207269_at	defensin, alpha 4, corticostatin	DEFA4	0	0	1083.74	0.76
210322_x_at	ubiquitously transcribed tetratricopeptide repeat gene, Y-linked	UTY	0	0	1128.62	0.76
203757_s_at	carcinoembryonic antigen-related cell adhesion molecule 6 (non-specific cross reacting antigen)	CEACAM6	0	0	1130.74	0.73
220532_s_at	transmembrane protein 176B	TMEM176B	0	0	1147.14	0.98

204589_at	NUAK family, SNF1-like kinase, 1	NUAK1	0	0	1193.96	0.66
214983_at	testis-specific transcript, Y-linked 15	TTTY15	0	0	1194.43	0.81
215838_at	leukocyte immunoglobulin-like receptor, subfamily A (with TM domain), member 5	LILRA5	0	0	1198.81	0.67
212187_x_at	prostaglandin D2 synthase 21kDa (brain)	PTGDS	0	0	1213.10	0.72
202203_s_at	autocrine motility factor receptor	AMFR	0	0	1258.70	0.82
218340_s_at	ubiquitin-like modifier activating enzyme 6	UBA6	0	0	1261.83	0.80
206108_s_at	splicing factor, arginine/serine-rich 6	SFRS6	0	0	1275.36	0.76
201340_s_at	ectodermal-neural cortex (with BTB-like domain)	ENC1	0	0	1290.26	0.67
202018_s_at	lactotransferrin	LTF	0	0	1296.26	0.79
208067_x_at	ubiquitously transcribed tetratricopeptide repeat gene, Y-linked	UTY	0	0	1303.99	0.72
202072_at	heterogeneous nuclear ribonucleoprotein L	HNRNPL	0	0	1316.01	0.71
205624_at	carboxypeptidase A3 (mast cell)	CPA3	0	0	1332.81	0.90
206177_s_at	arginase, liver	ARG1	0	0	1337.55	0.71
201110_s_at	thrombospondin 1	THBS1	0	0	1343.09	0.79
219935_at	ADAM metallopeptidase with thrombospondin type 1 motif, 5	ADAMTS5	0	0	1369.69	0.73
209710_at	GATA binding protein 2	GATA2	0	0	1374.00	0.84
212730_at	desmuslin	DMN	0	0	1405.40	0.72
207063_at	chromosome Y open reading frame 14	CYorf14	0	0	1413.33	0.96
201058_s_at	myosin, light chain 9, regulatory	MYL9	0	0	1413.64	0.81
211748_x_at	prostaglandin D2 synthase 21kDa (brain)	PTGDS	0	0	1416.62	0.75
212509_s_at	matrix-remodelling associated 7	MXRA7	0	0	1437.64	0.75
211657_at	carcinoembryonic antigen-related cell adhesion molecule 6 (non-specific cross reacting antigen)	CEACAM6	0	0	1449.78	0.88
206647_at	hemoglobin, zeta	HBZ	0	0	1460.52	0.86
220120_s_at	erythrocyte membrane protein band 4.1 like 4A	EPB41L4A	0	0	1475.54	0.70
212793_at	dishevelled associated activator of morphogenesis 2	DAAM2	0	0	1484.05	0.76
221428_s_at	transducin (beta)-like 1 X-linked receptor 1	TBL1XR1	0	0	1496.24	0.88
209992_at	6-phosphofructo-2-kinase/fructose-2,6-biphosphatase 2	PFKFB2	0	0	1538.70	0.71
203815_at	glutathione S-transferase theta 1	GSTT1	0	0	1542.10	0.84
203789_s_at	sema domain, immunoglobulin domain (Ig), short basic domain, secreted, (semaphorin) 3C	SEMA3C	0	0	1543.80	0.74
203498_at	regulator of calcineurin 2	RCAN2	0	0	1547.99	0.82
207723_s_at	killer cell lectin-like receptor subfamily C, member 3	KLRC3	0	0	1560.26	0.76
211663_x_at	prostaglandin D2 synthase 21kDa (brain)	PTGDS	0	0	1567.19	0.76
215574_at	not yet annotated	NA	0	0	1570.36	0.83

205900_at	keratin 1	KRT1	0	0	1588.94	0.94
207496_at	membrane-spanning 4-domains, subfamily A, member 2 (Fc fragment of IgE, high affinity I, receptor for; beta polypeptide)	MS4A2	0	0	1613.09	0.84
216361_s_at	MYST histone acetyltransferase (monocytic leukemia) 3	MYST3	0	0	1616.63	0.77
215037_s_at	BCL2-like 1	BCL2L1	0	0	1621.20	0.81
202286_s_at	tumor-associated calcium signal transducer 2	TACSTD2	0	0	1623.29	0.94
35201_at	heterogeneous nuclear ribonucleoprotein L	HNRNPL	0	0	1629.31	0.75
214218_s_at	X (inactive)-specific transcript (non-protein coding)	XIST	0	0	1629.93	2.43
221753_at	slingshot homolog 1 (Drosophila)	SSH1	0	0	1631.41	0.74
215666_at	major histocompatibility complex, class II, DR beta 4	HLA-DRB4	0	0	1632.70	1.12
206448_at	zinc finger protein 365	ZNF365	0	0	1639.00	0.76
209686_at	S100 calcium binding protein B	S100B	0	0	1650.43	1.10
207802_at	cysteine-rich secretory protein 3	CRISP3	0	0	1651.19	0.85
203184_at	fibrillin 2	FBN2	0	0	1654.79	0.74
212520_s_at	SWI/SNF related, matrix associated, actin dependent regulator of chromatin, subfamily a, member 4	SMARCA4	7.24E-07	1.41E-04	1661.47	0.82
211430_s_at	immunoglobulin heavy constant gamma 3 (G3m marker)	IGHG3	7.24E-07	1.41E-04	1687.47	0.92
213338_at	transmembrane protein 158	TMEM158	1.45E-06	2.74E-04	1697.86	0.83
201369_s_at	zinc finger protein 36, C3H type-like 2	ZFP36L2	1.45E-06	2.74E-04	1700.06	0.78
212768_s_at	olfactomedin 4	OLFM4	1.45E-06	2.74E-04	1708.52	1.04
209706_at	NK3 homeobox 1	NKX3-1	1.45E-06	2.74E-04	1710.06	1.03
203216_s_at	myosin VI	MYO6	1.45E-06	2.74E-04	1710.42	0.77
210896_s_at	aspartate beta-hydroxylase	ASPH	1.45E-06	2.74E-04	1718.91	0.74
203638_s_at	fibroblast growth factor receptor 2	FGFR2	1.45E-06	2.74E-04	1724.42	0.87
205653_at	cathepsin G	CTSG	1.45E-06	2.74E-04	1746.61	0.88
202437_s_at	cytochrome P450, family 1, subfamily B, polypeptide 1	CYP1B1	1.45E-06	2.74E-04	1749.68	0.75
210321_at	granzyme H (cathepsin G-like 2, protein h-CCPX)	GZMH	1.45E-06	2.74E-04	1752.65	0.73
203215_s_at	myosin VI	MYO6	1.45E-06	2.74E-04	1752.72	0.72
204560_at	FK506 binding protein 5	FKBP5	1.45E-06	2.74E-04	1753.97	0.71
205158_at	ribonuclease, RNase A family, 4	RNASE4	1.45E-06	2.74E-04	1764.98	0.71
211654_x_at	major histocompatibility complex, class II, DQ beta 1	HLA-DQB1	1.45E-06	2.74E-04	1769.43	0.75
205654_at	complement component 4 binding protein, alpha	C4BPA	1.45E-06	2.74E-04	1771.64	0.89
202435_s_at	cytochrome P450, family 1, subfamily B, polypeptide 1	CYP1B1	2.17E-06	3.41E-04	1784.70	0.71
220122_at	multiple C2 domains, transmembrane 1	MCTP1	2.17E-06	3.41E-04	1785.38	0.79
206881_s_at	leukocyte immunoglobulin-like receptor, subfamily A (without TM domain), member 3	LILRA3	2.90E-06	4.44E-04	1801.77	0.76
221728_x_at	X (inactive)-specific transcript (non-protein coding)	XIST	2.90E-06	4.44E-04	1807.00	2.35

202436_s_at	cytochrome P450, family 1, subfamily B, polypeptide 1	CYP1B1	2.90E-06	4.44E-04	1807.27	0.69
204148_s_at	POM (POM121 homolog, rat) and ZP3 fusion	POMZP3	2.90E-06	4.44E-04	1816.13	0.79
204415_at	interferon, alpha-inducible protein 6	IFI6	2.90E-06	4.44E-04	1819.15	0.77
214153_at	ELOVL family member 5, elongation of long chain fatty acids (FEN1/Elo2, SUR4/Elo3-like, yeast)	ELOVL5	2.90E-06	4.44E-04	1820.53	0.76
202770_s_at	cyclin G2	CCNG2	3.62E-06	5.21E-04	1825.73	0.84
213319_s_at	cold shock domain protein A	CSDA	3.62E-06	5.21E-04	1828.32	0.78
207357_s_at	UDP-N-acetyl-alpha-D-galactosamine:polypeptide N-acetylgalactosaminyltransferase 10 (GalNAc-T10)	GALNT10	4.35E-06	6.12E-04	1829.48	0.72
205040_at	orosomucoid 1	ORM1	4.35E-06	6.12E-04	1829.49	0.90
204066_s_at	centaurin, gamma 2	CENTG2	4.35E-06	6.12E-04	1832.92	0.77
206676_at	carcinoembryonic antigen-related cell adhesion molecule 8	CEACAM8	4.35E-06	6.12E-04	1842.80	0.92
205110_s_at	fibroblast growth factor 13	FGF13	5.07E-06	6.86E-04	1854.84	1.00
211571_s_at	versican	VCAN	6.52E-06	8.74E-04	1874.05	0.71
220220_at	leucine rich repeat containing 37, member A4 (pseudogene)	LRRC37A4	7.24E-06	9.62E-04	1891.15	0.78
213478_at	kazrin	RP1-21O18.1	7.97E-06	0.001047619	1899.06	0.78
209031_at	cell adhesion molecule 1	CADM1	8.69E-06	0.001132075	1902.89	0.79
210504_at	Kruppel-like factor 1 (erythroid)	KLF1	9.42E-06	0.001214953	1906.12	0.95
215646_s_at	versican	VCAN	9.42E-06	0.001214953	1907.47	0.72
201341_at	ectodermal-neural cortex (with BTB-like domain)	ENC1	9.42E-06	0.001214953	1909.57	0.78
202411_at	interferon, alpha-inducible protein 27	IFI27	1.01E-05	0.001272727	1919.41	0.82
204141_at	tubulin, beta 2A	TUBB2A	1.09E-05	0.001351351	1922.91	1.18
206665_s_at	BCL2-like 1	BCL2L1	1.16E-05	0.001428571	1923.85	0.80
220553_s_at	PRP39 pre-mRNA processing factor 39 homolog (S. cerevisiae)	PRPF39	1.23E-05	0.001504425	1932.99	0.84
206618_at	interleukin 18 receptor 1	IL18R1	1.30E-05	0.001578947	1934.84	0.70
207674_at	Fc fragment of IgA, receptor for	FCAR	1.30E-05	0.001578947	1936.56	1.11
205728_at	not yet annotated	NA	1.30E-05	0.001578947	1938.23	0.78
204713_s_at	coagulation factor V (proaccelerin, labile factor)	F5	1.30E-05	0.001578947	1940.49	0.76
218872_at	tescalcin	TESC	1.30E-05	0.001578947	1942.22	0.93
219630_at	PDZK1 interacting protein 1	PDZK1IP1	1.30E-05	0.001578947	1947.57	0.90
208710_s_at	adaptor-related protein complex 3, delta 1 subunit	AP3D1	1.52E-05	0.00175	1963.75	0.83
206371_at	folate receptor 3 (gamma)	FOLR3	1.52E-05	0.00175	1972.85	0.83
205041_s_at	orosomucoid 1	ORM1	1.52E-05	0.00175	1975.45	0.91
212816_s_at	cystathionine-beta-synthase	CBS	1.52E-05	0.00175	1975.67	0.81
205557_at	bactericidal/permeability-increasing protein	BPI	1.52E-05	0.00175	1975.74	0.92
208451_s_at	complement component 4B (Childo blood group)	C4B	1.67E-05	0.00184	1988.91	0.82

203559_s_at	amiloride binding protein 1 (amine oxidase (copper-containing))	ABP1	1.74E-05	0.001904762	1993.15	0.88
215839_at	leukocyte immunoglobulin-like receptor, subfamily A (with TM domain), member 5	LILRA5	1.74E-05	0.001904762	1993.92	0.75
203319_s_at	zinc finger protein 148	ZNF148	1.81E-05	0.001953125	2004.08	0.75
209960_at	hepatocyte growth factor (hepapoietin A; scatter factor)	HGF	1.81E-05	0.001953125	2006.82	0.79
210517_s_at	A kinase (PRKA) anchor protein 12	AKAP12	1.81E-05	0.001953125	2006.85	0.94
206025_s_at	tumor necrosis factor, alpha-induced protein 6	TNFAIP6	1.81E-05	0.001953125	2012.03	0.80
215242_at	phosphatidylinositol glycan anchor biosynthesis, class C	PIGC	1.81E-05	0.001953125	2012.08	1.04
203231_s_at	ataxin 1	ATXN1	1.88E-05	0.001954887	2015.78	0.84
201005_at	CD9 molecule	CD9	1.96E-05	0.002014926	2016.96	0.92
214036_at	not yet annotated	NA	2.03E-05	0.002074074	2021.69	0.77
204794_at	dual specificity phosphatase 2	DUSP2	2.61E-05	0.002647059	2034.99	0.80
214215_s_at	La ribonucleoprotein domain family, member 5	LARP5	2.90E-05	0.002919708	2042.10	0.76
214453_s_at	interferon-induced protein 44	IFI44	2.90E-05	0.002919708	2044.43	0.85
201333_s_at	Rho guanine nucleotide exchange factor (GEF) 12	ARHGEF12	2.90E-05	0.002919708	2045.46	0.85
206757_at	phosphodiesterase 5A, cGMP-specific	PDE5A	3.12E-05	0.003071429	2048.71	0.72
202509_s_at	tumor necrosis factor, alpha-induced protein 2	TNFAIP2	3.26E-05	0.003191489	2053.88	0.86
215990_s_at	B-cell CLL/lymphoma 6	BCL6	3.26E-05	0.003191489	2054.12	0.77
203936_s_at	matrix metallopeptidase 9 (gelatinase B, 92kDa gelatinase, 92kDa type IV collagenase)	MMP9	3.33E-05	0.003216783	2056.74	0.80
206857_s_at	FK506 binding protein 1B, 12.6 kDa	FKBP1B	3.40E-05	0.003263889	2060.67	0.89
210797_s_at	2'-5'-oligoadenylate synthetase-like	OASL	3.48E-05	0.003310345	2063.03	0.76
206785_s_at	killer cell lectin-like receptor subfamily C, member 2	KLRC2	3.62E-05	0.003424658	2072.89	0.82
219519_s_at	sialic acid binding Ig-like lectin 1, sialoadhesin	SIGLEC1	3.69E-05	0.003469388	2074.52	0.82
204533_at	chemokine (C-X-C motif) ligand 10	CXCL10	3.84E-05	0.003581081	2075.99	0.92
201392_s_at	insulin-like growth factor 2 receptor	IGF2R	4.06E-05	0.003758389	2078.86	0.78
203504_s_at	ATP-binding cassette, sub-family A (ABC1), member 1	ABCA1	4.13E-05	0.0038	2079.69	0.81
215684_s_at	activating signal cointegrator 1 complex subunit 2	ASCC2	4.56E-05	0.004172185	2087.54	0.86
221268_s_at	sphingosine-1-phosphate phosphatase 1	SGPP1	4.78E-05	0.004342105	2093.53	1.10
203961_at	nebulette	NEBL	5.00E-05	0.004509804	2102.49	1.17
220404_at	G protein-coupled receptor 97	GPR97	5.14E-05	0.00461039	2106.53	0.78
206877_at	MAX dimerization protein 1	MXD1	5.22E-05	0.004645161	2106.92	0.79
203942_s_at	MAP/microtubule affinity-regulating kinase 2	MARK2	5.29E-05	0.004679487	2110.39	0.82

Down-regulated genes

**Affymetrix probe ID**	**Gene name**	**Gene symbol**	**p-Values**	**q-Values**	**RP-Values**	**Fold Change**

203290_at	major histocompatibility complex, class II, DQ alpha 1	HLA-DQA1	0	0	192.80	4.96
214218_s_at	X (inactive)-specific transcript (non-protein coding)	XIST	0	0	367.79	2.43
221728_x_at	X (inactive)-specific transcript (non-protein coding)	XIST	0	0	413.06	2.35
217671_at	not yet annotated	NA	0	0	655.43	1.77
221491_x_at	major histocompatibility complex, class II, DR beta 3	HLA-DRB3	0	0	710.39	1.54
209841_s_at	leucine rich repeat neuronal 3	LRRN3	0	0	710.44	1.71
220281_at	solute carrier family 12 (sodium/potassium/chloride transporters), member 1	SLC12A1	0	0	844.85	1.29
210432_s_at	sodium channel, voltage-gated, type III, alpha subunit	SCN3A	0	0	891.23	1.54
209728_at	major histocompatibility complex, class II, DR beta 4	HLA-DRB4	0	0	933.19	1.12
213825_at	oligodendrocyte lineage transcription factor 2	OLIG2	0	0	950.93	1.48
219179_at	dapper, antagonist of beta-catenin, homolog 1 (Xenopus laevis)	DACT1	0	0	955.19	1.44
204141_at	tubulin, beta 2A	TUBB2A	0	0	970.29	1.18
205493_s_at	dihydropyrimidinase-like 4	DPYSL4	0	0	974.26	1.56
205826_at	myomesin (M-protein) 2, 165kDa	MYOM2	0	0	1003.57	1.25
215666_at	major histocompatibility complex, class II, DR beta 4	HLA-DRB4	0	0	1035.27	1.12
215374_at	poly(A) polymerase alpha	PAPOLA	0	0	1052.13	1.50
207328_at	arachidonate 15-lipoxygenase	ALOX15	0	0	1056.14	1.32
208253_at	sialic acid binding Ig-like lectin 8	SIGLEC8	0	0	1084.14	1.39
207674_at	Fc fragment of IgA, receptor for	FCAR	0	0	1107.21	1.11
215652_at	succinate dehydrogenase complex, subunit D, integral membrane protein	SDHD	0	0	1125.90	1.71
219532_at	elongation of very long chain fatty acids (FEN1/Elo2, SUR4/Elo3, yeast)-like 4	ELOVL4	0	0	1128.12	1.48
215083_at	not yet annotated	NA	0	0	1132.27	1.50
209840_s_at	leucine rich repeat neuronal 3	LRRN3	0	0	1141.26	1.42
213382_at	macrophage stimulating, pseudogene 9	MSTP9	0	0	1163.18	1.33
220421_at	butyrophilin-like 8	BTNL8	0	0	1166.15	1.33
209686_at	S100 calcium binding protein B	S100B	0	0	1167.24	1.10
221205_at	not yet annotated	NA	0	0	1201.03	1.44
222285_at	immunoglobulin heavy constant gamma 1 (G1m marker)	IGHG1	0	0	1213.23	1.52
206632_s_at	apolipoprotein B mRNA editing enzyme, catalytic polypeptide-like 3B	APOBEC3B	0	0	1215.96	1.30
220532_s_at	transmembrane protein 176B	TMEM176B	0	0	1222.04	0.98
203961_at	nebulette	NEBL	0	0	1247.28	1.17
218975_at	collagen, type V, alpha 3	COL5A3	0	0	1270.21	1.18
221268_s_at	sphingosine-1-phosphate phosphatase 1	SGPP1	0	0	1277.24	1.10

39318_at	T-cell leukemia/lymphoma 1A	TCL1A	0	0	1288.39	1.47
217295_at	mucin 8	MUC8	0	0	1291.13	1.21
219301_s_at	contactin associated protein-like 2	CNTNAP2	0	0	1293.74	1.39
209995_s_at	T-cell leukemia/lymphoma 1A	TCL1A	0	0	1306.93	1.47
213674_x_at	immunoglobulin heavy constant gamma 1 (G1m marker)	IGHG1	0	0	1334.25	1.46
52255_s_at	collagen, type V, alpha 3	COL5A3	0	0	1336.42	1.19
214537_at	histone cluster 1, H1d	HIST1H1D	0	0	1341.42	1.28
209583_s_at	CD200 molecule	CD200	0	0	1348.33	1.36
212558_at	sprouty homolog 1, antagonist of FGF signaling (Drosophila)	SPRY1	0	0	1348.85	1.36
205594_at	zinc finger protein 652	ZNF652	0	0	1349.81	1.49
206700_s_at	jumonji, AT rich interactive domain 1D	JARID1D	0	0	1350.60	0.56
219671_at	hippocalcin like 4	HPCAL4	0	0	1372.25	1.50
206641_at	tumor necrosis factor receptor superfamily, member 17	TNFRSF17	0	0	1405.75	1.23
206492_at	fragile histidine triad gene	FHIT	0	0	1416.26	1.44
209706_at	NK3 homeobox 1	NKX3-1	0	0	1430.14	1.03
207509_s_at	leukocyte-associated immunoglobulin-like receptor 2	LAIR2	0	0	1430.73	1.33
203911_at	RAP1 GTPase activating protein	RAP1GAP	0	0	1432.33	1.27
209371_s_at	SH3-domain binding protein 2	SH3BP2	0	0	1442.99	1.17
212768_s_at	olfactomedin 4	OLFM4	0	0	1471.99	1.04
203962_s_at	nebulette	NEBL	0	0	1481.45	1.20
218793_s_at	sex comb on midleg-like 1 (Drosophila)	SCML1	0	0	1481.71	1.25
207289_at	matrix metallopeptidase 25	MMP25	0	0	1490.41	1.34
205917_at	zinc finger protein 264	ZNF264	0	0	1504.92	1.37
219300_s_at	contactin associated protein-like 2	CNTNAP2	0	0	1506.67	1.29
215543_s_at	like-glycosyltransferase	LARGE	0	0	1514.37	1.42
215621_s_at	immunoglobulin heavy constant gamma 1 (G1m marker)	IGHG1	0	0	1523.92	1.37
221771_s_at	M-phase phosphoprotein 8	MPHOSPH8	0	0	1526.13	1.34
210121_at	UDP-Gal:betaGlcNAc beta 1,3-galactosyltransferase, polypeptide 2	B3GALT2	0	0	1527.71	1.38
206851_at	ribonuclease, RNase A family, 3 (eosinophil cationic protein)	RNASE3	0	0	1548.77	1.19
218345_at	transmembrane protein 176A	TMEM176A	0	0	1567.01	0.88
214945_at	family with sequence similarity 153, member B	FAM153B	0	0	1568.07	1.34
214407_x_at	glycophorin B (MNS blood group)	GYPB	1.45E-06	3.03E-04	1577.94	1.29
215242_at	phosphatidylinositol glycan anchor biosynthesis, class C	PIGC	2.17E-06	4.29E-04	1582.29	1.04
205000_at	DEAD (Asp-Glu-Ala-Asp) box polypeptide 3, Y-linked	DDX3Y	2.17E-06	4.29E-04	1583.06	0.56
214180_at	mannosidase, alpha, class 1C, member 1	MAN1C1	2.17E-06	4.29E-04	1589.41	1.48

205238_at	chromosome X open reading frame 34	CXorf34	2.17E-06	4.29E-04	1591.42	1.32
215761_at	Dmx-like 2	DMXL2	2.90E-06	5.13E-04	1600.27	1.35
215892_at	zinc finger protein 440	ZNF440	2.90E-06	5.13E-04	1601.37	1.38
220068_at	pre-B lymphocyte gene 3	VPREB3	2.90E-06	5.13E-04	1602.28	1.42
213268_at	calmodulin binding transcription activator 1	CAMTA1	2.90E-06	5.13E-04	1609.06	1.26
204409_s_at	eukaryotic translation initiation factor 1A, Y-linked	EIF1AY	2.90E-06	5.13E-04	1639.00	0.55
210548_at	chemokine (C-C motif) ligand 23	CCL23	2.90E-06	5.13E-04	1642.99	1.09
206873_at	carbonic anhydrase VI	CA6	2.90E-06	5.13E-04	1645.34	1.37
219389_at	sushi domain containing 4	SUSD4	2.90E-06	5.13E-04	1651.28	1.37
205389_s_at	ankyrin 1, erythrocytic	ANK1	3.62E-06	6.25E-04	1662.21	1.24
216002_at	not yet annotated	NA	3.62E-06	6.25E-04	1662.80	1.26
206310_at	serine peptidase inhibitor, Kazal type 2 (acrosin-trypsin inhibitor)	SPINK2	4.35E-06	6.59E-04	1669.91	1.35
208450_at	lectin, galactoside-binding, soluble, 2	LGALS2	4.35E-06	6.59E-04	1671.14	1.16
203038_at	protein tyrosine phosphatase, receptor type, K	PTPRK	4.35E-06	6.59E-04	1671.72	1.33
201406_at	ribosomal protein L36a	RPL36A	4.35E-06	6.59E-04	1672.52	1.18
210718_s_at	ADP-ribosylation factor-like 17 pseudogene 1	ARL17P1	4.35E-06	6.59E-04	1673.08	1.20
215786_at	not yet annotated	NA	4.35E-06	6.59E-04	1673.85	1.41
216247_at	ribosomal protein S20	RPS20	4.35E-06	6.59E-04	1675.94	1.31
201295_s_at	WD repeat and SOCS box-containing 1	WSB1	4.35E-06	6.59E-04	1681.11	1.17
210549_s_at	chemokine (C-C motif) ligand 23	CCL23	4.35E-06	6.59E-04	1688.98	1.11
201909_at	ribosomal protein S4, Y-linked 1	RPS4Y1	4.35E-06	6.59E-04	1690.66	0.48
202904_s_at	LSM5 homolog, U6 small nuclear RNA associated (S. cerevisiae)	LSM5	4.35E-06	6.59E-04	1697.36	1.34
220059_at	signal transducing adaptor family member 1	STAP1	5.80E-06	8.08E-04	1721.83	1.34
205110_s_at	fibroblast growth factor 13	FGF13	5.80E-06	8.08E-04	1722.94	1.00
210701_at	craniofacial development protein 1	CFDP1	5.80E-06	8.08E-04	1724.30	1.40
207063_at	chromosome Y open reading frame 14	CYorf14	5.80E-06	8.08E-04	1735.98	0.96
220085_at	helicase, lymphoid-specific	HELLS	5.80E-06	8.08E-04	1737.70	1.40
221170_at	histamine receptor H4	HRH4	5.80E-06	8.08E-04	1750.48	1.23
215133_s_at	family with sequence similarity 153, member B	FAM153B	5.80E-06	8.08E-04	1751.77	1.35
219672_at	erythroid associated factor	ERAF	5.80E-06	8.08E-04	1752.58	1.15
222375_at	not yet annotated	NA	6.52E-06	8.65E-04	1763.25	1.27
219815_at	galactose-3-O-sulfotransferase 4	GAL3ST4	6.52E-06	8.65E-04	1765.45	1.40
203088_at	fibulin 5	FBLN5	6.52E-06	8.65E-04	1766.94	1.28
216133_at	T cell receptor alpha locus	TRA@	6.52E-06	8.65E-04	1769.49	1.38
216022_at	not yet annotated	NA	6.52E-06	8.65E-04	1769.99	1.33
217422_s_at	CD22 molecule	CD22	7.24E-06	9.52E-04	1783.28	1.38

38521_at	CD22 molecule	CD22	7.97E-06	0.001009174	1799.78	1.37
213831_at	major histocompatibility complex, class II, DQ alpha 1	HLA-DQA1	7.97E-06	0.001009174	1800.71	0.37
207434_s_at	FXYD domain containing ion transport regulator 2	FXYD2	7.97E-06	0.001009174	1805.06	1.32
37462_i_at	splicing factor 3a, subunit 2, 66kDa	SF3A2	7.97E-06	0.001009174	1811.72	1.24
214012_at	endoplasmic reticulum aminopeptidase 1	ERAP1	8.69E-06	0.001081081	1812.83	1.12
215768_at	not yet annotated	NA	8.69E-06	0.001081081	1815.89	1.19
209602_s_at	GATA binding protein 3	GATA3	1.09E-05	0.001339286	1833.19	1.32
220729_at	not yet annotated	NA	1.16E-05	0.001415929	1836.59	1.14
204581_at	CD22 molecule	CD22	1.30E-05	0.001565217	1845.65	1.37
207979_s_at	CD8b molecule	CD8B	1.30E-05	0.001565217	1871.50	1.30
213994_s_at	spondin 1, extracellular matrix protein	SPON1	1.45E-05	0.001724138	1876.53	1.34
205624_at	carboxypeptidase A3 (mast cell)	CPA3	1.52E-05	0.001735537	1884.04	0.90
216763_at	KN motif and ankyrin repeat domains 1	KANK1	1.52E-05	0.001735537	1885.04	1.24
219215_s_at	solute carrier family 39 (zinc transporter), member 4	SLC39A4	1.52E-05	0.001735537	1888.51	1.34
202286_s_at	tumor-associated calcium signal transducer 2	TACSTD2	1.52E-05	0.001735537	1888.54	0.94
205372_at	pleiomorphic adenoma gene 1	PLAG1	1.52E-05	0.001735537	1890.74	1.25
206606_at	lipase, hepatic	LIPC	1.59E-05	0.00176	1893.08	1.28
216833_x_at	glycophorin B (MNS blood group)	GYPB	1.59E-05	0.00176	1895.37	1.25
215956_at	not yet annotated	NA	1.59E-05	0.00176	1901.20	1.28
205674_x_at	FXYD domain containing ion transport regulator 2	FXYD2	1.59E-05	0.00176	1904.65	1.35
211149_at	ubiquitously transcribed tetratricopeptide repeat gene, Y-linked	UTY	1.67E-05	0.001825397	1906.79	0.61
221747_at	tensin 1	TNS1	1.88E-05	0.002015504	1920.41	1.12
210854_x_at	solute carrier family 6 (neurotransmitter transporter, creatine), member 8	SLC6A8	1.88E-05	0.002015504	1921.63	1.06
214997_at	golgi autoantigen, golgin subfamily a, 1	GOLGA1	1.88E-05	0.002015504	1921.84	1.39
213843_x_at	solute carrier family 6 (neurotransmitter transporter, creatine), member 8	SLC6A8	1.96E-05	0.002061069	1929.31	1.07
214731_at	CTTNBP2 N-terminal like	CTTNBP2NL	1.96E-05	0.002061069	1933.62	1.32
205900_at	keratin 1	KRT1	2.10E-05	0.002101449	1936.58	0.94
219846_at	gon-4-like (C. elegans)	GON4L	2.10E-05	0.002101449	1937.43	1.35
220784_s_at	urotensin 2	UTS2	2.10E-05	0.002101449	1943.80	1.29
219308_s_at	adenylate kinase 5	AK5	2.10E-05	0.002101449	1945.58	1.34
214877_at	CDK5 regulatory subunit associated protein 1-like 1	CDKAL1	2.10E-05	0.002101449	1951.73	1.14
215555_at	not yet annotated	NA	2.10E-05	0.002101449	1953.09	1.31
209066_x_at	ubiquinol-cytochrome c reductase binding protein	UQCRB	2.10E-05	0.002101449	1958.85	1.09
205802_at	transient receptor potential cation channel, subfamily C, member 1	TRPC1	2.17E-05	0.002112676	1961.49	1.13

207815_at	platelet factor 4 variant 1	PF4V1	2.17E-05	0.002112676	1962.95	1.09
206478_at	KIAA0125	KIAA0125	2.17E-05	0.002112676	1967.15	1.28
222101_s_at	dachsous 1 (Drosophila)	DCHS1	2.17E-05	0.002112676	1968.11	1.28
213846_at	cytochrome c oxidase subunit VIIc	COX7C	2.32E-05	0.002237762	1977.92	1.17
219629_at	family with sequence similarity 118, member A	FAM118A	2.39E-05	0.002275862	1982.50	1.04
205803_s_at	transient receptor potential cation channel, subfamily C, member 1	TRPC1	2.39E-05	0.002275862	1983.15	1.05
216067_at	not yet annotated	NA	2.61E-05	0.002465753	1987.71	1.27
211310_at	enhancer of zeste homolog 1 (Drosophila)	EZH1	2.75E-05	0.002585034	1993.58	1.36
204410_at	eukaryotic translation initiation factor 1A, Y-linked	EIF1AY	3.12E-05	0.002905405	2004.20	0.59
211645_x_at	not yet annotated	NA	3.19E-05	0.00295302	2014.22	1.21
210756_s_at	Notch homolog 2 (Drosophila)	NOTCH2	3.26E-05	0.002980132	2024.31	1.28
211643_x_at	immunoglobulin kappa constant	IGKC	3.26E-05	0.002980132	2027.25	1.25
220820_at	not yet annotated	NA	3.33E-05	0.003006536	2029.46	1.30
221428_s_at	transducin (beta)-like 1 X-linked receptor 1	TBL1XR1	3.33E-05	0.003006536	2030.01	0.88
206398_s_at	CD19 molecule	CD19	3.40E-05	0.003051948	2034.81	1.30
205544_s_at	complement component (3d/Epstein Barr virus) receptor 2	CR2	3.48E-05	0.003076923	2036.66	1.29
207681_at	chemokine (C-X-C motif) receptor 3	CXCR3	3.48E-05	0.003076923	2036.94	1.30
209883_at	glycosyltransferase 25 domain containing 2	GLT25D2	3.55E-05	0.003121019	2042.43	1.20
213172_at	tetratricopeptide repeat domain 9	TTC9	3.62E-05	0.003164557	2044.84	1.33
212805_at	similar to KIAA0367	LOC100129762	3.84E-05	0.0033125	2050.93	1.07
212190_at	serpin peptidase inhibitor, clade E (nexin, plasminogen activator inhibitor type 1), member 2	SERPINE2	3.84E-05	0.0033125	2055.43	1.27
208650_s_at	CD24 molecule	CD24	3.98E-05	0.003416149	2057.55	1.24
213922_at	tau tubulin kinase 2	TTBK2	4.20E-05	0.003515152	2060.91	1.36
220637_at	family with sequence similarity 124B	FAM124B	4.20E-05	0.003515152	2062.36	1.24
220494_s_at	not yet annotated	NA	4.20E-05	0.003515152	2063.69	1.09
209807_s_at	nuclear factor I/X (CCAAT-binding transcription factor)	NFIX	4.20E-05	0.003515152	2065.51	1.09
219315_s_at	transmembrane protein 204	TMEM204	4.27E-05	0.003532934	2066.26	1.33
74694_s_at	rabaptin, RAB GTPase binding effector protein 2	RABEP2	4.27E-05	0.003532934	2066.42	1.30
217647_at	not yet annotated	NA	4.42E-05	0.003609468	2069.88	1.39
221477_s_at	superoxide dismutase 2, mitochondrial	SOD2	4.42E-05	0.003609468	2073.28	1.17
217397_at	T cell receptor alpha locus	TRA@	4.64E-05	0.003657143	2088.15	1.29
201151_s_at	muscleblind-like (Drosophila)	MBNL1	4.64E-05	0.003657143	2088.44	0.98
203476_at	trophoblast glycoprotein	TPBG	4.64E-05	0.003657143	2089.94	1.20
222253_s_at	POM121-like protein	DKFZP434P211	4.64E-05	0.003657143	2090.52	1.15
207459_x_at	glycophorin B (MNS blood group)	GYPB	4.64E-05	0.003657143	2091.80	1.18
209381_x_at	splicing factor 3a, subunit 2, 66kDa	SF3A2	4.64E-05	0.003657143	2094.31	1.20

206088_at	leucine rich repeat containing 37A	LRRC37A	4.85E-05	0.003806818	2097.24	1.18
206624_at	ubiquitin specific peptidase 9, Y-linked (fat facets-like, Drosophila)	USP9Y	4.93E-05	0.003841808	2100.00	0.69
78383_at	similar to hCG1811779	LOC100129250	5.14E-05	0.003988764	2103.08	1.33
214666_x_at	iron-responsive element binding protein 2	IREB2	5.22E-05	0.003956044	2108.86	1.15
210744_s_at	interleukin 5 receptor, alpha	IL5RA	5.22E-05	0.003956044	2109.45	1.14
213804_at	inositol polyphosphate-5-phosphatase, 75kDa	INPP5B	5.22E-05	0.003956044	2113.64	1.35
217539_at	chromosome 18 open reading frame 25	C18orf25	5.22E-05	0.003956044	2114.26	1.32
216401_x_at	similar to Ig kappa chain V-I region HK102 precursor	LOC652493	5.29E-05	0.003924731	2116.63	1.26
206171_at	adenosine A3 receptor	ADORA3	5.29E-05	0.003924731	2116.97	1.12
209816_at	patched homolog 1 (Drosophila)	PTCH1	5.29E-05	0.003924731	2120.32	1.18
211343_s_at	collagen, type XIII, alpha 1	COL13A1	5.29E-05	0.003924731	2121.77	1.27
209662_at	centrin, EF-hand protein, 3 (CDC31 homolog, yeast)	CETN3	5.36E-05	0.00393617	2125.17	1.09
215812_s_at	solute carrier family 6 (neurotransmitter transporter, creatine), member 10 (pseudogene)	SLC6A10P	5.36E-05	0.00393617	2125.32	1.04
207520_at	TROVE domain family, member 2	TROVE2	5.72E-05	0.004179894	2132.17	1.20
208428_at	transporter 2, ATP-binding cassette, sub-family B (MDR/TAP)	TAP2	5.94E-05	0.004315789	2135.72	1.14
215648_at	not yet annotated	NA	6.01E-05	0.004322917	2138.06	1.31
205049_s_at	CD79a molecule, immunoglobulin-associated alpha	CD79A	6.01E-05	0.004322917	2138.08	1.28
201754_at	cytochrome c oxidase subunit VIc	COX6C	6.23E-05	0.004410257	2142.04	1.11
209480_at	major histocompatibility complex, class II, DQ beta 1	HLA-DQB1	6.23E-05	0.004410257	2142.25	0.42
211106_at	not yet annotated	NA	6.23E-05	0.004410257	2142.87	1.28
209871_s_at	amyloid beta (A4) precursor protein-binding, family A, member 2	APBA2	6.37E-05	0.004489796	2146.96	1.37
213534_s_at	PAS domain containing serine/threonine kinase	PASK	6.66E-05	0.004646465	2149.68	1.27
34478_at	RAB11B, member RAS oncogene family	RAB11B	6.66E-05	0.004646465	2149.81	1.25
205732_s_at	nuclear receptor coactivator 2	NCOA2	6.81E-05	0.0047	2151.61	1.07
204612_at	protein kinase (cAMP-dependent, catalytic) inhibitor alpha	PKIA	6.81E-05	0.0047	2153.24	1.29
214973_x_at	immunoglobulin heavy constant delta	IGHD	6.95E-05	0.004752475	2153.53	1.28
205950_s_at	carbonic anhydrase I	CA1	6.95E-05	0.004752475	2154.85	1.08
203178_at	glycine amidinotransferase (L-arginine:glycine amidinotransferase)	GATM	7.10E-05	0.004803922	2156.97	1.18
220177_s_at	transmembrane protease, serine 3	TMPRSS3	7.10E-05	0.004803922	2157.01	1.29
215176_x_at	netrin 2-like (chicken)	NTN2L	7.17E-05	0.004782609	2157.90	1.23
211517_s_at	interleukin 5 receptor, alpha	IL5RA	7.17E-05	0.004782609	2159.27	1.15
216576_x_at	netrin 2-like (chicken)	NTN2L	7.17E-05	0.004782609	2160.55	1.20
206507_at	zinc finger and SCAN domain containing 12	ZSCAN12	7.46E-05	0.004951923	2164.55	1.28
