# Supplementary material for: Blood transcriptomics of drug-naïve sporadic Parkinson’s disease patients
Source: BMC Genomics. 2015 Oct 28;16:876. doi: 10.1186/s12864-015-2058-3 (PMC4625854; doi:10.1186/s12864-015-2058-3)
Supplement: Additional file 9: — List of common differentially expressed genes between PD patients and controls based on SAM and PUMA analyses and the 395 selected variables of the Ranking-PCA. Genes are ranked according to the P- values based on PUMA analysis. Order of the 395 variables, Affymetrix Probe Set IDs, gene symbols and names are indicated. Names shown in bold indicate transcripts validated by RT-qPCR (Fig. 2a). (PDF 3122 kb) [file 12864_2015_2058_MOESM9_ESM.pdf]

Additional file 9. List of common differentially expressed genes between PD patients and controls based on SAM and PUMA analyses and the 395 selected variables of the Ranking-PCA. Genes are ranked according to the P- values based on PUMA analysis. Order of the 395 variables, Affymetrix Probe Set IDs, gene symbols and names are indicated. Names shown in bold indicate transcripts validated by RT-qPCR (Figure 2a).

Affymetrix ID	Gene symbol	Gene name	P-value	PUMA: Ratio	SAM: Ratio	Order
**209715_at**	**CBX5**	**chromobox homolog 5 (HP1 alpha homolog, Drosophila)**	**5.67E-08**	**-1.38**	**-1.35**	**1**
208893_s_at	dusp6	dual specificity phosphatase 6	7.48E-05	1.37	1.37	8
**209153_s_at**	**tcf3**	**transcription factor 3 (E2A immunoglobulin enhancer binding factors E12/E47)**	**8.62E-05**	**-1.26**	**-1.33**	**7**
213006_at	cebpd	CCAAT/enhancer binding protein (C/EBP), delta	1.28E-04	1.31	1.35	81
203658_at	SLC25A20	solute carrier family 25 (carnitine/acylcarnitine translocase), member 20	1.32E-04	1.26	1.29	34
202436_s_at	CYP1B1	cytochrome P450, family 1, subfamily B, polypeptide 1	1.97E-04	1.44	1.48	19
204530_s_at	Tox	thymocyte selection-associated high mobility group box	2.42E-04	1.29	1.29	220
**215151_at**	**dock10**	**dedicator of cytokinesis 10**	**2.47E-04**	**-1.23**	**-1.26**	**128**
221592_at	TBC1D8	TBC1 domain family, member 8 (with GRAM domain)	2.48E-04	1.28	1.33	9
219025_at	CD248	CD248 molecule, endosialin	2.84E-04	-2.17	-1.30	31
**214180_at**	**MAN1C1**	**mannosidase, alpha, class 1C, member 1**	**2.95E-04**	**-1.37**	**-1.39**	**40**
213435_at	SATB2	SATB homeobox 2	3.65E-04	1.59	1.67	149
210607_at	FLT3LG	fms-related tyrosine kinase 3 ligand	3.97E-04	-1.35	-1.34	18
205594_at	ZNF652	zinc finger protein 652	4.20E-04	-1.41	-1.57	33
205530_at	Etfdh	electron-transferring-flavoprotein dehydrogenase	4.93E-04	1.19	1.22	24
201013_s_at	paics	phosphoribosylaminoimidazole carboxylase, phosphoribosylaminoimidazole succinocarboxamide synthetase	4.98E-04	-1.16	-1.21	97
210847_x_at	TNFRSF25	tumor necrosis factor receptor superfamily, member 25	5.03E-04	-1.26	-1.29	12
214739_at	LRCH3	leucine-rich repeats and calponin homology (CH) domain containing 3	6.26E-04	-1.17	-1.25	6
212996_s_at	URB1	URB1 ribosome biogenesis 1 homolog (S. cerevisiae)	6.26E-04	-1.24	-1.28	163
213666_at	Sept6	septin 6	8.53E-04	-1.21	-1.24	153
217647_at	dhodh	dihydroorotate dehydrogenase	9.45E-04	-1.36	-1.43	36
215750_at	KIAA1659	KIAA1659 protein	9.86E-04	-1.32	-1.53	2
220609_at	LOC202181	hypothetical protein LOC202181	1.11E-03	-1.19	-1.24	47
218693_at	tspan15	tetraspanin 15	1.11E-03	-1.40	-1.36	57
207339_s_at	LTB	lymphotoxin beta (TNF superfamily, member 3)	1.15E-03	-1.22	-1.26	3
219671_at	HPCAL4	hippocalcin like 4	1.22E-03	-1.37	-1.51	37
203630_s_at	COG5	component of oligomeric golgi complex 5	1.29E-03	-1.25	-1.28	114
202658_at	PEX11B	peroxisomal biogenesis factor 11 beta	1.43E-03	-1.12	-1.13	158
214661_s_at	NOP14	NOP14 nucleolar protein homolog (yeast)	1.57E-03	-1.17	-1.20	16
211310_at	EZH1	enhancer of zeste homolog 1 (Drosophila)	1.73E-03	-1.27	-1.29	346
31637_s_at	thrA	thyroid hormone receptor, alpha (erythroblastic leukemia viral (v-erb-a) oncogene homolog, avian)	1.97E-03	-1.29	-1.39	23
212831_at	MEGF9	multiple EGF-like-domains 9	2.03E-03	1.20	1.29	41
217645_at	COX16	COX16 cytochrome c oxidase assembly homolog (S. cerevisiae)	2.05E-03	-1.23	-1.28	78
203825_at	BRD3	bromodomain containing 3	2.58E-03	-1.21	-1.23	68
213963_s_at	SAP30	Sin3A-associated protein, 30kDa	2.76E-03	1.28	1.35	13
222348_at	MAST4	microtubule associated serine/threonine kinase family member 4	3.00E-03	-1.21	-1.31	22
219315_s_at	TMEM204	transmembrane protein 204	3.30E-03	-1.27	-1.36	125
213804_at	INPP5B	inositol polyphosphate-5-phosphatase, 75kDa	3.50E-03	-1.26	-1.34	58
220215_at	ZNF669	zinc finger protein 669	4.16E-03	-1.14	-1.20	61
207078_at	MED6	mediator complex subunit 6	4.40E-03	-1.20	-1.27	46
215390_at	c9orf5	chromosome 9 open reading frame 5	4.79E-03	-1.46	-1.65	121
222027_at	Nucks1	nuclear casein kinase and cyclin-dependent	4.94E-03	-1.19	-1.27	120

		kinase substrate 1				
219846_at	YY1AP1//GON4L	YY1 associated protein 1; gon-4-like (C. elegans)	5.21E-03	-1.28	-1.39	87
206413_s_at	TCL6	T-cell leukemia/lymphoma 6	5.25E-03	-1.46	-1.23	29
221101_at	C14ORF113	chromosome 14 open reading frame 113	5.76E-03	-1.55	-1.50	132
222141_at	Klhl22	kelch-like 22 (Drosophila)	5.90E-03	-1.17	-1.21	95
206761_at	Cd96	CD96 molecule	6.00E-03	-1.25	-1.27	71
220085_at	Hells	helicase, lymphoid-specific	6.10E-03	-1.27	-1.35	51
212414_s_at	Sept6	septin 6	6.60E-03	-1.14	-1.19	77
41113_at	ZNF500	zinc finger protein 500	6.78E-03	-1.15	-1.18	44
203226_s_at	Tspan31	tetraspanin 31	7.42E-03	-1.12	-1.19	151
215786_at	rsf1	remodeling and spacing factor 1	8.66E-03	-1.26	-1.39	90
217995_at	SQRDL	sulfide quinone reductase-like (yeast)	9.81E-03	1.12	1.17	85
213340_s_at	KIAA0495	KIAA0495	9.94E-03	-1.11	-1.15	109
