# Supplementary material for: Blood transcriptomics of drug-naïve sporadic Parkinson’s disease patients
Source: BMC Genomics. 2015 Oct 28;16:876. doi: 10.1186/s12864-015-2058-3 (PMC4625854; doi:10.1186/s12864-015-2058-3)
Supplement: Additional file 10: — List of common genes between A9 DA neurons of the SN in the mouse and differentially expressed transcripts in the blood of PD patients according to SAM (a) and PUMA (b). (PDF 3122 kb) [file 12864_2015_2058_MOESM10_ESM.pdf]

Additional file 10. List of common genes between A9 dopaminergic neurons of the SN in the mouse and differentially expressed transcripts in the blood of PD patients according to SAM (a) and PUMA (b).

(a)

Common A9 and SAM analysis
ACACA
APBA2
BRD3
CBX5
CFDP1
CORO2B
DUSP6
ELOVL4
ENC1
ETFDH
EZH1
GLTSCR2
GOLGA1
GRSF1
HPCAL4
ID3
INPP5B
KLC2
KLHL22
LRRN3
MAN1C1
MCCC2
MCF2L
MED6
MEGF9
NMT2
NOL14
NPAS2
NUCKS1
OSBPL9
PAICS
PAIP2B
POLE3
RFX3
RSF1
SALL2
SAP30
SEPT6
SLC11A2
SLC25A20
SQRDL
TBC1D8
THRA
TOPORS
TOX
TSC22D3

TSPAN15
TSPAN31
TTBK2

(b)

Common A9 and co-expression analysis
AASDHPPT
ABLIM1
ACACA
ACSBG1
AHCYL1
ALG8
ANKRD57
APBA2
APLP2
B3GALT2
BAALC
BDH1
BRD3
CBX5
CBY1
CCDC85B
CCDC9
CHD3
CMKLR1
CNNM4
CORO2B
CREM
CRIP2
CSNK2A1
CTSZ
DDX51
DLGAP4
DPYSL3
DTNB
DUSP1
DUSP6
ELOVL4
ENC1
ETFDH
EXT1
EZH1
FEM1B
FGFR1OP
FKBP5
GJB3
GNAO1
GOLGA1
GPR17
H2AFY
HDAC6
HERPUD1
HIP1R
HMGA1

HPCAL4
ICAM1
IL21R
ING1
INPP4A
INPP4B
INPP5B
INTS1
KCND2
KCNK1
KCNMA1
KIF1C
KLC2
KLHL22
KPNA6
LARP5
LRRN3
LUZP2
MAN1C1
MCF2L
MECP2
MED6
MEGF9
METTL1
MGAT5
MICAL3
MLLT10
MYO5A
MYO6
NBL1
NF1
NMT2
NOL9
NPM3
NR4A2
NRG1
NUAK1
NUCKS1
NUDT6
NUP50
OSBPL1A
PAICS
PCBP1
PCDH17
PCNX
PDLIM5
PDXDC1
PHIP
PLOD1
POU3F1
PPAN
PPP1R9A
PTPRR
PUS1
RAP2A

RAP2B
RBM15B
RRBP1
RUFY3
S1PR2
SAP30
SEPT6
SGTA
SIN3B
SLC22A17
SLC25A20
SMAD1
SOCS7
SON
SPON1
SPTLC2
SQRDL
ST3GAL6
TBC1D8
TCF7
THRA
TLE4
TMEM204
TNPO1
TOX
TRPC4
TSPAN15
TSPAN31
USP22
VCAN
WDSOF1
ZBTB38
