# Supplementary material for: Blood transcriptomics of drug-naïve sporadic Parkinson’s disease patients
Source: BMC Genomics. 2015 Oct 28;16:876. doi: 10.1186/s12864-015-2058-3 (PMC4625854; doi:10.1186/s12864-015-2058-3)
Supplement: Additional file 11: — Statistically significant gene sets from GSEA analyses. GSEA was run on the normalized, unfiltered microarray dataset as suggested in the tools implementation (http://www.broadinstitute.org/gsea/ version 2.06), and using the c5 - GO gene sets collection of the Molecular Signatures Database (MSigDB) (http://www.broadinstitute.org/gsea/msigdb/). The test was performed separately on each of the c5 sub-collections (biological process, molecular function and cellular component), running 1000 permutations and excluding gene sets with fewer than 5 genes or more than 150 (the latter, to retain granularity). Names (Gene Symbol) shown in bold indicate transcripts measured by RT-qPCR (Additional file 12). (PDF 3122 kb) [file 12864_2015_2058_MOESM11_ESM.pdf]

Additional file 11. Statistically significant gene sets resulting from GSEA analyses. GSEA was run on the normalized, unfiltered microarray dataset as suggested in the tools implementation (http://www.broadinstitute.org/gsea version 2.06), and using the c5 - GO gene sets collection of the Molecular Signatures Database (MSigDB) (http://www.broadinstitute.org/gsea/msigdb/). The test was performed separately on each of the c5 sub-collections (biological process, molecular function and cellular component), running 1000 permutations and excluding gene sets with fewer than 5 genes or more than 150 (the latter, to retain granularity). Name (Gene Symbol) shown in bold indicate transcripts measured by RT-qPCR (Additional file 12).

GSEA: Gene Ontology: Process (results ID 1280852587546)
CHROMATIN_REMODELING
CHROMATIN_ASSEMBLY_OR_DISASSEMBLY

GSEA: Gene Ontology: Function (results ID 1280908995046)
S_ADENOSYLMETHIONINE_DEPENDENT_METHYLTRANSFERASE_ACTIVITY
CYTOCHROME_C_OXIDASE_ACTIVITY
TRANSFERASE_ACTIVITY__TRANSFERRING_ONE_CARBON_GROUPS
PROTEIN_METHYLTRANSFERASE_ACTIVITY
METHYLTRANSFERASE_ACTIVITY

GSEA: Gene Ontology: Component (results ID 1280909452203)
INTRINSIC_TO_GOLGI_MEMBRANE

Genes that contribute to significance of gene sets above (in order of relevance):
CHROMATIN_REMODELING:
HELLS
SUV39H2
RSF1
ACTL6A
BNIP3
SIRT4
HMGA1

CHROMATIN_ASSEMBLY_OR_DISASSEMBLY:
HELLS
SUV39H2
NAP1L3
RSF1
SIRT4
HMGA1
MTA2
CHAF1A
NAP1L4
ASF1A

S_ADENOSYLMETHIONINE_DEPENDENT_METHYLTRANSFERASE_ACTIVITY:
SUV39H2

METTL1
SUV39H1
BHMT
DNMT3A
DNMT1
PEMT
DNMT3B
PRMT7

CYTOCHROME_C_OXIDASE_ACTIVITY:
CYB5A
COX11
COX7B
COX4I1
COX10
COX5B

TRANSFERASE_ACTIVITY__TRANSFERRING_ONE_CARBON_GROUPS:
SUV39H2
GATM
METTL1
SUV39H1
BHMT
DNMT3A
DNMT1
GAMT
PRMT1
PEMT
DNMT3B
PRMT7
MGMT

PROTEIN_METHYLTRANSFERASE_ACTIVITY:
SUV39H2
SUV39H1
PRMT7

METHYLTRANSFERASE_ACTIVITY:
SUV39H2
METTL1
SUV39H1
BHMT
DNMT3A
DNMT1
GAMT
PRMT1
PEMT
DNMT3B
PRMT7
MGMT

INTRINSIC_TO_GOLGI_MEMBRANE:
LARGE
MAN1C1
BNIP3
RHOT2
