# Supplementary material for: Blood transcriptomics of drug-naïve sporadic Parkinson’s disease patients
Source: BMC Genomics. 2015 Oct 28;16:876. doi: 10.1186/s12864-015-2058-3 (PMC4625854; doi:10.1186/s12864-015-2058-3)
Supplement: Additional file 12: — Genes involved in chromatin remodeling and methylation are targeted in PD. Selected samples (12 PD and 12 HC blood samples) previously processed for gene expression profiling were also tested in RT-qPCR assays for several targets (CBX5, HELLS, MECP2, ASF1A, DNMT3A and PRMT1) that were identified by data analyses. Retrotranscription was performed using 1 μg of RNA and the iSCRIPT™ cDNA Synthesis Kit (Bio-Rad) according to the manufacturer’s protocol. Real Time qPCR was executed using SYBER-Green PCR Master Mix (Applied Biosystem) and an iCycler IQ Real Time PCR System (Bio-Rad). Sequences of gene specific primers are reported in the table below. Expression of the gene of interest was normalized to β-actin. The relative expression of each sample was calculated by the formula 2 exp-ΔΔCt (User Bulletin 2 of the ABI Prism 7700 Sequence Detection System). The amplified products were separated on a 2 % agarose gel and visualized with ethidium bromide staining. (PDF 3122 kb) [file 12864_2015_2058_MOESM12_ESM.pdf]

Additional file 12. Genes involved in chromatin remodeling and methylation are targeted in PD. Selected samples (12 PD and 12 HC blood samples) previously processed for gene expression profiling were also tested in RT-qPCR assays for several targets (CBX5, HELLS, MECP2, ASF1A, DNMT3A and PRMT1) that were identified by data analyses. Retrotranscription was performed using 1µg of RNA and the iSCRIPT™ cDNA Synthesis Kit (Bio-Rad) according to the manufacturer's protocol. Real Time qPCR was executed using SYBER-Green PCR Master Mix (Applied Biosystem) and an iCycler IQ Real Time PCR System (Bio-Rad). Sequences of gene specific primers are reported in the table below. Expression of the gene of interest was normalized to *β-actin*. The relative expression of each sample was calculated by the formula 2 exp$^{-\Delta\Delta Ct}$ (User Bulletin 2 of the ABI Prism 7700 Sequence Detection System). The amplified products were separated on a 2% agarose gel and visualized with ethidium bromide staining.

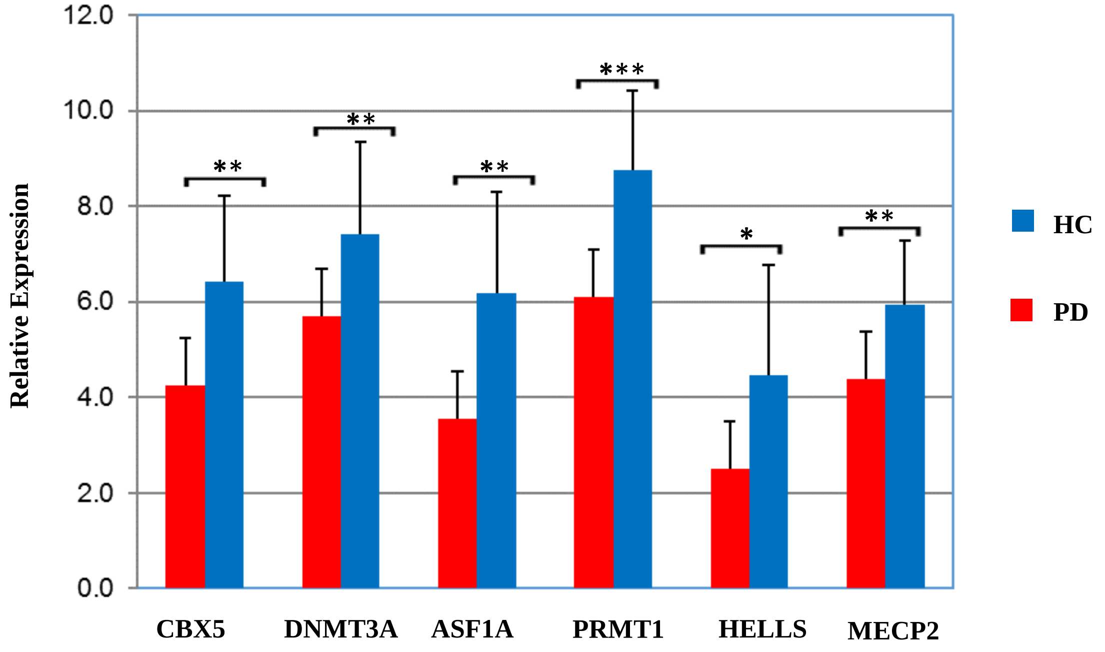

Gene symbol	Forward_Primer	Reverse_Primer
CBX5	CCAATTTCTCAAACAGTGCCGATG	GGTTCCAGTCCTCTCTCAAAGC
HELLS	GCAAGTGAAGAGAAGCCAGTTATG	CACACAGAGATTAGTAGAGGAGGAG
ASF1A	TCTCCTTTCTACAACCCGTTCC	CTTGATCGTATTCTTCACTTTCTGC
DNMT3A	GCTGCGGCGGCGAGAG	TGTAGCGGTCCACCTGAATGC
PRMT1	GCCTCCAGCCGCCTCTTG	ACCTCGTCCTTCAGCATCTCC
MECP2	CACGGAAGCTTAAGCAAAGG	CTGGAGCTTTGGGAGATTTG
