# Supplementary material for: Blood transcriptomics of drug-naïve sporadic Parkinson’s disease patients
Source: BMC Genomics. 2015 Oct 28;16:876. doi: 10.1186/s12864-015-2058-3 (PMC4625854; doi:10.1186/s12864-015-2058-3)
Supplement: Additional file 13: — Selection of reliable reference genes for peripheral blood gene expression analyses. TaqMan® array human endogenous control cards (Applied Biosystems, Foster City, CA, USA) are 384-well microfluidic cards containing 16 human TaqMan Gene Expression Assays. They were used to evaluate the endogenous controls specific for peripheral blood that exhibit minimal differential expression. Peripheral blood samples from 8 PD and 8 HC gender- and age-matched subjects were processed following the manufacturer’s instructions (Applied Biosystems, Foster City, CA, USA). The expression stability was determined and compared by two commonly used algorithms (geNorm and NormFinder). By comparing the output of these two methods and by accepting gene expression levels of qPCR at Ct values ≤ 29, we obtained a list of the most stable reference genes in human peripheral blood. See the following Figures reporting output files of the analyses. As the best reference genes, PGK1, UBC and GAPDH were selected according to the following practical considerations: PGK1 is the best reference gene according to geNorm and NormFinder analyses; UBC presents similar stability strength to PGK1 and a different threshold Ct value; GAPDH is one of the most widely used reference genes in peripheral blood expression studies. The gene expression analyses of the first experimental data sets (data not shown) reveled that GAPDH had a higher variability compared to PGK1 and UBC; therefore, we decided to evaluate the relative gene expression by normalizing the data to the geometric mean of PGK1 and UBC. (PDF 3122 kb) [file 12864_2015_2058_MOESM13_ESM.pdf]

Additional file 13. Selection of reliable reference genes for peripheral blood gene expression analyses. TaqMan® array human endogenous control cards (Applied Biosystems, Foster City, CA, USA), which are 384-well microfluidic cards containing 16 human TaqMan Gene Expression Assays. They were used to evaluate the endogenous controls specific for peripheral blood that exhibit minimal differential expression. Peripheral blood samples from 8 PD and 8 HC gender- and age-matched subjects were processed following the manufacturer's instructions (Applied Biosystems, Foster City, CA, USA). The expression stability was determined and compared by two commonly used algorithms (geNorm and NormFinder). By comparing the output of these two methods and by accepting gene expression levels of qPCR at Ct values≤ 29, we obtained a list of the most stable reference genes in human peripheral blood. See the following Figures reporting output files of the analyses. As the best reference genes, PGK1, UBC and GAPDH were selected according to the following practical considerations: PGK1 is the best reference gene according to geNorm and NormFinder analyses; UBC presents similar stability strength to PGK1 and a different threshold Ct value; GAPDH is one of the most widely used reference genes in peripheral blood expression studies. The gene expression analyses of the first experimental data sets (data not shown) reveled that GAPDH had a higher variability compared to PGK1 and UBC; therefore, we decided to evaluate the relative gene expression by normalizing the data to the geometric mean of PGK1 and UBC.

NormFinder Output

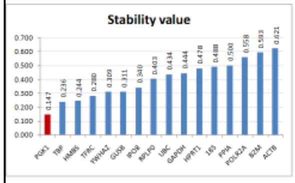

**Best gene**	PGK1
Stability value	0.147
**Best combination of two genes**	PGK1 and HMBS
Stability value for best combination of two genes	0.128

**Intragroup variation**		
Group identifier	1	0
PGK1	0.022	0.005
TBP	0.081	0.029
**HMBS**	0.150	0.124
HPRT1	0.158	0.164
B2M	0.158	0.195
18S	0.160	0.266
UBC	0.268	0.304
RPLP0	0.279	0.319
GUSB	0.333	0.394
IPO8	0.382	0.568
POLR2A	0.406	0.681
YWHAZ	0.434	0.691
GAPDH	0.459	0.799
PPIA	0.464	0.917
TFRC	0.647	1.193
ACTB	0.851	1.457

**Intergroup variation**		
Group identifier	1	0
B2M	-0.480	0.480
HPRT1	-0.308	0.308
PPIA	-0.280	0.280
UBC	-0.257	0.257
RPLP0	-0.137	0.137
**PGK1**	-0.084	0.084
TBP	-0.062	0.062
GUSB	-0.027	0.027
YWHAZ	0.010	-0.010
TFRC	0.038	-0.038
**HMBS**	0.054	-0.054
IPO8	0.101	-0.101
GAPDH	0.222	-0.222
18S	0.338	-0.338
POLR2A	0.347	-0.347
ACTB	0.524	-0.524

geNorm Output

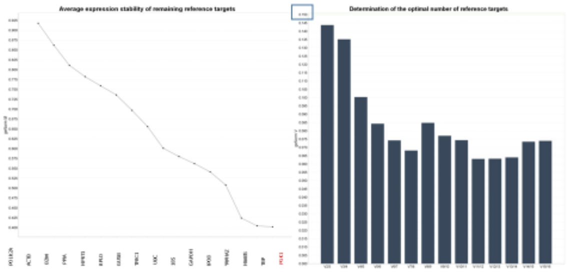

The optimal normalization factor can be calculated as the geometric mean of reference targets **TBP** and PGK1.

Reference Genes: Mean CT values

Gene ID	Average Ct
**18S**	13.85
**B2M**	20.55
**RPLPO**	24.54
**PPIA**	25.15
**UBC**	25.69
**PGK1**	26.03
**ACTB**	26.12
**GAPDH**	27.07
**YWHAZ**	29.63
**HPRT1**	29.67
**GUSB**	29.7
**TFRC**	30.23
**IPO8**	30.25
**POLR2A**	30.64
**TBP**	31.56
**HMBS**	32.02
