# Supplementary material for: Blood transcriptomics of drug-naïve sporadic Parkinson’s disease patients
Source: BMC Genomics. 2015 Oct 28;16:876. doi: 10.1186/s12864-015-2058-3 (PMC4625854; doi:10.1186/s12864-015-2058-3)
Supplement: Additional file 14: — List of similar- and anti-similar-to-PD compounds from drug network analysis. Community identifiers, drugs and community enrichment p-values resulting from the drug network analysis of genome-wide ranked lists of genes sorted according to their differential expression in PD. Drugs are sorted according to their similarity to PD (a) and according to their “anti-similarity” (b) as explained in the main manuscript. (PDF 3121 kb) [file 12864_2015_2058_MOESM14_ESM.pdf]

Additional file 14. List of similar- and anti-similar-to-PD compounds from drug network analysis. Community identifiers, drugs and community enrichment P-values resulting from the drug network analysis of genome-wide ranked lists of genes sorted according to their differential expression in PD. Drugs are sorted according to their similarity to PD (**a**) and according to their "anti-similarity" (**b**) as explained in the main manuscript.

(a)

Community	Drug	Enrichment P-value
63	helveticoside	8.99E-01
100	metixene	1.21E+01
100	chlorprothixene	1.03E+00
63	lanatoside_C	4.09E-02
100	alexidine	1.85E-01
63	proscillaridin	9.34E-04
100	levomepromazine	3.45E-02
63	digoxin	2.00E-05
62	niclosamide	2.02E+01
28	geldanamycin	4.03E+00
106	zardaverine	7.85E+00
100	methylbenzethonium_chloride	3.56E-02
63	ouabain	1.94E-06
100	perphenazine	6.98E-03
102	terguride	1.81E+01
104	etacrynic_acid	2.03E+01
63	lycorine	8.54E-08
100	trifluoperazine	3.44E-03
100	piperacetazine	4.32E-04
100	cytochalasin_B	5.02E-05
62	felodipine	8.72E+00
100	chlorcyclizine	9.51E-06
34	terfenadine	2.94E+01
62	0179445-0000	1.70E+00
81	serotonin	9.85E+00
104	15-delta_prostaglandin_J2	4.60E+00
63	digoxigenin	2.31E-08
16	pergolide	3.14E+01
100	loperamide	1.95E-05
60	clomipramine	3.50E+01
100	calmidazolium	3.83E-06
15	Gly-His-Lys	1.01E+01
40	mometasone	6.37E+01
13	clonidine	5.19E+01
40	puromycin	2.57E+01
40	betazole	7.32E+00
64	pentetrazol	1.43E+01
100	desipramine	5.53E-06
100	0297417-0002B	7.57E-07
69	fenbendazole	2.86E+01
89	sanguinarine	7.01E+01
7	diazoxide	6.00E+01
60	1,5-isoquinolinediol	1.12E+01
34	nordihydroguaiaretic_acid	1.29E+01

40	loxapine	3.13E+00
62	gossypol	2.00E+00
32	cefalotin	8.18E+01
102	altretamine	1.22E+01
38	glycopyrronium_bromide	2.19E+01
22	phenelzine	4.01E+01
63	strophanthidin	4.91E-08
104	securinine	2.90E+00
100	protriptyline	8.37E-06
100	chlorpromazine	1.36E-06
89	N-acetyl-L-leucine	4.36E+01
104	withaferin_A	5.05E-01
104	thiostrepton	5.82E-02
34	prenylamine	4.49E+00
102	biotin	3.43E+00
6	hydroxyzine	6.34E+01
62	clofazimine	1.15E+00
89	menadione	2.17E+01
40	disulfiram	2.50E+00
16	trimethobenzamide	1.95E+01
100	fluphenazine	3.63E-06
100	prochlorperazine	6.24E-07
69	mebendazole	9.53E+00
34	suloctidil	1.26E+00
40	celastrol	8.88E-01
40	5224221	2.00E-01
100	quinisocaine	3.06E-07
63	digitoxigenin	1.51E-08
52	carbachol	8.82E+01
100	diperodon	8.70E-08
104	parthenolide	2.32E-02
100	benzethonium_chloride	1.85E-08
40	fendiline	6.87E-02
40	1,4-chrysenequinone	1.27E-02
4	syrosingopine	7.65E+01
62	pyrvinium	8.40E-01
62	benzamil	1.83E-01
7	buspirone	5.05E+01
32	Prestwick-664	7.72E+01
32	0175029-0000	4.95E+01
69	scoulerine	2.43E+00
41	etofylline	4.91E+01
100	podophyllotoxin	4.18E-08
100	cloperastine	7.00E-09
40	thapsigargin	6.11E-03
5	azathioprine	8.79E+01
104	C-75	7.33E-03
62	pararosaniline	7.60E-02
5	isocarboxazid	5.55E+01
55	suxibuzone	3.33E+01
23	NS-398	2.79E+01
100	bepridil	6.76E-09
34	mefloquine	8.51E-01
5	N-acetyl-L-aspartic_acid	2.84E+01

89	ketoprofen	2.52E+01
14	bromopride	7.47E+01
0	metoclopramide	1.07E+02
40	prilocaine	3.47E-03
90	beta-escin	1.07E+02
100	homochlorcyclizine	6.13E-09
34	astemizole	2.07E-01
16	spironolactone	1.43E+01
100	clemastine	1.68E-09
13	atropine	6.69E+01
40	MG-132	1.07E-03
102	phenylpropanolamine	4.17E+00
67	letrozole	6.30E+01
102	flucytosine	9.11E-01
92	terconazole	5.51E+01
60	josamycin	1.92E+01
52	bephenium_hydroxynaphthoate	8.10E+01
32	Prestwick-665	4.92E+01
0	nefopam	1.06E+02
53	anisomycin	4.64E+01
62	dequalinium_chloride	9.31E-02
73	bromocriptine	6.21E+01
62	profenamine	2.05E-02
73	(-)-isoprenaline	2.16E+01
60	tolfenamic_acid	7.22E+00
40	piperlongumine	6.95E-04
73	orciprenaline	4.98E+00
100	dihydroergocristine	1.50E-08
28	alvespimycin	8.22E+00
89	terbutaline	2.30E+01
0	nicardipine	1.06E+02
106	dinoprost	2.38E+01
100	imipramine	6.30E-09
26	lisuride	5.05E+01
85	delsoline	7.05E+01
62	abamectin	9.21E-03
42	dirithromycin	8.63E+01
7	droperidol	5.25E+01
40	(+)-chelidonine	3.59E-04
63	hydroquinine	1.35E-07
62	5707885	2.23E-03
52	levamisole	6.84E+01
7	oxaprozin	2.99E+01
42	glibenclamide	5.31E+01
28	monorden	1.16E+00
52	bendroflumethiazide	4.54E+01
100	metitepine	1.46E-08
62	valinomycin	5.90E-04
42	chlorzoxazone	2.49E+01
53	propofol	1.50E+01
25	epirizole	7.52E+01
86	adipiodone	4.13E+01
0	terazosin	1.05E+02
5	ciprofloxacin	3.22E+01

73	fenoterol	1.51E+00
20	mexiletine	5.66E+01
16	diprophylline	1.18E+01
100	nortriptyline	1.75E-08
60	wortmannin	4.64E+00
101	salbutamol	5.76E+01
49	exemestane	8.45E+01
42	lobeline	1.07E+01
100	fluspirilene	7.14E-09
38	calcium_folinate	1.75E+01
28	tanespimycin	1.12E-01
97	furazolidone	7.51E+01
0	thiamazole	1.04E+02
100	BW-B70C	2.87E-09
16	HC_toxin	4.59E+00
88	clenbuterol	6.01E+01
100	albendazole	7.95E-10
40	pimethixene	9.25E-04
46	isoetarine	6.69E+01
5	cefazolin	2.12E+01
13	hydrastine_hydrochloride	7.09E+01
3	triamterene	1.00E+02
84	cefmetazole	5.49E+01
100	maprotiline	4.43E-10
100	phenazopyridine	8.22E-11
34	trimipramine	6.97E-01
13	flufenamic_acid	4.85E+01
76	nomifensine	5.60E+01
100	raloxifene	2.57E-11
7	triprolidine	2.79E+01
100	thioproperazine	5.38E-12
41	allantoin	3.94E+01
100	hexetidine	1.09E-12
74	ketorolac	9.46E+01
5	haloperidol	1.23E+01
92	beclometasone	3.45E+01
103	methoxsalen	4.00E+01
12	oxolamine	7.14E+01
6	corticosterone	8.45E+01
13	DL-thiorphan	3.21E+01
104	STOCK1N-35215	1.53E-01
6	tacrine	5.90E+01
13	labetalol	1.67E+01
0	pirlindole	1.05E+02
6	diphemanil_metilsulfate	3.40E+01
51	butein	4.16E+01
59	halcinonide	8.29E+01
92	tetramisole	1.17E+01
0	cyclopentolate	1.03E+02
100	amphotericin_B	3.73E-12
32	proxymetacaine	8.00E+01
16	scriptaid	2.91E+00
51	HNMPA-(AM)3	7.03E+00
100	clozapine	1.09E-12

46	corbadrine	3.28E+01
85	ursodeoxycholic_acid	5.23E+01
62	clioquinol	6.64E-03
89	cantharidin	4.71E+01
4	5114445	9.18E+01
104	F0447-0125	5.16E-02
64	pyrantel	2.02E+01
69	colchicine	6.95E+00
13	alpha-yohimbine	1.08E+01
97	tetroquinone	4.84E+01
3	quercetin	9.09E+01
7	5162773	2.42E+01
52	progesterone	6.22E+01
40	MG-262	5.20E-03
7	BAS-012416453	1.21E+01
90	sertaconazole	1.07E+02
61	cyclizine	1.05E+02
100	doxepin	3.75E-12
3	ciclopirox	7.06E+01
90	colecalciferol	1.07E+02
89	6-benzylaminopurine	3.67E+01
71	tropine	7.91E+01
38	atractyloside	7.50E+00
7	amrinone	6.08E+00
1	tamoxifen	7.37E+01
16	LY-294002	1.45E+00
5	quinidine	1.26E+01
37	bumetanide	9.70E+01
71	Prestwick-1085	3.92E+01
100	amoxapine	4.16E-12
52	cinchocaine	5.00E+01
100	tetrandrine	8.53E-13
32	lysergol	7.97E+01
6	naltrexone	2.77E+01
102	rolipram	6.09E+00
100	tonzonium_bromide	2.37E-13
71	methanthelinium_bromide	1.32E+01
34	spiperone	1.09E+00
100	thioridazine	5.41E-14
50	flecainide	8.22E+01
102	hydrocortisone	2.03E+00
59	mepenzolate_bromide	5.69E+01
102	nicotinic_acid	5.16E-01
51	dimethyloxalylglycine	7.38E-01
5	cinchonidine	7.59E+00
90	parbendazole	1.07E+02
37	enalapril	7.56E+01
5	bisoprolol	3.10E+00
62	rottlerin	1.49E-02
89	talampicillin	3.31E+01
100	cyclobenzaprine	5.65E-14
13	papaverine	1.07E+01
37	dimenhydrinate	4.65E+01
100	alimemazine	1.24E-14

60	gefitinib	1.10E+01
100	amitriptyline	2.20E-15
40	PF-00875133-00	1.27E-02
0	SC-560	1.06E+02
100	perhexiline	4.50E-16
104	5155877	5.50E-02
100	CP-645525-01	7.37E-17
42	clindamycin	1.88E+01
61	oxymetazoline	1.01E+02
32	tinidazole	7.75E+01
97	(+/-)-catechin	3.00E+01
61	propafenone	8.76E+01
40	mianserin	5.07E-03
60	nimesulide	4.92E+00
29	cinchonine	1.06E+02
102	naftidrofuryl	2.19E-01
12	SR-95639A	4.87E+01
105	gelsemine	7.46E+01
16	rifabutin	1.07E+00
105	diflorasone	3.12E+01
100	zuclopenthixol	1.01E-16
100	clofilium_tosylate	1.33E-17
40	dicycloverine	1.93E-03
100	orphenadrine	1.98E-18
4	berberine	9.04E+01
0	piretanide	1.06E+02
74	morantel	9.07E+01
5	streptomycin	2.47E+00
104	myricetin	1.87E-02
89	flumequine	3.18E+01
100	clomifene	7.11E-19
5	oxantel	9.06E-01
40	5253409	7.02E-04
61	nisoxetine	7.39E+01
90	tyloxapol	1.07E+02
16	mepacrine	3.69E-01
59	pempidine	3.58E+01
4	ikarugamycin	7.51E+01
42	ajmaline	1.11E+01
102	fluvastatin	7.86E-02
22	Prestwick-1100	8.58E+01
5	betamethasone	3.52E-01
61	Trolox_C	5.44E+01
95	tiaprofenic_acid	9.95E+01
7	5213008	1.11E+01
37	chenodeoxycholic_acid	3.22E+01
104	oxyphenbutazone	4.79E-03
32	amoxicillin	7.84E+01
13	gramine	1.19E+01
98	tretinoin	7.08E+01
58	meprylcaine	1.06E+02
15	STOCK1N-35874	2.59E+01
20	proglumide	4.71E+01
67	gibberellic_acid	7.85E+01

53	cicloheximide	1.65E+01
90	bretylium_tosilate	1.07E+02
61	fipexide	3.75E+01
4	decitabine	5.96E+01
89	pargyline	2.86E+01
31	corynanthine	1.06E+02
0	cefoperazone	1.07E+02
87	risperidone	9.23E+01
42	clopamide	5.57E+00
52	Prestwick-860	6.87E+01
61	mesoridazine	2.22E+01
50	benfotiamine	5.92E+01
61	triflusal	1.07E+01
62	clotrimazole	7.81E-02
54	esculetin	8.19E+01
62	miconazole	2.16E-02
72	3-acetamidocoumarin	1.03E+02
83	clidinium_bromide	9.33E+01
3	etoposide	8.05E+01
62	naftifine	5.81E-03
5	AG-028671	2.57E-01
52	ioversol	5.57E+01
64	aztreonam	1.19E+01
82	scopolamine_N-oxide	1.02E+02
13	tremorine	9.13E+00
50	spiramycin	2.82E+01
26	guanabenz	5.25E+01
11	CP-690334-01	9.42E+01
67	mephentermine	5.46E+01
89	sitosterol	2.44E+01
40	lynestrenol	2.31E-03
100	metergoline	7.28E-16
34	aminophenazone	2.91E+00
49	4,5-dianilinophthalimide	9.31E+01
62	foliosidine	2.34E-03
90	etanidazole	1.07E+02
30	amiprilose	7.64E+01
91	sulindac	8.47E+01
66	isosorbide	7.66E+01
16	bufexamac	3.16E-01
0	benzbromarone	1.07E+02
100	ivermectin	3.86E-16
52	palmatine	4.66E+01
100	co-dergocrine_mesilate	5.86E-17
102	florfenicol	6.77E-02
22	quinethazone	7.32E+01
74	meticrane	8.43E+01
90	dexpropranolol	1.07E+02
0	lidocaine	1.07E+02
18	valdecoxib	6.68E+01
97	paromomycin	2.39E+01
1	fasudil	5.73E+01
101	kawain	5.74E+01
58	meclofenoxate	1.05E+02

11	alpha-ergocryptine	6.76E+01
84	leflunomide	4.66E+01
40	dyclonine	1.81E-03
46	zomepirac	3.39E+01
42	danazol	3.91E+00
68	carbarsone	1.03E+02
13	diclofenac	7.67E+00
99	trimethylcolchicinic_acid	1.06E+02
0	ampicillin	1.07E+02
66	karakoline	3.51E+01
24	splitomicin	6.87E+01
62	mifepristone	1.84E-03
58	phenformin	9.97E+01
4	5286656	5.91E+01
74	imipenem	6.66E+01
85	milrinone	6.42E+01
5	bergenin	2.91E-01
99	trichlormethiazide	1.02E+02
35	gliquidone	1.02E+02
11	citalopram	3.69E+01
52	meropenem	4.25E+01
14	gallamine_triethiodide	1.03E+02
24	docosahexaenoic_acid_ethyl_ester	2.29E+01
95	moxonidine	9.10E+01
103	dicloxacillin	2.31E+01
38	guanadrel	7.58E+00
89	R-atenolol	2.91E+01
58	Prestwick-685	9.02E+01
90	liothyronine	1.07E+02
85	benzylpenicillin	3.86E+01
71	chloropyrazine	1.41E+01
27	isoflupredone	1.07E+02
85	sulfadoxine	1.72E+01
3	blebbistatin	7.94E+01
57	eticlopride	9.01E+01
50	ethoxyquin	1.46E+01
6	calycanthine	6.02E+01
0	diloxanide	1.07E+02
61	gliclazide	1.44E+01
52	ethaverine	3.39E+01
40	cyproheptadine	2.37E-03
42	chlorhexidine	2.12E+00
0	naphazoline	1.06E+02
101	clemizole	3.03E+01
95	trimethadione	7.04E+01
63	bisacodyl	3.09E-04
48	paclitaxel	1.04E+02
0	alclometasone	1.06E+02
61	thiocolchicoside	7.32E+00
32	flunixin	9.84E+01
32	luteolin	9.11E+01
92	iocetamic_acid	2.49E+01
5	convolamine	2.12E-01
39	drofenine	9.18E+01

102	(-)-atenolol	5.71E-02
100	ursolic_acid	6.99E-14
46	ebselen	1.71E+01
90	acepromazine	1.07E+02
90	nitrofurantoin	1.07E+02
22	nialamide	6.30E+01
5	noretynodrel	6.66E-02
4	artemisinin	5.38E+01
85	oxprenolol	7.67E+00
0	mimosine	1.06E+02
13	methacholine_chloride	9.12E+00
3	resveratrol	6.88E+01
66	betonicine	1.18E+01
49	rofecoxib	8.77E+01
7	Prestwick-1082	3.26E+01
58	3-acetylcoumarin	8.28E+01
8	pronetalol	1.06E+02
100	thiethylperazine	6.86E-14
89	hydrocotarnine	3.19E+01
13	sulfadiazine	4.75E+00
21	mephenesin	9.81E+01
62	erastin	5.22E-03
1	sodium_phenylbutyrate	3.56E+01
75	ribavirin	8.68E+01
5	5140203	2.53E-02
19	orlistat	1.07E+02
59	eldeline	4.00E+01
92	ethionamide	1.05E+01
1	2-deoxy-D-glucose	1.19E+01
34	dihydroergotamine	5.72E+00
0	baclofen	1.06E+02
25	benzonatate	9.75E+01
2	bucladesine	8.77E+01
74	fenspiride	6.13E+01
59	flunisolide	1.81E+01
13	protoveratrine_A	2.60E+00
88	ritodrine	7.30E+01
7	5151277	2.48E+01
13	ifosfamide	9.97E-01
0	sulfasalazine	1.05E+02
59	solasodine	5.96E+00
0	chloropyramine	1.04E+02
36	gabexate	9.94E+01
71	ondansetron	6.33E+00
67	Prestwick-967	5.40E+01
7	cefapirin	1.50E+01
104	carmustine	7.57E-02
4	pinacidil	4.63E+01
3	5109870	5.94E+01
97	iodixanol	2.03E+01
7	cefotetan	7.98E+00
41	roxarsone	7.39E+01
90	pentamidine	1.07E+02
34	penbutolol	2.96E+00

3	guaifenesin	4.11E+01
7	cinnarizine	3.79E+00
0	N6-methyladenosine	1.04E+02
20	indapamide	4.14E+01
6	5149715	6.24E+01
90	sulconazole	1.07E+02
6	metformin	4.28E+01
106	molindone	7.57E+01
89	oxytetracycline	3.39E+01
106	meptazinol	4.76E+01
97	amylocaine	7.56E+00
32	paroxetine	9.79E+01
90	Prestwick-674	1.07E+02
26	aciclovir	4.31E+01
106	alprostadil	2.31E+01
60	famotidine	3.03E+01
52	cefsulodin	4.96E+01
12	meclocycline	5.67E+01
72	decamethonium_bromide	1.03E+02
34	isoconazole	1.30E+00
62	azacyclonol	8.05E-03
88	ketoconazole	4.44E+01
6	5152487	2.90E+01
34	rescinnamine	3.63E-01
92	Prestwick-642	4.22E+00
62	butoconazole	1.93E-03
3	5211181	3.01E+01
11	dilazep	2.79E+01
92	vitexin	7.06E-01
55	napelline	6.85E+01
29	vigabatrin	1.07E+02
3	deferoxamine	1.68E+01
14	MS-275	1.04E+02
19	STOCK1N-35696	1.07E+02
104	lomustine	3.30E-02
37	dropropizine	5.74E+01
59	alcuronium_chloride	2.33E+00
0	brinzolamide	1.05E+02
80	iproniazid	9.83E+01
99	fosfosal	1.02E+02
34	atropine_oxide	1.10E-01
7	melatonin	3.19E+00
13	testosterone	1.21E+00
100	norcyclobenzaprine	8.36E-11
37	telenzepine	3.52E+01
99	simvastatin	9.36E+01
49	troleandomycin	8.29E+01
90	clorgiline	1.07E+02
89	tolbutamide	3.44E+01
39	isopropamide_iodide	7.13E+01
32	acacetin	9.84E+01
62	rotenone	9.36E-04
0	Prestwick-857	1.05E+02
40	propidium_iodide	6.69E-02

32	2,6-dimethylpiperidine	9.25E+01
89	picrotoxinin	2.41E+01
82	remoxipride	1.03E+02
58	sulfamethoxypyridazine	8.69E+01
58	aminohippuric_acid	7.01E+01
90	sulfamerazine	1.07E+02
6	tetrahydroalstonine	2.25E+01
41	octopamine	5.83E+01
77	nitrendipine	9.42E+01
66	pramocaine	3.08E+00
32	etidronic_acid	8.62E+01
27	tolnaftate	1.07E+02
35	metrizamide	1.01E+02
103	velnacrine	7.66E+00
84	nadide	3.70E+01
74	acetohexamide	6.07E+01
62	ticlopidine	3.10E-04
78	oligomycin	8.60E+01
5	minocycline	1.03E-01
100	monensin	2.74E-10
100	raubasine	4.74E-11
92	bicuculline	1.04E-01
67	cefalonium	4.54E+01
42	Prestwick-689	5.30E+00
100	proguanil	1.03E-11
102	flurbiprofen	1.85E-01
22	santonin	6.77E+01
54	halofantrine	7.57E+01
32	tiabendazole	8.12E+01
7	ricinine	2.72E+00
82	nabumetone	9.37E+01
106	Prestwick-983	1.44E+01
19	mebeverine	1.07E+02
41	torasemide	3.57E+01
90	riboflavin	1.07E+02
74	naproxen	4.28E+01
34	dosulepin	6.04E-02
32	heliotrine	7.25E+01
87	moracizine	9.45E+01
13	scopolamine	1.20E+00
32	thioguanosine	6.03E+01
74	nipecotic_acid	2.42E+01
90	cycloserine	1.07E+02
90	cyproterone	1.07E+02
57	vancomycin	7.79E+01
10	15(S)-15-methylprostaglandin_E2	9.65E+01
19	glafenine	1.07E+02
32	trioxysalen	4.94E+01
81	epiandrosterone	7.84E+01
7	tiratricol	1.48E+00
64	oxolinic_acid	1.33E+01
43	estrone	1.06E+02
52	esculin	6.67E+01
49	verapamil	7.35E+01

60	amodiaquine	3.50E+01
62	LM-1685	2.04E-04
69	oxedrine	5.26E+01
61	picotamide	2.63E+01
50	dicoumarol	1.63E+01
7	benzydamine	5.95E-01
90	medrysone	1.07E+02
21	0173570-0000	8.99E+01
84	ramifenazone	1.43E+01
87	lansoprazole	7.40E+01
3	colforsin	2.07E+01
27	Prestwick-692	1.07E+02
90	latamoxef	1.07E+02
40	dienestrol	1.74E-01
59	citiolone	1.08E+00
90	apramycin	1.07E+02
12	brompheniramine	4.40E+01
25	acetazolamide	9.76E+01
19	ornidazole	1.07E+02
0	glimepiride	1.07E+02
37	prednisolone	3.09E+01
52	natamycin	5.94E+01
32	metyrapone	4.76E+01
14	mitoxantrone	1.04E+02
0	nifenazone	1.07E+02
74	hydralazine	1.54E+01
61	iobenguane	1.76E+01
60	SC-58125	2.39E+01
105	conessine	4.71E+01
8	ethambutol	1.06E+02
34	proadifen	2.65E-02
6	5186223	2.52E+01
90	capsaicin	1.07E+02
102	urapidil	1.04E-01
94	tioguanine	9.14E+01
14	azacitidine	9.78E+01
25	moxisylyte	8.39E+01
32	skimmianine	4.02E+01
60	chlorphenesin	1.28E+01
19	erythromycin	1.07E+02
39	carbinoxamine	4.90E+01
90	adrenosterone	1.07E+02
61	ceftazidime	1.00E+01
47	sparteine	1.05E+02
40	isotretinoin	1.37E-01
90	alverine	1.07E+02
31	cefotaxime	1.07E+02
32	dextromethorphan	3.14E+01
52	pivampicillin	5.35E+01
87	spaglumic_acid	4.98E+01
29	furosemide	1.07E+02
54	Prestwick-981	5.07E+01
88	procainamide	3.41E+01
70	vinblastine	9.96E+01

90	sulfinpyrazone	1.07E+02
16	vorinostat	9.45E+00
99	colistin	9.61E+01
0	sulfanilamide	1.07E+02
62	mercaptopurine	2.34E-04
90	hexestrol	1.07E+02
17	thalidomide	9.31E+01
89	etamsylate	5.03E+01
93	flutamide	1.05E+02
14	GW-8510	9.00E+01
13	physostigmine	2.00E+00
32	vidarabine	2.61E+01
6	etodolac	1.84E+01
12	Chicago_Sky_Blue_6B	2.38E+01
73	methylergometrine	5.32E+01
35	articaine	9.74E+01
25	dexpanthenol	6.68E+01
52	piromidic_acid	4.62E+01
19	16,16-dimethylprostaglandin_E2	1.07E+02
58	tetryzoline	8.22E+01
7	mecamylamine	8.08E-01
29	iopamidol	1.07E+02
90	pipenzolate_bromide	1.07E+02
84	butamben	3.51E+00
62	isradipine	5.08E-05
7	piroxicam	2.51E-01
4	thiamine	8.27E+01
35	aminocaproic_acid	8.17E+01
0	propoxycaine	1.07E+02
90	anabasine	1.07E+02
13	demeclocycline	9.94E-01
0	ergocalciferol	1.07E+02
58	retrorsine	6.80E+01
0	dexamethasone	1.07E+02
29	primidone	1.07E+02
44	cobalt_chloride	1.01E+02
90	norethisterone	1.07E+02
13	cefuroxime	3.33E-01
94	W-13	5.63E+01
0	hemicholinium	1.06E+02
52	fluorocurarine	3.91E+01
89	verteporfin	4.82E+01
90	clobetasol	1.06E+02
87	prednicarbate	2.80E+01
100	flupentixol	1.89E-07
11	spiradoline	2.83E+01
90	scopoletin	1.06E+02
52	xylazine	2.74E+01
100	sirolimus	4.79E-08
83	iopromide	1.02E+02
20	sulfachlorpyridazine	4.22E+01
52	betahistine	1.71E+01
100	5666823	1.12E-08
31	idazoxan	1.07E+02

75	rifampicin	7.82E+01
93	ofloxacin	9.81E+01
0	demecarium_bromide	1.06E+02
77	paracetamol	7.85E+01
88	domperidone	1.52E+01
43	pizotifen	1.06E+02
85	estriol	2.49E+01
85	etomidate	8.87E+00
32	rimexolone	3.22E+01
32	acetylsalicylic_acid	2.14E+01
0	0225151-0000	1.06E+02
90	tranylcypromine	1.06E+02
0	stachydrine	1.05E+02
74	norfloxacin	1.57E+01
52	dimethadione	1.18E+01
44	dopamine	7.99E+01
17	NU-1025	6.02E+01
37	niflumic_acid	3.05E+01
74	phenindione	6.01E+00
85	azaperone	2.24E+00
73	alfuzosin	3.73E+01
67	sulfaquinoxaline	5.27E+01
29	metronidazole	1.07E+02
29	trihexyphenidyl	1.06E+02
42	betaxolol	1.24E+01
31	doxylamine	1.07E+02
22	ciclacillin	7.90E+01
14	daunorubicin	8.77E+01
48	chloroquine	1.06E+02
91	exisulind	9.32E+01
79	ozagrel	1.05E+02
95	propylthiouracil	9.66E+01
31	pralidoxime	1.06E+02
61	budesonide	1.47E+01
32	lymecycline	1.89E+01
38	sulfadimethoxine	1.83E+01
19	AH-6809	1.07E+02
106	etilefrine	1.71E+01
89	phentolamine	5.27E+01
32	metacycline	1.21E+01
41	imidurea	4.11E+01
50	piperacillin	1.20E+01
22	homosalate	5.99E+01
90	famprofazone	1.06E+02
39	butirosin	2.95E+01
100	lasalocid	8.56E-08
76	neomycin	9.45E+01
30	Prestwick-864	8.33E+01
16	trichostatin_A	1.13E+01
67	indoprofen	3.23E+01
76	acetylsalicylsalicylic_acid	6.69E+01
106	zalcitabine	5.13E+00
64	hyoscyamine	6.35E+00
32	calcium_pantothenate	8.43E+00

2	3-aminobenzamide	8.41E+01
41	niridazole	1.94E+01
22	levcycloserine	3.86E+01
49	mesalazine	8.46E+01
31	gemfibrozil	1.05E+02
0	sulfabenzamide	1.06E+02
61	tubocurarine_chloride	8.96E+00
9	IC-86621	1.06E+02
0	etynodiol	1.06E+02
96	sulfaguanidine	1.06E+02
32	ethotoin	5.31E+00
0	chlorpropamide	1.05E+02
81	pirenperone	6.93E+01
72	memantine	1.06E+02
0	pregnenolone	1.04E+02
31	oxetacaine	1.02E+02
25	triflupromazine	6.31E+01
89	sotalol	5.03E+01
6	prednisone	2.78E+01
56	dantrolene	9.94E+01
57	2-aminobenzenesulfonamide	7.04E+01
90	fluorometholone	1.07E+02
19	AH-23848	1.07E+02
22	chlorambucil	2.14E+01
60	vinpocetine	2.49E+01
38	khellin	4.47E+00
35	alfadolone	7.64E+01
34	nicergoline	1.06E-01
25	metrifonate	3.87E+01
93	nalidixic_acid	8.68E+01
90	denatonium_benzoate	1.07E+02
20	PHA-00851261E	2.40E+01
62	antimycin_A	3.26E-04
48	demecolcine	1.04E+02
2	4-hydroxyphenazone	5.02E+01
90	Prestwick-1084	1.06E+02
20	0317956-0000	4.86E+00
100	pimozide	5.10E-07
90	flunarizine	1.06E+02
3	trifluridine	5.23E+01
0	cromoglicic_acid	1.05E+02
82	Prestwick-1083	1.01E+02
87	bethanechol	1.73E+01
0	pentetic_acid	1.04E+02
5	azlocillin	4.52E+00
61	etamivan	5.83E+00
7	noscapine	1.12E+00
14	fisetin	8.60E+01
46	pridinol	4.66E+01
0	pheniramine	1.03E+02
40	vinburnine	3.34E+00
31	levocabastine	9.87E+01
101	pentolonium	6.06E+01
52	crotamiton	2.22E+01

35	Prestwick-972	5.48E+01
18	3-nitropropionic_acid	7.13E+01
7	diflunisal	3.51E-01
79	asiaticoside	9.88E+01
35	tiletamine	2.78E+01
40	nalbuphine	1.63E+00
61	Prestwick-559	2.35E+00
85	repaglinide	8.41E-01
90	difenidol	1.06E+02
48	12,13-EODE	9.85E+01
40	ionomycin	6.44E-01
89	altizide	5.36E+01
27	adiphenine	1.07E+02
29	enoxacin	1.06E+02
99	galantamine	1.03E+02
0	cytisine	1.03E+02
6	AR-A014418	2.35E+01
16	idoxuridine	9.98E+00
89	zimeldine	4.10E+01
90	ranitidine	1.06E+02
55	kinetin	7.80E+01
35	enilconazole	1.02E+01
0	ascorbic_acid	1.02E+02
32	sulfametoxydiazine	9.59E+00
55	estropipate	4.18E+01
32	harman	4.97E+00
86	flucloxacillin	9.16E+01
45	tetraethylenepentamine	1.02E+02
67	epitiostanol	2.42E+01
6	hexylcaine	1.26E+01
100	promazine	2.81E-06
80	mefexamide	1.00E+02
26	timolol	6.63E+01
90	ethosuximide	1.06E+02
61	trimetazidine	9.37E-01
43	equilin	1.06E+02
72	fluticasone	1.05E+02
96	succinylsulfathiazole	1.01E+02
99	reserpine	9.79E+01
27	isoxicam	1.07E+02
95	seneciphylline	9.60E+01
67	tocainide	8.41E+00
34	chlorphenamine	3.64E-02
0	nizatidine	1.03E+02
89	8-azaguanine	3.58E+01
31	sulfathiazole	9.59E+01
10	CP-319743	9.32E+01
96	minoxidil	8.15E+01
28	fulvestrant	1.22E+01
77	oxybenzone	6.01E+01
41	chloramphenicol	1.29E+01
76	pirenzepine	4.60E+01
86	rilmenidine	6.05E+01
5	methocarbamol	4.59E+00

90	trazodone	1.06E+02
27	isometheptene	1.07E+02
82	iopanoic_acid	9.75E+01
47	bacampicillin	1.05E+02
37	fenofibrate	3.88E+01
99	mestranol	8.91E+01
27	carbimazole	1.06E+02
25	atracurium_besilate	2.99E+01
4	monocrotaline	1.02E+02
6	estradiol	7.38E+00
102	meglumine	1.26E+00
90	bezafibrate	1.06E+02
49	racecadotril	8.72E+01
11	CP-320650-01	2.74E+01
15	diclofenamide	6.29E+01
40	Y-27632	9.16E-01
58	procyclidine	9.54E+01
59	fludroxycortide	2.73E+00
22	Prestwick-1080	1.93E+01
53	emetine	7.30E+01
106	naftopidil	2.81E+00
25	sulfacetamide	1.14E+01
67	ticarcillin	1.92E+00
6	debrisoquine	2.71E+00
1	novobiocin	3.86E+01
13	tomatidine	5.39E+00
78	clofibrate	8.02E+01
100	lovastatin	1.77E-05
71	ciprofibrate	2.97E+01
2	probucol	2.22E+01
73	hesperidin	4.28E+01
41	Prestwick-920	3.15E+00
90	omeprazole	1.06E+02
32	nifedipine	8.14E+00
0	PF-00539758-00	1.05E+02
81	propranolol	5.21E+01
5	pivmecillinam	2.84E+00
52	doxazosin	3.69E+01
27	clorsulon	1.06E+02
58	benzthiazide	8.79E+01
90	piperidolate	1.06E+02
29	cefotiam	1.07E+02
80	pentoxifylline	8.74E+01
32	sulfaphenazole	4.66E+00
68	carcinine	1.07E+02
90	(-)-MK-801	1.05E+02
90	ronidazole	1.04E+02
37	acemetacin	2.42E+01
8	probenecid	1.07E+02
17	monastrol	3.52E+01
100	procarbazine	1.43E-05
52	rolitetracycline	2.65E+01
104	semustine	7.24E+00
27	thiamphenicol	1.04E+02

80	cloxacillin	5.53E+01
101	riluzole	4.38E+01
12	carisoprodol	3.43E+01
58	flumetasone	7.62E+01
102	dioxybenzone	3.22E-01
58	fenoprofen	5.65E+01
54	bacitracin	5.63E+01
7	triamcinolone	9.02E-01
60	bambuterol	4.17E+01
45	oxamic_acid	8.41E+01
1	arachidonyltrifluoromethane	1.28E+01
68	lactobionic_acid	1.07E+02
68	guanfacine	1.06E+02
0	antazoline	1.06E+02
24	U0125	3.75E+01
105	yohimbic_acid	5.78E+01
8	sisomicin	1.07E+02
27	ketotifen	1.02E+02
104	phenoxybenzamine	2.36E+00
29	canadine	1.06E+02
89	desoxycortone	5.33E+01
100	troglitazone	1.21E-05
43	saquinavir	1.05E+02
54	trapidil	1.95E+01
90	pentoxyverine	1.06E+02
19	PHA-00767505E	1.07E+02
19	lobelanidine	1.07E+02
8	fusaric_acid	1.05E+02
52	cinoxacin	2.21E+01
90	ganciclovir	1.05E+02
45	copper_sulfate	3.95E+01
4	carbamazepine	1.04E+02
14	H-7	9.66E+01
52	dydrogesterone	1.20E+01
0	praziquantel	1.06E+02
3	5255229	7.91E+01
0	chlormezanone	1.06E+02
90	lorglumide	1.04E+02
40	econazole	1.91E+00
0	quipazine	1.05E+02
90	ifenprodil	1.03E+02
97	tolmetin	5.70E+01
32	todralazine	6.30E+00
99	theophylline	9.04E+01
13	ramipril	6.40E+00
69	nifuroxazide	9.58E+01
55	metoprolol	2.21E+01
89	ipratropium_bromide	4.80E+01
21	cefamandole	1.02E+02
90	tropicamide	1.01E+02
0	megestrol	1.05E+02
0	harmalol	1.03E+02
48	5252917	1.00E+02
4	canrenoic_acid	1.01E+02

0	10-methoxyharmalan	1.01E+02
43	naringenin	1.01E+02
90	mevalolactone	9.98E+01
4	molsidomine	9.36E+01
82	flavoxate	9.72E+01
0	PF-00539745-00	9.90E+01
13	bupropion	2.61E+00
23	SB-203580	1.01E+02
90	cetirizine	9.74E+01
19	zaprinast	1.07E+02
21	PNU-0230031	8.78E+01
35	atropine_methonitrate	7.41E+00
7	zidovudine	6.12E-01
90	meclozine	9.40E+01
47	cyanocobalamin	1.03E+02
78	arachidonic_acid	4.46E+01
0	gentamicin	9.77E+01
40	PHA-00846566E	1.30E+00
49	kanamycin	9.01E+01
53	cephaeline	5.72E+01
42	methazolamide	4.97E+01
52	lomefloxacin	1.06E+01
100	S-propranolol	7.31E-05
19	sulfapyridine	1.07E+02
52	bemegride	4.26E+00
0	hymecromone	9.57E+01
36	nystatin	1.07E+02
14	ellipticine	9.27E+01
0	lincomycin	9.10E+01
0	netilmicin	8.40E+01
3	5248896	7.50E+01
0	tetracaine	7.61E+01
33	clebopride	1.07E+02
8	phenacetin	1.04E+02
19	cotinine	1.07E+02
99	arcaine	8.32E+01
0	cefepime	6.89E+01
90	cortisone	9.69E+01
49	alpha-estradiol	7.43E+01
3	5279552	5.66E+01
44	quinpirole	8.19E+01
4	metanephrine	8.96E+01
89	methyldopa	5.21E+01
50	sulfadimidine	1.69E+01
16	fusidic_acid	2.65E+01
87	ioxaglic_acid	1.71E+01
73	diltiazem	3.77E+01
61	piracetam	4.03E+00
82	procaine	8.84E+01
33	prazosin	1.07E+02
104	5194442	1.33E+00
5	levothyroxine_sodium	7.12E+00
68	pepstatin	1.06E+02
27	viomycin	1.04E+02

80	meclofenamic_acid	3.02E+01
31	isoxsuprine	1.05E+02
37	diethylcarbamazine	2.33E+01
77	nimodipine	3.94E+01
72	harpagoside	1.07E+02
90	naloxone	9.89E+01
43	MK-886	9.51E+01
19	AG-012559	1.07E+02
98	TTNPB	1.03E+02
100	fluvoxamine	1.43E-04
74	proxyphylline	4.18E+01
90	prasterone	9.61E+01
101	trimethoprim	2.52E+01
32	chrysin	1.39E+01
29	amikacin	1.07E+02
0	midodrine	7.94E+01
90	methapyrilene	9.23E+01
48	(-)-catechin	9.64E+01
22	lithocholic_acid	2.65E+01
71	mephenytoin	2.07E+01
90	aminophylline	8.70E+01
43	ciclosporin	7.70E+01
31	minaprine	1.02E+02
91	sulindac_sulfide	1.00E+02
0	folic_acid	7.49E+01
48	DL-PPMP	7.79E+01
4	pyridoxine	8.51E+01
32	pancuronium_bromide	8.34E+00
95	selegiline	1.03E+02
90	furaltadone	8.34E+01
89	dacarbazine	5.34E+01
19	0198306-0000	1.07E+02
39	ribostamycin	3.53E+01
90	phthalylsulfathiazole	7.63E+01
89	laudanosine	3.65E+01
79	heptaminol	1.01E+02
52	edrophonium_chloride	5.12E+00
81	biperiden	3.61E+01
90	etofenamate	6.88E+01
11	PHA-00745360	2.37E+01
65	pirinixic_acid	1.06E+02
89	ginkgolide_A	2.26E+01
12	hydrochlorothiazide	2.42E+01
32	apigenin	4.60E+00
29	ceforanide	1.07E+02
90	zoxazolamine	6.21E+01
25	mefenamic_acid	1.32E+01
99	spectinomycin	7.94E+01
90	methylprednisolone	5.18E+01
0	captopril	8.05E+01
14	alsterpaullone	9.35E+01
100	diphenylpyraline	2.39E-04
93	tiapride	9.85E+01
74	roxithromycin	2.61E+01

31	amprolium	9.94E+01
19	dipivefrine	1.07E+02
97	tenoxicam	5.32E+01
83	cefoxitin	1.07E+02
91	phenyl_biguanide	8.35E+01
76	acenocoumarol	4.01E+01
31	cimetidine	8.90E+01
69	amiloride	9.82E+01
29	nitrofural	1.06E+02
37	bupivacaine	1.05E+01
46	gabapentin	6.94E+01
27	dapsone	1.04E+02
21	PNU-0293363	7.73E+01
19	pseudopelletierine	1.07E+02
60	hexamethonium_bromide	7.18E+01
0	streptozocin	8.30E+01
52	naringin	2.76E+00
16	rosiglitazone	2.34E+01
62	tribenoside	4.00E-01
75	mycophenolic_acid	9.28E+01
0	tridihexethyl	7.59E+01
0	dihydrostreptomycin	6.45E+01
19	CAY-10397	1.07E+02
4	hydroflumethiazide	8.34E+01
69	(+)-isoprenaline	8.40E+01
5	topiramate	8.34E+00
49	midecamycin	7.43E+01
0	sulfamethizole	5.61E+01
4	bromperidol	6.39E+01
19	hesperetin	1.07E+02
0	cyclic_adenosine_monophosphate	4.48E+01
13	lidoflazine	8.03E+00
0	dizocilpine	3.31E+01
18	PF-01378883-00	6.42E+01
9	16-phenyltetranorprostaglandin_E2	1.07E+02
33	nomegestrol	1.06E+02
27	etiocholanolone	1.02E+02
97	carbenoxolone	2.37E+01
31	salsolidin	7.98E+01
99	atovaquone	6.84E+01
65	celecoxib	1.00E+02
79	betulin	8.93E+01
8	oxamniquine	1.05E+02
86	azapropazone	5.61E+01
73	dexibuprofen	2.51E+01
74	lisinopril	1.13E+01
3	kaempferol	7.49E+01
27	merbromin	9.54E+01
19	dehydrocholic_acid	1.07E+02
36	levopropoxyphene	1.06E+02
60	acebutolol	5.88E+01
90	eucatropine	8.28E+01
93	griseofulvin	8.51E+01
0	loracarbef	3.54E+01

0	pindolol	2.36E+01
90	fluocinonide	7.45E+01
27	nadolol	8.20E+01
4	quinostatin	4.95E+01
14	camptothecin	9.21E+01
82	alfaxalone	9.14E+01
90	amiodarone	6.57E+01
60	guanethidine	3.29E+01
42	ampyrone	7.20E+01
14	doxorubicin	7.23E+01
53	vanoxerine	4.54E+01
23	SC-19220	9.77E+01
0	aconitine	1.96E+01
33	coralyne	1.04E+02
91	tacrolimus	5.33E+01
0	isocorydine	1.17E+01
29	tranexamic_acid	1.07E+02
8	tolazamide	1.03E+02
100	fluoxetine	2.57E-03
40	tyrphostin_AG-1478	1.73E+01
96	tolazoline	9.41E+01
89	solanine	4.23E+01
30	suprofen	9.87E+01
5	aminoglutethimide	4.45E+00
72	benzathine_benzylpenicillin	1.07E+02
27	homatropine	6.67E+01
29	boldine	1.06E+02
90	promethazine	7.01E+01
89	tracazolate	2.42E+01
19	propantheline_bromide	1.07E+02
26	thioperamide	9.09E+01
95	tobramycin	1.05E+02
61	oleandomycin	8.89E+00
29	fludrocortisone	1.04E+02
19	caffeic_acid	1.07E+02
0	yohimbine	1.16E+01
90	pyrazinamide	6.40E+01
90	oxybuprocaine	4.95E+01
32	harmol	1.82E+01
47	cefixime	1.05E+02
31	phenazone	7.65E+01
16	valproic_acid	1.66E+01
94	benserazide	7.58E+01
49	dexverapamil	6.67E+01
44	indometacin	6.80E+01
32	harmine	7.26E+00
6	Prestwick-675	4.15E+01
42	hydrastinine	5.60E+01
19	deptropine	1.07E+02
6	aceclofenac	1.33E+01
83	pyrithyldione	1.07E+02
72	methyldopate	1.07E+02
82	piribedil	7.84E+01
9	11-deoxy-16,16-	1.06E+02

	dimethylprostaglandin_E2	
49	mepyramine	3.03E+01
19	salsolinol	1.07E+02
56	vincamine	1.04E+02
0	Prestwick-1103	1.22E+01
68	dl-alpha_tocopherol	1.07E+02
99	theobromine	6.50E+01
19	pipemidic_acid	1.07E+02
83	chlortalidone	1.05E+02
48	3-hydroxy-DL-kynurenine	8.54E+01
96	cyclopenthiazide	6.58E+01
47	neostigmine_bromide	9.65E+01
82	phensuximide	4.11E+01
90	metamizole_sodium	6.20E+01
68	piperine	1.06E+02
0	myosmine	8.06E+00
36	Prestwick-682	1.06E+02
90	sulfafurazole	4.90E+01
68	mebhydrolin	1.02E+02
90	cefalexin	3.36E+01
69	dobutamine	8.50E+01
8	nafcillin	1.01E+02
29	isoniazid	1.04E+02
60	tetracycline	2.45E+01
72	methoxamine	1.06E+02
88	androsterone	6.44E+01
0	metampicillin	4.64E+00
8	6-azathymine	8.39E+01
79	diphenhydramine	7.10E+01
8	etifenin	4.37E+01
19	PF-00562151-00	1.07E+02
52	cisapride	9.59E+00
19	sulpiride	1.07E+02
15	pilocarpine	7.84E+01
83	sulfamonomethoxine	9.74E+01
13	hydroxyachillin	1.56E+01
58	levonorgestrel	1.06E+02
29	sulmazole	1.01E+02
29	ambroxol	8.74E+01
58	dorzolamide	1.04E+02
68	7-aminocephalosporanic_acid	9.10E+01
26	finasteride	6.97E+01
68	lumicolchicine	5.28E+01
99	canavanine	4.02E+01
72	iohexol	1.04E+02
31	pyrimethamine	7.40E+01
43	diethylstilbestrol	9.51E+01
48	phenanthridinone	5.88E+01
65	imatinib	8.79E+01
19	oxybutynin	1.07E+02
10	BCB000039	1.05E+02
58	buflomedil	9.71E+01
57	felbinac	1.04E+02
47	N-acetylmuramic_acid	7.40E+01

72	cefaclor	9.27E+01
19	dinoprostone	1.07E+02
3	apomorphine	9.73E+01
4	depudecin	7.34E+01
19	benfluorex	1.07E+02
69	nocodazole	6.06E+01
83	doxycycline	7.24E+01
19	0316684-0000	1.07E+02
36	benzocaine	1.03E+02
46	hecogenin	7.36E+01
0	N-phenylanthranilic_acid	8.96E+00
19	STOCK1N-28457	1.07E+02
19	glycocholic_acid	1.07E+02
9	BCB000038	1.05E+02
89	dipyridamole	5.58E+01
58	butyl_hydroxybenzoate	8.47E+01
7	5186324	3.21E+01
42	chlortetracycline	5.27E+01
19	SB-202190	1.07E+02
89	epivincamine	2.35E+01
95	L-methionine_sulfoximine	1.06E+02
19	genistein	1.07E+02
27	pheneticillin	8.85E+01
19	disopyramide	1.07E+02
90	nifurtimox	8.44E+01
4	butacaine	4.73E+01
3	hycanthone	8.91E+01
23	pioglitazone	9.15E+01
98	tomelukast	1.06E+02
19	PHA-00816795	1.07E+02
19	H-89	1.07E+02
105	Prestwick-691	8.95E+01
90	xylometazoline	7.64E+01
58	fenbufen	5.76E+01
56	ethisterone	9.68E+01
40	5230742	7.38E+01
29	mafenide	9.22E+01
90	ketanserin	6.01E+01
70	meteneprost	1.07E+02
19	levodopa	1.07E+02
57	xamoterol	9.27E+01
10	BCB000040	9.57E+01
40	primaquine	4.09E+01
90	monobenzone	4.07E+01
93	metaraminol	9.18E+01
22	Prestwick-984	7.94E+01
14	staurosporine	1.02E+02
72	fursultiamine	8.28E+01
21	PNU-0251126	9.35E+01
95	benperidol	1.05E+02
75	methotrexate	9.84E+01
98	iloprost	9.87E+01
31	SR-95531	7.69E+01
90	sulfamethoxazole	2.37E+01

19	AG-013608	1.07E+02
33	carteolol	1.07E+02
29	moroxydine	7.60E+01
19	metolazone	1.07E+02
19	practolol	1.07E+02
32	irinotecan	6.19E+01
43	betulinic_acid	9.51E+01
0	6-bromoindirubin-3-oxime	4.03E+01
19	glipizide	1.07E+02
19	adenosine_phosphate	1.07E+02
19	nilutamide	1.07E+02
3	5182598	8.97E+01
36	amantadine	1.02E+02
95	cefadroxil	9.91E+01
19	alprenolol	1.07E+02
33	arecoline	1.03E+02
9	CP-944629	1.04E+02
30	levobunolol	1.05E+02
27	chlorogenic_acid	9.98E+01
70	CP-863187	1.07E+02
19	PHA-00665752	1.07E+02
19	harmaline	9.91E+01
14	tyrphostin_AG-825	1.06E+02
70	suramin_sodium	1.07E+02

(b)

**Community**	**Drug**	**Enrichment P-val**
19	glipizide	4.01E+00
9	CP-944629	8.16E-01
0	6-bromoindirubin-3-oxime	1.78E+01
32	irinotecan	1.22E+01
70	CP-863187	1.63E+00
19	nilutamide	2.00E+00
14	tyrphostin_AG-825	8.31E+00
19	0198306-0000	2.59E-01
43	betulinic_acid	6.46E+00
19	adenosine_phosphate	3.31E-02
14	staurosporine	6.80E-01
19	AG-013608	4.16E-03
75	methotrexate	4.19E+00
19	harmaline	5.17E-04
19	alprenolol	3.66E-05
30	levobunolol	5.14E+00
19	STOCK1N-28457	4.34E-06
14	GW-8510	9.60E-02
33	arecoline	9.00E+00
3	apomorphine	2.73E+01
86	azapropazone	6.71E+00
72	methyldopate	1.83E+01
29	isoniazid	3.35E+01
29	moroxydine	5.86E+00

19	SB-202190	9.46E-06
19	practolol	7.62E-07
90	cefalexin	8.74E+01
70	suramin_sodium	2.76E-01
95	cefadroxil	2.15E+01
46	hecogenin	1.61E+01
90	ganciclovir	6.08E+01
4	5286656	4.20E+01
19	levodopa	7.12E-07
29	fludrocortisone	1.66E+00
31	phenazone	3.97E+01
19	salsolinol	1.29E-07
72	cefaclor	3.90E+00
19	PHA-00665752	1.57E-08
10	BCB000040	1.22E+01
29	enoxacin	3.29E-01
3	5248896	1.25E+01
58	benzthiazide	4.77E+01
89	epivincamine	7.20E+01
48	phenanthridinone	2.84E+01
70	meteneprost	1.59E-02
58	butyl_hydroxybenzoate	1.39E+01
68	dl-alpha_tocopherol	3.29E+01
19	H-89	3.12E-08
19	0316684-0000	2.81E-09
32	harmol	4.75E+01
83	chlortalidone	2.60E+01
90	sulfamethoxazole	6.63E+01
19	hesperetin	6.61E-10
19	metolazone	5.49E-11
90	naloxone	4.57E+01
27	chlorogenic_acid	5.85E+01
90	ketanserin	2.75E+01
12	hydrochlorothiazide	2.91E+01
105	Prestwick-691	2.21E+01
69	nocodazole	4.02E+01
72	iohexol	1.30E+00
19	sulpiride	4.63E-11
8	etifenin	4.49E+01
22	Prestwick-1080	4.85E+01
58	buflomedil	5.98E+00
98	iloprost	2.00E+01
27	adiphenine	2.51E+01
36	amantadine	2.94E+01
72	methoxamine	1.89E-01
32	calcium_pantothenate	3.71E+01
57	xamoterol	2.61E+01
3	5182598	8.78E+00
58	meprylcaine	1.61E+00
89	8-azaguanine	6.06E+01
19	oxybutynin	1.35E-10
77	paracetamol	2.28E+01
4	depudecin	3.54E+01
27	nadolol	9.31E+00

68	pepstatin	1.26E+01
4	quinostatin	1.27E+01
29	mafenide	7.96E-01
36	benzocaine	5.28E+00
0	metampicillin	1.03E+02
0	gentamicin	9.48E+01
90	etofenamate	4.36E+01
42	ampyrone	6.86E+01
91	exisulind	3.12E+01
47	N-acetylmuramic_acid	3.66E+01
68	guanfacine	2.75E+00
105	yohimbic_acid	4.36E+00
89	picrotoxinin	4.34E+01
37	acemetacin	6.25E+01
89	zimeldine	2.17E+01
72	fursultiamine	6.01E-02
93	metaraminol	3.90E+01
90	monobenzone	3.83E+01
0	sulfasalazine	8.98E+01
21	PNU-0251126	4.00E+01
95	benperidol	1.83E+01
7	5186324	9.26E+01
68	mebhydrolin	5.23E-01
95	L-methionine_sulfoximine	3.94E+00
19	disopyramide	6.33E-09
52	naringin	9.83E+01
27	homatropine	5.33E+00
19	sulfapyridine	1.11E-09
19	CAY-10397	1.25E-10
19	caffeic_acid	1.31E-11
83	sulfamonomethoxine	1.17E+01
98	TTNPB	4.02E+00
93	tiapride	9.13E+00
33	carteolol	9.27E+00
96	cyclopenthiazide	3.89E+01
4	butacaine	9.51E+00
90	xylometazoline	4.18E+01
19	PHA-00816795	5.92E-12
22	Prestwick-984	3.12E+01
32	thioguanosine	5.07E+01
86	flucloxacillin	4.66E+00
69	nifuroxazide	2.48E+01
4	pyridoxine	3.32E+00
31	SR-95531	5.14E+01
82	piribedil	6.73E+01
56	ethisterone	2.76E+01
19	AH-6809	3.12E-12
8	6-azathymine	3.07E+01
91	sulindac_sulfide	8.22E+00
90	methylprednisolone	3.91E+01
0	netilmicin	9.57E+01
14	doxorubicin	5.71E+00
33	clebopride	1.67E+00
40	primaquine	1.05E+02

89	dipyridamole	2.55E+01
23	pioglitazone	3.76E+01
72	benzathine_benzylpenicillin	3.45E-02
58	fenbufen	3.38E+00
90	zoxazolamine	3.30E+01
9	BCB000038	9.54E+00
36	levopropoxyphene	1.97E+00
4	hydroflumethiazide	1.57E+00
29	ambroxol	2.07E+00
8	nafcillin	1.15E+01
57	felbinac	1.02E+01
68	piperine	2.57E-01
19	benfluorex	1.21E-11
0	tridihexethyl	9.41E+01
29	tranexamic_acid	6.24E-01
52	edrophonium_chloride	9.32E+01
75	mycophenolic_acid	7.08E+00
91	phenyl_biguanide	1.33E+00
91	tacrolimus	8.32E-02
2	probucol	4.17E+01
97	carbenoxolone	7.22E+01
0	lincomycin	8.81E+01
48	3-hydroxy-DL-kynurenine	3.10E+01
19	genistein	7.59E-12
14	azacitidine	2.75E+00
0	streptozocin	7.84E+01
90	ronidazole	3.86E+01
99	theobromine	8.99E+01
19	dinoprostone	1.63E-12
0	chlormezanone	6.74E+01
90	Prestwick-674	2.90E+01
98	tomelukast	7.52E-01
82	phensuximide	3.91E+01
31	pyrimethamine	3.94E+01
89	tracazolate	2.54E+01
43	naringenin	3.47E+01
90	clorgiline	2.23E+01
90	metamizole_sodium	1.42E+01
3	kaempferol	2.45E+01
60	hexamethonium_bromide	9.74E+01
29	cefotiam	3.75E-01
40	5230742	1.03E+02
0	N-phenylanthranilic_acid	6.36E+01
95	selegiline	3.68E+00
0	pindolol	5.06E+01
36	Prestwick-682	4.26E-01
47	cefixime	2.06E+01
60	tetracycline	7.51E+01
90	nifurtimox	1.25E+01
6	AR-A014418	1.01E+02
49	alpha-estradiol	8.96E+01
58	meclofenoxate	3.27E+00
8	probenecid	6.09E+00
4	decitabine	1.69E+00

79	diphenhydramine	5.76E+01
47	neostigmine_bromide	4.48E+00
65	pirinixic_acid	4.00E+01
27	viomycin	1.14E+01
26	finasteride	6.55E+01
48	12,13-EODE	1.42E+01
31	levocabastine	2.50E+01
0	loracarbef	5.08E+01
40	dienestrol	9.77E+01
90	aminophylline	1.27E+01
1	novobiocin	6.69E+01
73	dexibuprofen	8.27E+01
94	benserazide	4.18E+01
19	pseudopelletierine	6.61E-11
4	molsidomine	6.87E-01
33	prazosin	6.86E-01
0	myosmine	4.45E+01
36	nystatin	4.94E-02
3	hycanthone	1.75E+01
90	ethosuximide	1.07E+01
32	harmine	8.18E+01
9	16-phenyltetranorprostaglandin_E2	3.30E+00
78	arachidonic_acid	4.35E+01
60	guanethidine	5.67E+01
100	diphenylpyraline	1.07E+02
23	SB-203580	1.34E+01
19	pipemidic_acid	4.22E-11
19	AH-23848	5.01E-12
42	chlortetracycline	7.85E+01
29	iopamidol	3.92E-01
52	cinoxacin	9.61E+01
8	fusaric_acid	2.56E+00
82	nabumetone	2.42E+01
15	pilocarpine	5.58E+01
0	hymecromone	4.56E+01
0	pentetic_acid	3.40E+01
39	ribostamycin	6.50E+01
99	canavanine	7.71E+01
0	10-methoxyharmalan	2.52E+01
37	diethylcarbamazine	6.86E+01
0	captopril	1.75E+01
105	conessine	4.25E+00
55	estropipate	6.62E+01
74	naproxen	1.00E+02
32	ethotoin	7.62E+01
90	mevalolactone	1.54E+01
52	bemegride	8.67E+01
44	dopamine	5.84E+01
27	pheneticillin	9.26E+00
19	glycocholic_acid	9.86E-12
90	sulfafurazole	1.13E+01
19	propantheline_bromide	1.27E-12
8	pronetalol	7.61E-01
48	(-)-catechin	6.91E+00

19	AG-012559	1.75E-13
90	methapyrilene	8.05E+00
22	chlorambucil	4.18E+01
102	fluvastatin	1.03E+02
29	sulmazole	2.40E-01
11	PHA-00745360	8.22E+01
16	rosiglitazone	1.03E+02
62	tribenoside	1.07E+02
80	pentoxifylline	6.98E+01
49	dexverapamil	7.49E+01
27	tolnaftate	4.36E+00
48	demecolcine	1.75E+00
14	camptothecin	5.54E+00
58	levonorgestrel	4.62E+00
52	betahistine	7.75E+01
9	11-deoxy-16,16-dimethylprostaglandin_E2	6.49E-01
29	nitrofural	7.92E-02
42	hydrastinine	6.34E+01
29	boldine	1.62E-02
58	dorzolamide	1.60E+00
65	celecoxib	1.10E+01
83	doxycycline	1.58E+01
90	furaltadone	9.93E+00
40	5253409	1.01E+02
16	spironolactone	9.27E+01
96	sulfaguanidine	2.88E+01
27	etiocholanolone	1.88E+00
90	prasterone	7.09E+00
96	tolazoline	6.62E+00
43	diethylstilbestrol	2.98E+01
90	sulfinpyrazone	4.59E+00
25	mefenamic_acid	9.67E+01
65	imatinib	9.62E-01
74	hydralazine	8.84E+01
32	sulfametoxydiazine	7.96E+01
47	cyanocobalamin	2.17E+00
8	tolazamide	2.85E-01
90	oxybuprocaine	3.39E+00
37	dimenhydrinate	5.22E+01
21	PNU-0230031	4.05E+01
99	atovaquone	6.49E+01
94	W-13	1.27E+01
14	fisetin	2.84E+00
13	hydroxyachillin	1.07E+02
5	aminoglutethimide	1.07E+02
24	U0125	5.60E+01
0	pheniramine	4.51E+01
29	furosemide	8.63E-03
102	dioxybenzone	9.62E+01
48	paclitaxel	5.50E-01
10	BCB000039	2.31E+01
69	dobutamine	4.20E+01
37	fenofibrate	2.91E+01

19	PF-00562151-00	1.25E-11
0	isocorydine	3.92E+01
83	pyrithyldione	5.36E+00
14	mitoxantrone	9.76E-01
49	mepyramine	5.76E+01
100	fluoxetine	1.07E+02
0	cyclic_adenosine_monophosphate	3.17E+01
37	bupivacaine	1.26E+01
68	lumicolchicine	1.38E+00
14	alsterpaullone	2.40E-01
42	ajmaline	5.07E+01
8	phenacetin	7.25E-02
68	7-aminocephalosporanic_acid	2.38E-01
3	5211181	2.93E+01
4	bromperidol	2.93E+00
33	nomegestrol	3.70E-01
44	quinpirole	2.56E+01
41	roxarsone	9.78E+01
1	arachidonyltrifluoromethane	4.69E+01
0	yohimbine	2.97E+01
67	tocainide	1.00E+02
0	cyclopentolate	2.20E+01
60	josamycin	6.60E+01
104	5194442	1.06E+02
81	biperiden	8.05E+01
94	tioguanine	1.54E+00
0	Prestwick-1103	1.68E+01
82	alfaxalone	2.27E+01
99	spectinomycin	4.97E+01
88	androsterone	8.75E+01
72	harpagoside	7.38E-01
89	solanine	8.02E+01
30	suprofen	2.78E+01
0	PF-00539745-00	1.33E+01
90	pentoxyverine	1.13E+01
29	ceforanide	6.70E-03
6	Prestwick-675	1.04E+02
40	dicycloverine	1.03E+02
19	mebeverine	7.98E-11
79	heptaminol	4.03E+01
90	lorglumide	8.54E+00
52	cisapride	8.78E+01
55	metoprolol	4.08E+01
77	nimodipine	2.93E+01
32	pancuronium_bromide	8.88E+01
35	articaine	9.99E+01
16	diprophylline	8.86E+01
46	gabapentin	6.22E+01
0	dizocilpine	1.30E+01
100	fluvoxamine	1.07E+02
33	coralyne	3.41E-02
13	ifosfamide	1.07E+02
84	butamben	8.40E+01
19	glafenine	4.00E-11

60	acebutolol	5.21E+01
90	promethazine	8.46E+00
89	altizide	7.41E+01
0	harmalol	1.08E+01
30	Prestwick-864	6.61E+00
61	piracetam	1.07E+02
44	indometacin	6.72E+00
58	retrorsine	3.37E+00
19	PHA-00767505E	1.03E-11
31	amprolium	5.49E+01
52	palmatine	8.08E+01
57	vancomycin	1.39E+01
28	fulvestrant	8.56E+01
56	dantrolene	1.99E+01
13	lidoflazine	1.05E+02
79	betulin	1.43E+01
56	vincamine	2.29E+00
52	dimethadione	6.73E+01
32	vidarabine	8.63E+01
90	pyrazinamide	9.13E+00
95	tobramycin	1.27E+01
99	theophylline	3.82E+01
31	minaprine	3.65E+01
41	imidurea	8.28E+01
32	apigenin	7.60E+01
90	amiodarone	6.86E+00
68	carbarsone	1.14E-01
58	procyclidine	1.55E+00
106	naftopidil	1.02E+02
67	epitiostanol	8.90E+01
40	tyrphostin_AG-1478	1.03E+02
0	midodrine	1.48E+01
83	cefoxitin	2.64E+00
31	cimetidine	2.13E+01
74	lisinopril	8.82E+01
16	valproic_acid	7.86E+01
0	lidocaine	1.11E+01
6	aceclofenac	1.02E+02
89	cantharidin	7.23E+01
90	phthalylsulfathiazole	6.55E+00
19	cotinine	2.40E-11
6	5149715	9.23E+01
13	cefuroxime	1.03E+02
90	famprofazone	4.52E+00
32	chrysin	7.01E+01
80	meclofenamic_acid	5.05E+01
40	PHA-00846566E	1.00E+02
61	gliclazide	1.06E+02
52	doxazosin	6.22E+01
2	3-aminobenzamide	3.79E+01
50	sulfadimidine	9.86E+01
52	Prestwick-860	4.64E+01
78	oligomycin	2.38E+01
80	cloxacillin	1.83E+01

41	allantoin	5.95E+01
19	deptropine	9.69E-12
90	eucatropine	4.17E+00
31	salsolidin	1.28E+01
90	omeprazole	2.60E+00
42	glibenclamide	5.59E+01
61	oleandomycin	1.04E+02
27	thiamphenicol	7.90E+00
45	copper_sulfate	7.24E+01
90	tropicamide	1.76E+00
42	clopamide	3.45E+01
25	atracurium_besilate	9.36E+01
26	thioperamide	6.58E+01
99	galantamine	2.82E+01
31	doxylamine	5.98E+00
0	sulfamethizole	1.69E+01
21	PNU-0293363	3.12E+01
13	protoveratrine_A	1.01E+02
9	IC-86621	3.58E-01
3	5279552	4.42E+01
89	verteporfin	7.06E+01
23	SC-19220	1.09E+01
27	dapsone	3.72E+00
50	piperacillin	7.72E+01
90	fluocinonide	1.73E+00
52	crotamiton	4.08E+01
18	PF-01378883-00	7.44E+01
12	carisoprodol	7.78E+01
85	estriol	1.05E+02
67	indoprofen	7.41E+01
99	mestranol	1.52E+01
57	2-aminobenzenesulfonamide	4.68E+00
22	homosalate	6.45E+01
89	methyldopa	6.01E+01
4	monocrotaline	9.61E+00
69	(+)-isoprenaline	4.73E+01
82	Prestwick-1083	2.30E+01
54	bacitracin	9.32E+01
53	vanoxerine	9.79E+01
71	mephenytoin	1.01E+02
61	Prestwick-559	1.01E+02
4	artemisinin	4.36E+00
5	topiramate	1.07E+02
0	metoclopramide	2.18E+01
90	cetirizine	2.05E+00
0	aconitine	1.59E+01
89	dacarbazine	4.95E+01
0	cytisine	1.12E+01
19	orlistat	6.50E-11
77	oxybenzone	1.29E+01
6	estradiol	9.01E+01
19	dehydrocholic_acid	7.66E-12
90	cortisone	1.54E+00
5	methocarbamol	1.07E+02

3	5255229	3.51E+01
89	phentolamine	3.78E+01
79	ozagrel	5.74E+00
8	oxamniquine	2.13E-01
14	H-7	1.33E+00
67	ticarcillin	5.27E+01
49	kanamycin	7.09E+01
27	isoxicam	2.38E+00
48	5252917	1.27E+00
0	tetracaine	1.11E+01
90	piperidolate	1.34E+00
48	DL-PPMP	1.67E-01
93	griseofulvin	3.94E+01
69	amiloride	2.85E+01
97	tenoxicam	9.53E+01
40	isotretinoin	1.04E+02
27	merbromin	8.26E-01
5	pivmecillinam	1.06E+02
73	orciprenaline	9.57E+01
45	oxamic_acid	3.20E+01
0	megestrol	9.70E+00
58	phenformin	3.01E+00
32	rimexolone	8.80E+01
7	zidovudine	1.07E+02
86	rilmenidine	1.53E+01
90	trazodone	1.30E+00
82	flavoxate	1.25E+01
0	baclofen	7.53E+00
37	chenodeoxycholic_acid	2.75E+01
32	metyrapone	7.93E+01
19	dipivefrine	1.12E-11
41	Prestwick-920	4.78E+01
79	asiaticoside	7.61E-01
101	pentolonium	1.01E+02
37	prednisolone	1.24E+01
88	ketoconazole	7.77E+01
95	seneciphylline	1.37E+01
89	sotalol	3.68E+01
0	dihydrostreptomycin	6.47E+00
31	isoxsuprine	6.75E+00
76	acenocoumarol	9.72E+01
74	roxithromycin	9.02E+01
27	isometheptene	3.48E-01
100	5666823	1.07E+02
42	danazol	3.58E+01
29	amikacin	2.11E-01
43	ciclosporin	5.09E+01
59	fludroxycortide	1.06E+02
90	clobetasol	1.71E+00
95	trimethadione	4.74E+00
72	fluticasone	2.47E+00
49	midecamycin	5.63E+01
100	thioproperazine	1.07E+02
90	bezafibrate	1.13E+00

89	ginkgolide_A	2.93E+01
19	zaprinast	7.09E-12
13	ramipril	1.03E+02
55	kinetin	3.24E+01
27	ketotifen	1.03E-01
0	nizatidine	7.36E+00
85	ursodeoxycholic_acid	1.02E+02
32	luteolin	7.86E+01
77	nitrendipine	2.61E+00
38	khellin	1.02E+02
83	iopromide	1.91E+00
101	trimethoprim	8.18E+01
43	saquinavir	2.82E+01
0	dexamethasone	5.78E+00
66	karakoline	9.32E+01
13	methacholine_chloride	9.95E+01
40	ionomycin	1.05E+02
0	chlorpropamide	3.97E+00
52	lomefloxacin	6.26E+01
18	valdecoxib	3.83E+01
27	carbimazole	2.47E-02
24	splitomicin	3.85E+01
71	chloropyrazine	9.10E+01
29	primidone	1.03E-01
61	budesonide	1.02E+02
7	diflunisal	1.07E+02
27	isoflupredone	3.46E-03
89	laudanosine	2.56E+01
32	acacetin	7.39E+01
100	clemastine	1.07E+02
74	proxyphylline	8.06E+01
25	metrifonate	9.14E+01
52	dydrogesterone	5.18E+01
31	pralidoxime	4.79E+00
19	erythromycin	7.34E-12
40	propidium_iodide	1.03E+02
100	thiethylperazine	1.07E+02
6	etodolac	9.36E+01
89	desoxycortone	1.80E+01
29	metronidazole	2.98E-02
0	folic_acid	4.83E+00
102	meglumine	1.06E+02
52	piromidic_acid	3.99E+01
106	zardaverine	1.01E+02
97	(+/-)-catechin	8.67E+01
54	trapidil	7.53E+01
11	CP-320650-01	9.33E+01
17	monastrol	8.67E+01
7	melatonin	1.07E+02
34	proadifen	1.07E+02
42	chlorzoxazone	3.03E+01
82	remoxipride	8.39E+00
96	succinylsulfathiazole	1.19E+01
19	lobelanidine	1.99E-12

61	nisoxetine	9.89E+01
14	ellipticine	1.87E+00
42	betaxolol	1.56E+01
13	bupropion	9.78E+01
0	cefepime	5.03E+00
76	neomycin	7.71E+01
70	vinblastine	3.82E+00
0	praziquantel	3.31E+00
32	sulfaphenazole	7.40E+01
88	domperidone	5.71E+01
0	0225151-0000	2.11E+00
51	HNMPA-(AM)3	8.82E+01
99	colistin	2.50E+01
0	etynodiol	1.30E+00
53	cephaeline	8.84E+01
6	debrisoquine	8.68E+01
0	PF-00539758-00	7.70E-01
1	2-deoxy-D-glucose	5.84E+01
41	octopamine	3.91E+01
80	mefexamide	1.35E+01
32	tiabendazole	6.56E+01
16	fusidic_acid	9.76E+01
0	Prestwick-857	5.00E-01
10	15(S)-15-methylprostaglandin_E2	2.56E+01
43	MK-886	1.75E+01
34	chlorphenamine	1.07E+02
67	sulfaquinoxaline	5.32E+01
6	metformin	7.37E+01
0	cromoglicic_acid	3.17E-01
74	fenspiride	7.19E+01
0	sulfabenzamide	1.64E-01
46	zomepirac	7.41E+01
62	antimycin_A	1.07E+02
22	lithocholic_acid	7.61E+01
106	molindone	9.21E+01
99	arcaine	1.39E+01
100	S-propranolol	1.07E+02
89	ipratropium_bromide	2.17E+01
88	procainamide	2.90E+01
12	Chicago_Sky_Blue_6B	7.54E+01
7	triamcinolone	1.07E+02
32	lymecycline	6.06E+01
61	triflusal	9.57E+01
3	5109870	5.94E+01
3	trifluridine	4.02E+01
81	epiandrosterone	8.20E+01
13	physostigmine	9.63E+01
5	convolamine	1.07E+02
40	econazole	1.05E+02
39	isopropamide_iodide	8.25E+01
40	Y-27632	1.02E+02
100	proguanil	1.07E+02
4	thiamine	1.85E+01
58	flumetasone	8.08E+00

38	guanadrel	9.23E+01
74	phenindione	5.60E+01
72	3-acetamidocoumarin	2.29E+00
22	nialamide	5.80E+01
29	canadine	3.30E-02
68	carcinine	7.48E-01
31	idazoxan	5.11E+00
61	tubocurarine_chloride	8.80E+01
52	meropenem	5.10E+01
87	lansoprazole	1.06E+02
100	BW-B70C	1.07E+02
98	tretinoin	5.68E+00
35	atropine_methonitrate	1.04E+02
31	sulfathiazole	1.78E+00
32	nifedipine	5.80E+01
25	sulfacetamide	8.57E+01
90	medrysone	2.42E+01
69	oxedrine	3.64E+01
26	timolol	6.74E+01
6	prednisone	6.78E+01
0	naphazoline	5.17E-01
4	metanephrine	1.16E+01
32	todralazine	4.75E+01
89	hydrocotarnine	2.19E+01
16	trichostatin_A	9.43E+01
5	levothyroxine_sodium	1.07E+02
43	equilin	8.72E+00
99	simvastatin	8.57E+00
6	corticosterone	5.10E+01
49	4,5-dianilinophthalimide	6.39E+01
60	bambuterol	9.88E+01
17	thalidomide	5.28E+01
100	amoxapine	1.07E+02
82	procaine	5.58E+00
68	lactobionic_acid	9.71E-02
32	metacycline	3.93E+01
106	zalcitabine	7.98E+01
90	pipenzolate_bromide	2.63E+01
90	alverine	1.95E+01
73	diltiazem	9.77E+01
90	norethisterone	1.42E+01
60	famotidine	9.01E+01
87	ioxaglic_acid	1.01E+02
100	troglitazone	1.07E+02
41	niridazole	2.83E+01
73	alfuzosin	8.13E+01
96	minoxidil	3.57E+00
90	cycloserine	1.14E+01
106	meptazinol	5.58E+01
84	ramifenazone	8.82E+01
22	quinethazone	4.44E+01
5	azathioprine	1.06E+02
27	Prestwick-692	1.03E-02
32	harman	3.34E+01

90	meclozine	8.93E+00
52	rolitetracycline	5.24E+01
18	3-nitropropionic_acid	1.46E+01
97	tolmetin	8.31E+01
104	phenoxybenzamine	1.07E+02
21	0173570-0000	4.03E+01
42	methazolamide	1.98E+01
0	hemicholinium	1.27E+00
7	benzydamine	1.07E+02
67	gibberellic_acid	4.57E+01
48	chloroquine	2.95E-01
90	ifenprodil	7.68E+00
7	Prestwick-1082	1.07E+02
90	capsaicin	5.09E+00
90	denatonium_benzoate	3.14E+00
74	acetohexamide	5.02E+01
52	pivampicillin	4.22E+01
16	idoxuridine	8.92E+01
7	5151277	1.06E+02
0	nifenazone	1.04E+00
52	ioversol	2.92E+01
100	promazine	1.07E+02
106	etilefrine	3.38E+01
8	sisomicin	1.06E+00
62	mifepristone	1.07E+02
90	adrenosterone	2.59E+00
101	riluzole	7.67E+01
49	verapamil	5.15E+01
0	pregnenolone	7.53E-01
42	dirithromycin	1.05E+01
90	latamoxef	1.72E+00
49	racecadotril	2.91E+01
90	etanidazole	9.97E-01
4	canrenoic_acid	1.15E+01
43	pizotifen	3.54E+00
6	naltrexone	4.91E+01
21	cefamandole	1.63E+01
4	carbamazepine	4.95E+00
69	colchicine	2.67E+01
43	estrone	4.30E-01
27	clorsulon	1.68E-03
74	nipecotic_acid	3.44E+01
31	corynanthine	1.74E+00
85	milrinone	1.04E+02
104	semustine	1.07E+02
90	anabasine	8.49E-01
60	chlorphenesin	8.72E+01
35	tiletamine	1.02E+02
100	procarbazine	1.07E+02
52	natamycin	2.49E+01
90	difenidol	5.22E-01
0	antazoline	9.17E-01
36	gabexate	3.03E+00
32	skimmianine	4.14E+01

73	hesperidin	6.77E+01
87	prednicarbate	9.14E+01
22	levcycloserine	3.41E+01
90	(-)-MK-801	3.39E-01
102	(-)-atenolol	1.07E+02
103	dicloxacillin	9.78E+01
40	vinburnine	1.06E+02
90	riboflavin	1.87E-01
6	hexylcaine	3.75E+01
82	iopanoic_acid	2.89E+00
3	blebbistatin	5.75E+01
0	nicardipine	7.93E-01
90	bretylium_tosilate	1.04E-01
0	quipazine	4.22E-01
99	fosfosal	8.30E+00
35	alfadolone	9.19E+01
58	fenoprofen	1.44E+01
100	lovastatin	1.07E+02
61	etamivan	9.67E+01
100	phenazopyridine	1.07E+02
66	pramocaine	8.40E+01
8	ethambutol	2.17E-01
0	ergocalciferol	2.89E-01
32	flunixin	3.76E+01
10	CP-319743	1.15E+01
61	thiocolchicoside	8.73E+01
25	dexpanthenol	8.44E+01
90	flunarizine	9.06E-02
0	N6-methyladenosine	1.69E-01
64	aztreonam	1.06E+02
61	ceftazidime	7.35E+01
19	16,16-dimethylprostaglandin_E2	9.02E-08
22	santonin	1.95E+01
71	ciprofibrate	9.45E+01
82	scopolamine_N-oxide	4.54E-01
7	cefotetan	1.06E+02
20	sulfachlorpyridazine	1.06E+02
13	testosterone	1.05E+02
7	cefapirin	1.05E+02
20	0317956-0000	1.02E+02
93	nalidixic_acid	5.50E+01
15	diclofenamide	8.61E+01
46	pridinol	7.32E+01
61	iobenguane	5.89E+01
47	bacampicillin	2.30E+01
5	azlocillin	1.07E+02
44	cobalt_chloride	1.29E+01
12	meclocycline	7.41E+01
31	gemfibrozil	1.06E+00
60	gefitinib	8.38E+01
32	acetylsalicylic_acid	3.50E+01
31	cefotaxime	1.81E-01
35	Prestwick-972	7.73E+01
90	ranitidine	1.30E-01

64	pyrantel	9.74E+01
13	tomatidine	1.03E+02
86	adipiodone	1.36E+01
76	pirenzepine	7.27E+01
52	cinchocaine	3.05E+01
22	Prestwick-1100	8.79E+00
97	tetroquinone	7.86E+01
34	rescinnamine	1.07E+02
5	bergenin	1.06E+02
26	aciclovir	5.89E+01
85	repaglinide	1.01E+02
6	hydroxyzine	3.43E+01
50	dicoumarol	9.73E+01
52	progesterone	2.04E+01
14	MS-275	1.05E+01
19	ornidazole	4.98E-08
37	niflumic_acid	4.93E+01
35	metrizamide	5.40E+01
58	tetryzoline	1.15E+01
99	reserpine	4.92E+00
102	biotin	1.07E+02
81	propranolol	7.52E+01
61	Trolox_C	4.94E+01
29	trihexyphenidyl	3.44E-01
93	ofloxacin	2.73E+01
53	emetine	9.00E+01
41	chloramphenicol	2.80E+01
93	flutamide	5.83E+00
0	alclometasone	7.32E-01
64	hyoscyamine	7.63E+01
20	PHA-00851261E	9.06E+01
90	sulfamerazine	2.39E-01
55	napelline	3.99E+01
78	clofibrate	2.56E+01
2	bucladesine	5.49E+01
0	pirlindole	4.60E-01
0	demecarium_bromide	2.16E-01
0	benzbromarone	9.50E-02
40	(+)-chelidonine	1.07E+02
89	etamsylate	7.13E+01
61	picotamide	3.55E+01
90	cyproterone	1.63E-01
7	noscapine	1.05E+02
104	lomustine	1.07E+02
40	nalbuphine	1.07E+02
4	5114445	9.05E+00
61	propafenone	2.05E+01
39	carbinoxamine	7.88E+01
74	imipenem	3.95E+01
61	trimetazidine	9.53E+00
90	Prestwick-1084	1.03E-01
100	flupentixol	1.07E+02
90	liothyronine	4.50E-02
57	eticlopride	1.11E+01

75	rifampicin	5.78E+01
100	norcyclobenzaprine	1.07E+02
2	4-hydroxyphenazone	1.78E+01
6	5186223	2.81E+01
90	scopoletin	2.26E-02
89	terbutaline	6.42E+01
0	ascorbic_acid	1.12E-01
37	bumetanide	3.48E+01
40	mianserin	1.06E+02
60	1,5-isoquinolinediol	8.27E+01
61	cyclizine	4.18E+00
75	ribavirin	1.85E+01
45	tetraethylenepentamine	2.88E+01
0	chloropyramine	6.09E-02
87	risperidone	8.53E+01
99	trichlormethiazide	2.09E+00
90	parbendazole	1.49E-02
35	enilconazole	3.60E+01
100	sirolimus	1.07E+02
31	oxetacaine	6.97E-02
52	xylazine	2.46E+01
72	memantine	6.67E+00
37	dropropizine	1.68E+01
85	benzylpenicillin	9.75E+01
29	vigabatrin	1.70E-01
100	monensin	1.07E+02
67	Prestwick-967	5.70E+01
37	telenzepine	5.01E+00
25	moxisylyte	8.25E+01
100	clozapine	1.07E+02
89	oxytetracycline	5.88E+01
74	norfloxacin	2.62E+01
32	dextromethorphan	5.86E+01
0	stachydrine	6.04E-02
87	spaglumic_acid	6.02E+01
16	rifabutin	1.01E+02
34	dosulepin	1.07E+02
4	syrosingopine	6.17E+00
22	ciclacillin	5.65E+00
47	sparteine	9.36E+00
41	etofylline	1.54E+01
87	bethanechol	2.85E+01
62	isradipine	1.07E+02
100	pimozide	1.07E+02
41	torasemide	2.90E+00
7	piroxicam	1.06E+02
13	sulfadiazine	1.06E+02
60	tolfenamic_acid	7.44E+01
85	etomidate	8.58E+01
97	amylocaine	7.17E+01
85	oxprenolol	6.24E+01
76	acetylsalicylsalicylic_acid	5.13E+01
97	furazolidone	4.30E+01
17	NU-1025	3.33E+01

67	cefalonium	3.49E+01
62	foliosidine	1.07E+02
34	nicergoline	1.07E+02
6	tetrahydroalstonine	2.38E+01
58	sulfamethoxypyridazine	1.34E+01
72	decamethonium_bromide	1.56E+00
58	Prestwick-685	4.62E+00
100	dihydroergocristine	1.07E+02
46	isoetarine	6.55E+01
74	meticrane	1.42E+01
90	sulconazole	6.29E-02
90	fluorometholone	2.31E-02
12	brompheniramine	6.62E+01
60	vinpocetine	5.94E+01
32	tinidazole	6.00E+01
89	R-atenolol	5.88E+01
25	acetazolamide	6.68E+01
11	spiradoline	1.04E+02
40	prilocaine	1.07E+02
42	Prestwick-689	2.94E+01
5	cinchonidine	1.07E+02
0	sulfanilamide	1.68E-01
100	raubasine	1.07E+02
16	LY-294002	9.86E+01
21	mephenesin	1.22E+01
64	oxolinic_acid	5.59E+01
81	pirenperone	5.61E+01
90	pentamidine	1.77E-02
35	aminocaproic_acid	2.08E+01
25	triflupromazine	4.10E+01
52	cefsulodin	3.04E+01
104	carmustine	1.07E+02
100	co-dergocrine_mesilate	1.07E+02
85	azaperone	4.19E+01
66	betonicine	7.06E+01
5	bisoprolol	1.07E+02
102	naftidrofuryl	1.07E+02
100	amphotericin_B	1.07E+02
89	pargyline	5.20E+01
59	pempidine	1.07E+02
39	butirosin	5.85E+01
14	daunorubicin	1.53E+01
80	iproniazid	1.93E+01
13	flufenamic_acid	1.06E+02
53	cicloheximide	8.20E+01
100	CP-645525-01	1.07E+02
62	ticlopidine	1.07E+02
52	bendroflumethiazide	2.15E+01
60	nimesulide	4.72E+01
40	betazole	1.07E+02
54	esculetin	9.19E+01
60	SC-58125	2.64E+01
32	heliotrine	6.23E+01
49	mesalazine	5.90E+01

102	urapidil	1.07E+02
20	proglumide	8.36E+01
34	dihydroergotamine	1.07E+02
54	Prestwick-981	6.14E+01
83	clidinium_bromide	1.09E+01
1	fasudil	8.42E+01
90	tranylcypromine	3.30E-02
88	ritodrine	5.09E+01
92	bicuculline	1.07E+02
50	ethoxyquin	9.70E+01
13	scopolamine	1.05E+02
102	nicotinic_acid	1.06E+02
32	paroxetine	5.34E+01
106	alprostadil	5.81E+01
16	vorinostat	9.52E+01
62	mercaptopurine	1.07E+02
89	flumequine	4.88E+01
7	cinnarizine	1.06E+02
59	solasodine	1.07E+02
5	haloperidol	1.07E+02
90	hexestrol	2.16E-02
100	lasalocid	1.07E+02
100	cyclobenzaprine	1.07E+02
32	proxymetacaine	4.29E+01
52	fluorocurarine	1.72E+01
61	fipexide	7.82E+00
38	sulfadimethoxine	1.02E+02
34	aminophenazone	1.07E+02
7	triprolidine	1.06E+02
62	butoconazole	1.07E+02
89	sitosterol	3.79E+01
6	5152487	2.57E+01
100	ivermectin	1.07E+02
0	propoxycaine	1.17E+00
20	indapamide	5.60E+01
62	clioquinol	1.07E+02
40	lynestrenol	1.07E+02
95	moxonidine	5.55E+01
95	propylthiouracil	2.57E+01
0	piretanide	6.47E-01
49	exemestane	4.18E+01
42	clindamycin	2.54E+01
4	ikarugamycin	9.88E+00
19	STOCK1N-35696	7.01E-05
101	clemizole	8.95E+01
58	aminohippuric_acid	4.46E+00
3	colforsin	9.96E+01
13	demeclocycline	1.04E+02
92	terconazole	1.07E+02
5	minocycline	1.06E+02
11	dilazep	1.00E+02
7	tiratricol	1.05E+02
32	2,6-dimethylpiperidine	4.06E+01
106	Prestwick-983	3.47E+01

40	pimethixene	1.07E+02
85	sulfadoxine	2.89E+01
13	hydrastine_hydrochloride	1.02E+02
100	ursolic_acid	1.07E+02
90	apramycin	5.01E-02
52	esculin	1.39E+01
3	ciclopirox	9.27E+01
52	ethaverine	5.81E+00
73	bromocriptine	9.26E+01
60	wortmannin	2.21E+01
97	paromomycin	3.70E+01
3	resveratrol	7.91E+01
73	methylergometrine	7.03E+01
92	vitexin	1.07E+02
100	imipramine	1.07E+02
1	sodium_phenylbutyrate	6.29E+01
90	acepromazine	2.74E-02
7	mecamylamine	1.03E+02
105	gelsemine	7.33E+01
104	C-75	1.07E+02
87	moracizine	1.81E+01
34	atropine_oxide	1.07E+02
1	tamoxifen	2.36E+01
29	cinchonine	5.31E-01
0	thiamazole	1.37E+00
7	buspirone	9.88E+01
32	etidronic_acid	3.76E+01
7	amrinone	9.00E+01
5	cefazolin	1.06E+02
62	erastin	1.07E+02
81	serotonin	3.16E+01
37	enalapril	5.74E+00
59	alcuronium_chloride	1.07E+02
100	thioridazine	1.07E+02
100	amitriptyline	1.07E+02
54	halofantrine	3.24E+01
32	trioxysalen	2.66E+01
12	SR-95639A	5.85E+01
100	clofilium_tosylate	1.07E+02
102	flurbiprofen	1.06E+02
89	sanguinarine	4.82E+01
88	clenbuterol	2.64E+01
22	phenelzine	6.53E+00
0	glimepiride	1.26E+00
102	rolipram	1.04E+02
5	noretynodrel	1.05E+02
62	miconazole	1.07E+02
84	nadide	1.01E+02
59	citiolone	1.05E+02
38	atractyloside	9.62E+01
71	ondansetron	1.03E+02
32	Prestwick-665	1.88E+01
26	lisuride	7.03E+01
62	naftifine	1.07E+02

6	diphemanil_metilsulfate	2.59E+01
42	chlorhexidine	1.84E+01
60	amodiaquine	1.32E+01
7	ricinine	8.55E+01
40	PF-00875133-00	1.07E+02
6	calycanthine	9.72E+00
62	LM-1685	1.07E+02
39	drofenine	3.67E+01
5	AG-028671	1.04E+02
63	digitoxigenin	1.07E+02
62	clotrimazole	1.07E+02
38	glycopyrronium_bromide	7.29E+01
84	leflunomide	8.14E+01
49	troleandomycin	3.31E+01
67	mephentermine	4.44E+01
34	isoconazole	1.07E+02
0	ampicillin	1.59E+00
95	tiaprofenic_acid	1.37E+01
40	cyproheptadine	1.07E+02
62	azacyclonol	1.07E+02
90	tyloxapol	1.76E-01
0	brinzolamide	7.59E-01
3	deferoxamine	8.02E+01
24	docosahexaenoic_acid_ethyl_ester	5.89E+01
89	talampicillin	4.72E+01
51	butein	9.80E+01
59	flunisolide	1.01E+02
97	iodixanol	1.84E+01
74	morantel	2.81E+01
89	tolbutamide	3.05E+01
103	velnacrine	9.83E+01
50	benfotiamine	9.56E+01
50	spiramycin	6.96E+01
16	trimethobenzamide	1.02E+02
90	colecalciferol	1.02E-01
35	gliquidone	1.96E+01
71	Prestwick-1085	9.62E+01
104	F0447-0125	1.07E+02
100	tetrandrine	1.07E+02
58	3-acetylcoumarin	3.81E+00
16	mepacrine	9.49E+01
62	rotenone	1.07E+02
13	clonidine	1.05E+02
0	nefopam	7.27E-01
102	phenylpropanolamine	1.03E+02
11	alpha-ergocryptine	9.73E+01
40	1,4-chrysenequinone	1.07E+02
13	gramine	1.03E+02
63	strophanthidin	1.07E+02
92	tetramisole	1.07E+02
13	DL-thiorphan	9.75E+01
11	citalopram	7.40E+01
91	sulindac	4.52E+01
71	tropine	7.45E+01

100	orphenadrine	1.07E+02
4	pinacidil	1.67E+01
7	droperidol	8.79E+01
34	penbutolol	1.07E+02
49	rofecoxib	1.43E+01
34	mefloquine	1.07E+02
104	STOCK1N-35215	1.07E+02
5	ciprofloxacin	1.05E+02
25	benzonatate	5.80E+01
50	flecainide	3.43E+01
32	lysergol	3.07E+01
100	metergoline	1.07E+02
34	spiperone	1.06E+02
34	prenylamine	1.03E+02
5	betamethasone	1.02E+02
16	bufexamac	8.64E+01
104	etacrynic_acid	1.07E+02
104	myricetin	1.07E+02
7	diazoxide	7.70E+01
0	diloxanide	9.84E-01
32	cefalotin	1.87E+01
92	Prestwick-642	1.05E+02
30	amiprilose	5.85E+01
5	5140203	9.59E+01
89	N-acetyl-L-leucine	3.31E+01
46	ebselen	8.04E+01
3	guaifenesin	7.97E+01
61	oxymetazoline	2.36E+01
0	terazosin	4.46E-01
59	eldeline	9.54E+01
89	6-benzylaminopurine	1.72E+01
62	rottlerin	1.07E+02
40	piperlongumine	1.07E+02
100	calmidazolium	1.07E+02
0	mimosine	1.39E-01
100	fluspirilene	1.07E+02
90	dexpropranolol	5.42E-01
28	monorden	1.07E+02
32	Prestwick-664	1.14E+01
102	altretamine	1.03E+02
3	etoposide	6.03E+01
23	NS-398	6.26E+01
26	guanabenz	4.81E+01
62	valinomycin	1.07E+02
92	ethionamide	1.00E+02
73	fenoterol	7.46E+01
40	dyclonine	1.07E+02
100	perhexiline	1.07E+02
103	methoxsalen	7.29E+01
71	methanthelinium_bromide	4.39E+01
104	parthenolide	1.07E+02
102	flucytosine	9.54E+01
7	BAS-012416453	7.13E+01
25	epirizole	3.10E+01

55	suxibuzone	5.79E+01
90	nitrofurantoin	3.68E-01
12	oxolamine	4.58E+01
101	kawain	9.20E+01
40	MG-132	1.07E+02
5	oxantel	9.42E+01
100	raloxifene	1.07E+02
92	iocetamic_acid	8.38E+01
13	labetalol	1.02E+02
104	15-delta_prostaglandin_J2	1.06E+02
51	dimethyloxalylglycine	7.60E+01
99	trimethylcolchicinic_acid	2.18E+01
14	gallamine_triethiodide	5.52E+01
63	proscillaridin	1.07E+02
3	triamterene	4.20E+01
102	hydrocortisone	8.27E+01
63	bisacodyl	1.07E+02
5	quinidine	8.29E+01
90	beta-escin	1.81E-01
61	mesoridazine	1.26E+01
53	propofol	9.48E+01
100	albendazole	1.07E+02
13	diclofenac	9.72E+01
102	terguride	5.63E+01
100	metitepine	1.07E+02
100	fluphenazine	1.07E+02
63	digoxigenin	1.07E+02
42	lobeline	2.38E+01
104	thiostrepton	1.05E+02
13	tremorine	8.58E+01
100	alimemazine	1.07E+02
11	CP-690334-01	5.45E+01
105	diflorasone	6.64E+01
104	oxyphenbutazone	1.01E+02
66	isosorbide	7.35E+01
4	berberine	1.65E+01
32	0175029-0000	1.37E+01
102	florfenicol	2.47E+01
40	5224221	1.07E+02
100	zuclopenthixol	1.07E+02
34	nordihydroguaiaretic_acid	1.06E+02
14	bromopride	2.85E+01
34	suloctidil	1.01E+02
5	N-acetyl-L-aspartic_acid	7.31E+01
100	prochlorperazine	1.07E+02
13	alpha-yohimbine	7.17E+01
100	clomifene	1.07E+02
20	mexiletine	6.50E+01
32	amoxicillin	4.17E+00
62	profenamine	1.07E+02
7	5162773	7.46E+01
62	pararosaniline	1.07E+02
100	trifluoperazine	1.07E+02
40	thapsigargin	1.07E+02

84	cefmetazole	7.28E+01
13	papaverine	4.72E+01
5	streptomycin	5.03E+01
28	tanespimycin	1.07E+02
101	salbutamol	6.87E+01
16	scriptaid	9.67E+01
7	5213008	5.04E+01
6	tacrine	2.74E+01
100	doxepin	1.07E+02
0	cefoperazone	2.95E+00
52	levamisole	7.26E+01
52	bephenium_hydroxynaphthoate	4.30E+01
100	hexetidine	1.07E+02
0	SC-560	5.52E-01
15	STOCK1N-35874	1.05E+02
90	sertaconazole	5.48E-01
40	puromycin	1.07E+02
38	calcium_folinate	7.32E+01
69	scoulerine	1.05E+02
7	oxaprozin	2.27E+01
16	HC_toxin	8.12E+01
62	pyrvinium	1.07E+02
104	5155877	9.96E+01
34	trimipramine	9.88E+01
62	dequalinium_chloride	1.07E+02
15	Gly-His-Lys	8.53E+01
100	cytochalasin_B	1.07E+02
59	halcinonide	9.85E+01
46	corbadrine	7.32E+01
100	chlorpromazine	1.07E+02
76	nomifensine	8.22E+01
40	MG-262	1.06E+02
100	tonzonium_bromide	1.07E+02
28	alvespimycin	1.05E+02
100	levomepromazine	1.07E+02
104	withaferin_A	8.66E+01
69	mebendazole	9.89E+01
67	letrozole	6.68E+01
100	protriptyline	1.07E+02
100	diperodon	1.07E+02
59	mepenzolate_bromide	7.16E+01
100	nortriptyline	1.07E+02
100	podophyllotoxin	1.07E+02
53	anisomycin	8.31E+01
40	fendiline	1.06E+02
40	mometasone	1.02E+02
60	clomipramine	5.42E+01
74	ketorolac	6.19E+01
16	pergolide	5.79E+01
3	quercetin	5.24E+01
85	delsoline	7.41E+01
92	beclometasone	8.03E+01
100	maprotiline	1.07E+02
100	0297417-0002B	1.07E+02

62	0179445-0000	1.07E+02
89	menadione	7.64E+01
100	benzethonium_chloride	1.07E+02
100	chlorprothixene	1.07E+02
62	5707885	1.07E+02
64	pentetrazol	9.23E+01
62	clofazimine	1.07E+02
89	ketoprofen	4.21E+01
63	digoxin	1.07E+02
106	dinoprost	8.44E+01
100	bepridil	1.07E+02
104	securinine	7.01E+01
73	(-)-isoprenaline	8.62E+01
34	astemizole	1.00E+02
100	cloperastine	1.07E+02
40	disulfiram	1.03E+02
62	benzamil	1.07E+02
63	lycorine	1.07E+02
28	geldanamycin	9.72E+01
34	terfenadine	7.65E+01
100	homochlorcyclizine	1.07E+02
100	piperacetazine	1.07E+02
63	hydroquinine	1.07E+02
100	chlorcyclizine	1.07E+02
62	felodipine	1.07E+02
62	abamectin	1.06E+02
13	atropine	7.69E+01
100	quinisocaine	1.07E+02
100	desipramine	1.07E+02
5	isocarboxazid	8.25E+01
52	carbachol	7.90E+01
100	perphenazine	1.07E+02
69	fenbendazole	9.83E+01
100	methylbenzethonium_chloride	1.07E+02
63	lanatoside_C	1.07E+02
62	niclosamide	1.06E+02
62	gossypol	9.09E+01
40	celastrol	1.06E+02
40	loxapine	9.34E+01
63	ouabain	1.07E+02
100	alexidine	1.07E+02
100	metixene	1.07E+02
100	loperamide	1.01E+02
63	helveticoside	1.07E+02
